# Non-analog environments supported early *Homo sapiens* in southern Africa 200 000 years ago

**DOI:** 10.1038/s43247-026-04116-4

**Published:** 2026-10-30

**Authors:** Michaela Ecker, Maïlys Richard, Nils Andersen, Beatrice Bin, Will Archer, C. Britt Bousman, Josep M. Parés, Andri C. van Aardt, Louis Scott, Michael B. Toffolo

**Affiliations:** 1 Institute of Prehistoric and Protohistoric Archaeology, Kiel University, Kiel, Germany; 2 Archaeology Department, McGregor Museum, Kimberley, South Africa; 3 Archéosciences Bordeaux, UMR 6034 CNRS-Bordeaux Montaigne University, Pessac, France; 4 Department of Early Prehistory and Quaternary Ecology, University of Tübingen, Tübingen, Germany; 5 Leibniz Laboratory for Radiometric Dating and Isotope Research, Kiel University, Kiel, Germany; 6 Geochronology and Geology Programme, Centro Nacional de Investigación sobre la Evolución Humana (CENIEH), Burgos, Spain; 7 Max Planck Partner Group, Department of Archaeology and Anthropology, National Museum, Bloemfontein, South Africa; 8 Florisbad Quaternary Research Station, National Museum, Bloemfontein, South Africa; 9 Department of Geology, University of the Free State, Bloemfontein, South Africa; 10 Department of Anthropology, The George Washington University, Washington, DC USA; 11 Center for Archaeological Studies, Department of Anthropology, Texas State University, San Marcos, TX USA; 12 Department of Plant Sciences, University of the Free State, Bloemfontein, South Africa

**Keywords:** Palaeoecology, Archaeology, Biogeochemistry

## Abstract

In our species’ evolutionary history, the type of environments in which we evolved can provide crucial details about early human adaptive capacities. Such environments might not exist under current climatic and ecological conditions. Reconstructing palaeohydrology and vegetation using multiple proxies at human fossil sites in South Africa provides insights into local conditions at a high level of detail. This paper presents a plant *n*-alkane stable isotope record for Florisbad and Lombard’s Drift, which is anchored by new luminescence ages that revise the age for the Florisbad human cranium. We observe contrasting, non-analog plant environments during the Middle and Late Pleistocene by combining multiple lines of evidence to better understand the different environments that early *Homo sapiens* experienced. It also provides the foundation to evaluate their cultural adaptation strategies through time.

## Abstract (web summary)

Early *Homo sapiens* in South Africa experienced contrasting, non‑analogous environments shaped by shifting hydrology and vegetation, based on plant biomarker isotope records and revised dating of Middle-Pleistocene fossil sites, which provides context for hominin adaptive strategies

## Introduction

**Fig. 1:**
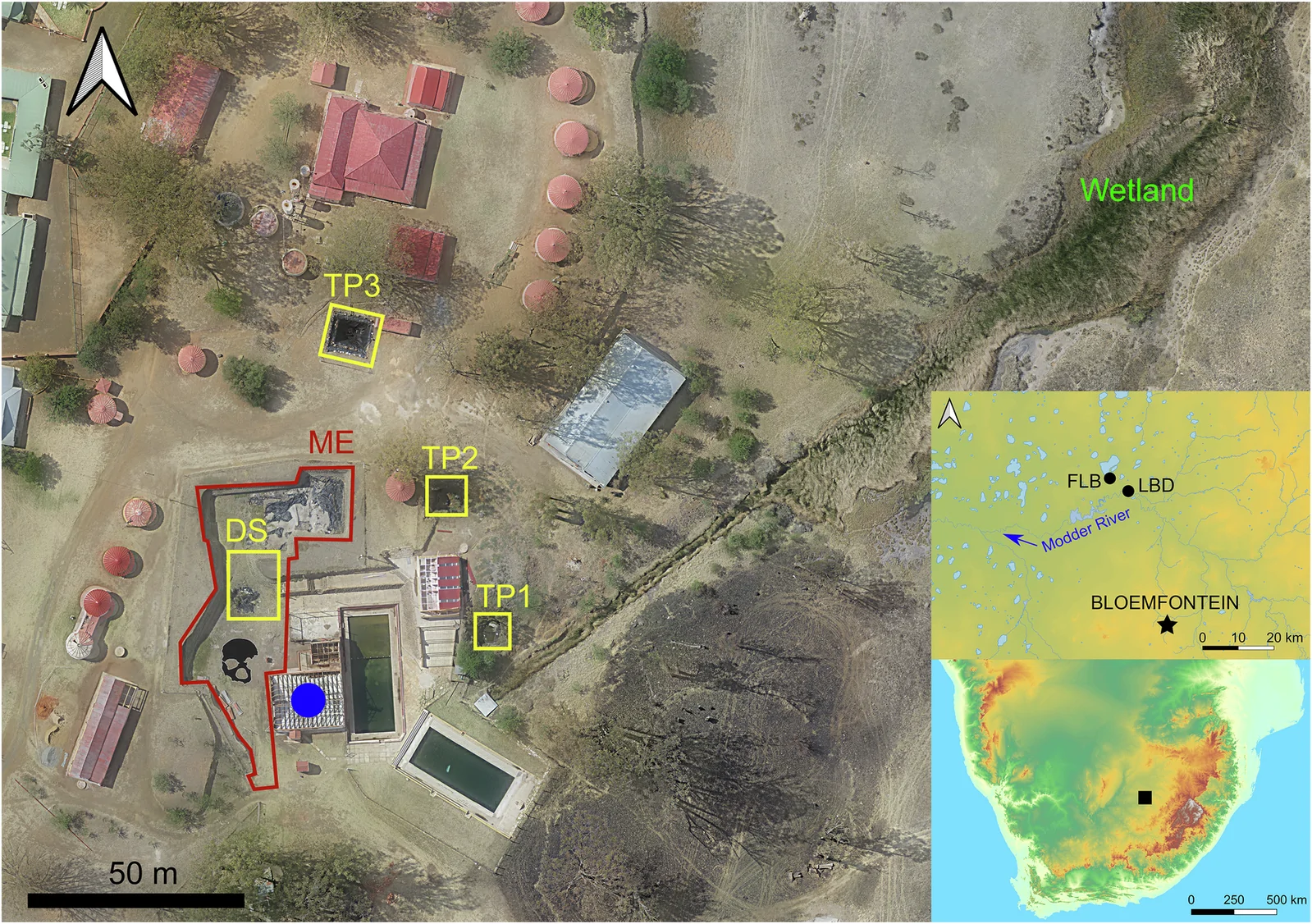
Aerial view of Florisbad, showing the location of excavation areas and the approximate find spot of the human cranium (black silhouette). ME Main Excavation, DS Dreyer Section, TP Test Pit. The blue circle marks the current location of the spring. The insets show the location of Florisbad and Lombard’s Drift in the Free State and the location of the study area in southern Africa. Source for insets is the Shuttle Radar Topography Mission 1-arc second Global, National Geospatial-Intelligence Agency (NGA), USGS Earth Resources Observation and Science (EROS), retrieved from NASA Earthdata.

*Homo sapiens* emerged in a pan-African evolutionary process shortly before 300 thousand years ago (ka)^1,2^. Associated with this emergence are widespread technological and cultural changes that characterise the Middle Stone Age (MSA)^3–7^. Shortly after their appearance, *H. sapiens* seem to have successfully adapted to diverse environmental contexts [e.g., refs. ^8–10^]. These diverse environments include altered contexts with no modern analogues, such as in today’s drylands that during the Pleistocene show phases of wetter climate with increased year-round water availability^11,12^. Non-analogue refers here to environments that do not occur in the modern biomes of South Africa on bio-geochemical grounds and include different climatic regimes and species adaptations which in turn lead to combinations of plant and animal species not found together today. During the Middle Pleistocene, southern Africa was characterised by a changing network of lakes and rivers^13–17^, however, the rarity of human fossils from this period limits our assessment of the influence of shifts in palaeohydrology on human cultural and biological evolution. The site of Florisbad in the Free State Province of South Africa (Fig. 1), provides a unique chance to study vegetation changes and trends related to rainfall regimes from well-preserved peat layers spanning the Middle Pleistocene to Holocene, and includes a partial human cranium^18–22^, as well as several archaeological assemblages^23–26^.

**Fig. 2:**
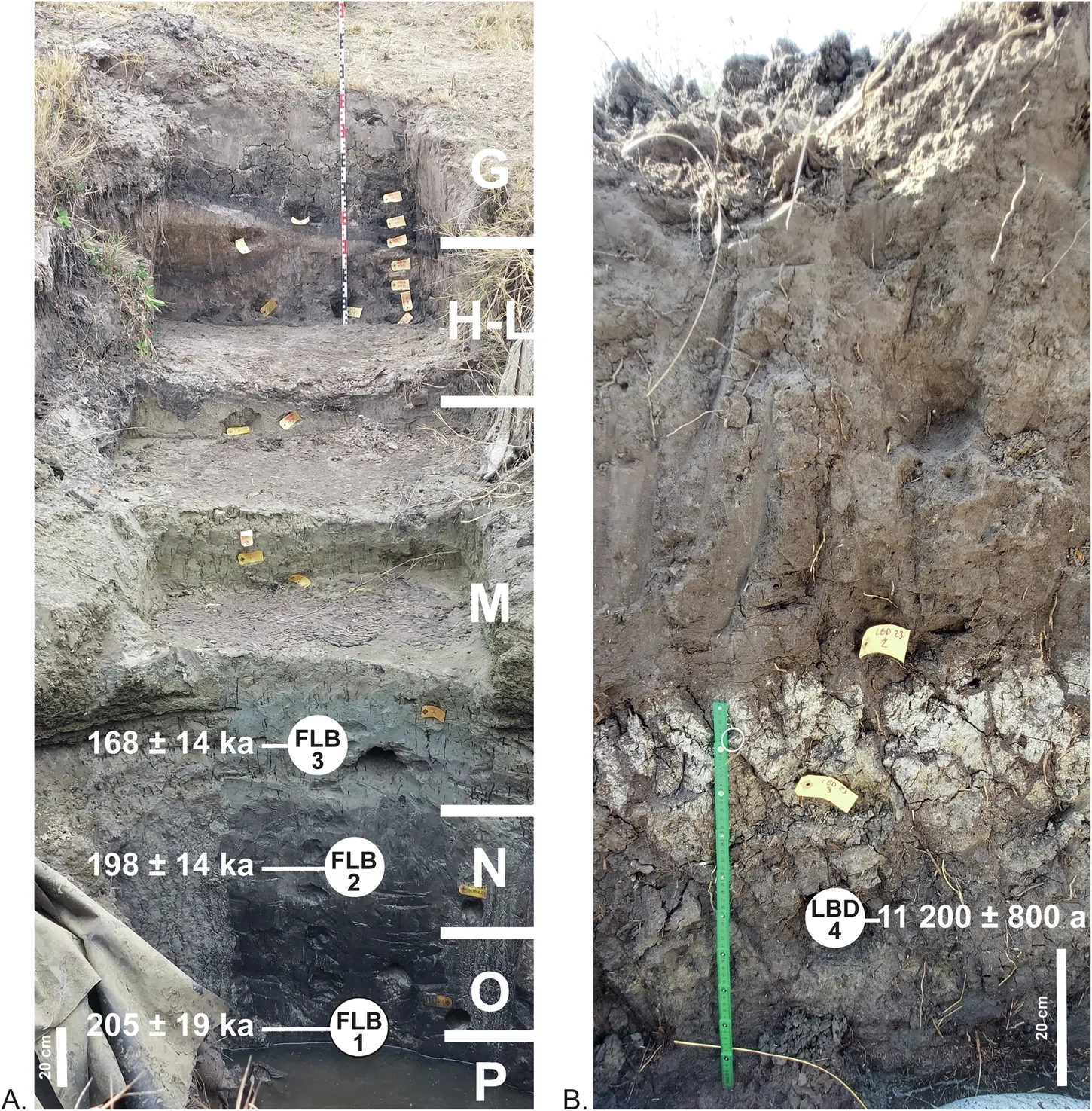
Luminescence dating sampling in the Dreyer Section at Florisbad (A) and at Lombard’s Drift (B). Sample locations and OSL ages are shown. The in situ dosimetry was conducted at the same location as the samples using a portable gamma-ray spectrometer.

Previous interpretations of the palaeoenvironment at Florisbad have linked phases of high lake levels with grasslands and dry phases with an arid landscape, brackish water in smaller pools and wetlands, like those occurring today^23,27^. However, a pollen study showed that this picture of alternating wet and dry phases seems contradictory at some levels, with interpretations requiring different taphonomic possibilities for vegetation reconstruction^28^. The sediment and pollen studies used different sedimentary profiles on which to base their interpretations (Table S1) and the two proxies are characterised by different spatiotemporal scales. In this study, we report plant biomarker stable isotope results from Florisbad’s Dreyer Section covering the Middle Pleistocene to Holocene, and for the nearby Holocene site of Lombard’s Drift (Fig. 1, Figs. S1–S3, SI S1). The results are examined within the framework of newly obtained luminescence ages (Fig. 2). Comparing the palaeoenvironmental reconstructions derived from biomarkers with pollen and faunal records from Florisbad (FLB) and Lombard’s Drift (LBD) we discuss the different environments humans engaged with in the central interior grasslands of South Africa.

## Results

### Plant wax *n*-alkanes

The amount of plant wax *n*-alkanes at FLB varied widely across samples (SI 3). Samples with high *n*-alkane amounts are the exception, and 13 of the 21 samples have less than 500 ng/g sediment *n*-alkane concentration. While some samples show an odd-over-even ratio with a clear odd dominance, other samples do not, suggesting degradation of the *n*-alkane chains in the latter case. This is further reflected in the carbon preference index (CPI) of the C_27_-C_33_ *n*-alkanes, representing terrestrial plant input, which ranges between 0.6 and 7.6 (average 2.8 ± 2.0, *n* = 21) (Table S2). A value higher than 2 is usually accepted as reflecting well-preserved samples^29^. Higher chain lengths dominate, with average chain length (ACL_27-33_) ranging from 29.3 to 32.4 (average 31.0 ± 0.9, *n* = 21) (Table S2). The most abundant *n*-alkane homologue at FLB varies by sample (Fig. S4). In the well-preserved Holocene samples C_31_ is the dominant *n*-alkane while the C_29_ and C_33_ homologues are the second most abundant, indicating a mainly terrestrial plant wax source. In contrast, in the Pleistocene samples, C_33_ is the dominant *n*-alkane with C_31_ the second most abundant *n*-alkane, indicating a strong presence of C_4_ plants and/or grasses. Some samples (UD12 to LD22) have an over-proportionally high dominance of the C_33_ *n*-alkane.

The amount of *n*-alkanes at LBD is higher than at FLB (SI 3, Supplementary Data 1). The carbon preference index (CPI) of the C_27_-C_33_ *n*-alkanes ranges between 1.1 and 2.9 (average 1.9 ± 0.8, *n* = 5) (Table S2). Only the top and bottom samples have CPI values over 2, indicating good preservation. These two samples show an odd-over-even ratio with a clear odd dominance while the other samples have a relatively high abundance of even numbered homologues, suggesting some level of degradation of the *n*-alkane chains in the latter case (Figs. S4 and S5). The dominant *n*-alkane varies by sample (Fig. S5). However, no LBD sample shows the disproportional high C_33_ abundances evident in the FLB Pleistocene samples. Average chain length (ACL_27-33_) at LBD ranges from 29.4 to 30.4 (average 30.0 ± 0.4, *n* = 5), which is lower than at FLB when comparing averages (Table S2).

**Fig. 3:**
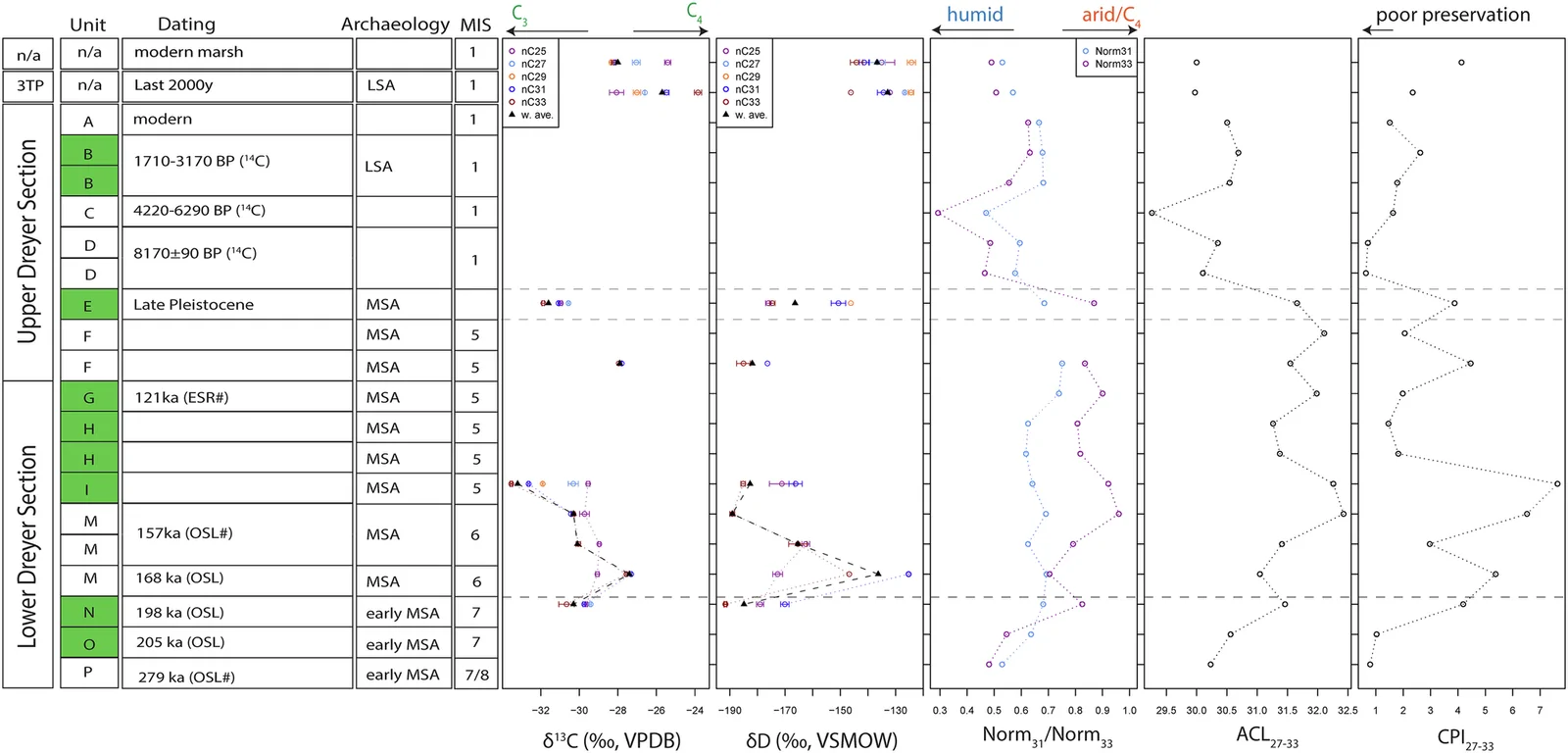
Distribution of biomarkers from Florisbad. From left to right: Sample section, stratigraphic unit after Kuman et al.^23^ with peat layers in green; dating after Scott andNyakale^57^ (^14^C), Grün et al.^19^ (OSL#, ESR#) and new ages (OSL); associated lithic technocomplex; Marine Isotope Stage (MIS); *n*C_25_, *n*C_27_, *n*C_29_, *n*C_31_ and *n*C_33_ δ^13^C mean values and their weighted average; *n*C_25_, *n*C_27_, *n*C_29_, *n*C_31_ and *n*C_33_ δD mean values and their weighted average; *Norm*31 and *Norm*33 index; Average Chain Length (ACL) of the C_27_-C_33_ *n*-alkanes; Carbon Preference Index (CPI) of the C_27_-C_33_ *n*-alkanes. The number of measurements (n) for mean values, error range and weighted average values is between 1 and 4 per sample and is provided in Supplementary Data 1. The 121 ka ESR age represents the teeth found at the contact between G and F and in the bottom 30 cm of F. The hominin cranium was found in a spring vent that cut through Units N-O and P and was sealed by Unit M^19^.

Ten best preserved samples as indicated by reliable CPI values and good odd-over-even-ratio from FLB were sent for stable isotope measurement, and eight could be measured successfully (SI 3). In both carbon and hydrogen isotopes the C_29_ homologue was in too low concentrations for measurement in all but two samples respectively. In contrast, C_33_ could be measured in all samples, reflecting the unusually high concentration of this homologue in the FLB Pleistocene samples (SI 3). The δ^13^C results for the C_25,_ C_27_, C_29_, C_31_ and C_33_ (Table 1 and Supplementary Data 1) of the archaeological samples exhibit comparable isotopic signatures throughout the sequence, with the C_31_ and C_33_ *n*-alkanes displaying similar trends in δ^13^C values throughout (Fig. 3). The δ^13^C values for the Holocene samples 3TP1 and FLB24 are consistently the highest across all homologues (Table 2). However, while 3TP1 ranges from −28.1 ‰ (C_25_) to −23.8 ‰ (C_33_), FLB24 shows a different trend in homologue abundance, ranging from −25.4 ‰ (C_25_) to −28.3 ‰ (C_29_). For the Pleistocene samples (UD12 to LD22), average δ^13^C results are −29.7 ± 0.7 ‰ (C_25_), −30.1 ± 0.6 ‰ (C_27_), −31.9 ‰ (C_29_), −29.83 ± 2.0 ‰ (C_31_) and −30.27 ± 2.1 ‰ (C_33_). The δD results for the C_25,_ C_27_, C_29_, C_31_ and C_33_ (Table 1 and Supplementary Data 1) of the archaeological samples exhibit a wider span of isotopic signatures throughout the sequence, but all homologues still display the same trends (Fig. 3). The Holocene sample 3TP1 shows the highest δD values across all homologues except in C_31_. It ranges from a minimum of −146.2 ‰ (C_33_) to a maximum of −124.4 ‰ (C_29_). The average δD results of the Pleistocene samples (UD12 to LD22) are −172.3 ± 6.2 ‰ (C_25_), −146.2 ‰ (C_29_), −157.7 ± 20.5 ‰ (C_31_) and −176.8 ± 16.0 ‰ (C_33_). No Pleistocene sample had enough C_27_ concentration to be measured for hydrogen stable isotopes.

**Table 1:** Stable isotope results for the Florisbad sediment samples

Sample	Section/ Depth	δ^13^C *n*C_25_	δ^13^C *n*C_27_	δ^13^C *n*C_29_	δ^13^C *n*C_31_	δ^13^C *n*C_33_	δD *n*C_25_	δD *n*C_27_	δD *n*C_29_	δD *n*C_31_	δD *n*C_33_
FLB-24	Modern marsh	−25.41 ± 0.2 (3)	−27.04 ± 0.2 (3)	−28.34 ± 0.1 (3)	−28.16 ± 0.0 (3)	−28.21 ± 0.1 (3)	−135.1 ± 4.7 (2)	−136.6 ± 2.8 (2)	−124.3 ± 1.5 (2)	−141.3 ± 1.8 (2)	−144.2 ± 2.2 (2)
FLB-3TP1	3rd test pit	−28.07 ± 0.4 (3)	−26.60 ± 0.0 (3)	−27.01 ± 0.2 (3)	−25.47 ± 0.1 (3)	−23.84 ± 0.2 (3)	−132.1 (1)	−126.7 ± 0.3 (3)	−124.4 ± 1.0 (2)	−134.4 ± 2.2 (3)	−146.2 ± 0.0 (2)
FLB-UD12	Upper Dreyer section	−30.96 ± 0.1 (3)	−30.56 ± 0.0 (2)		−31.08 ± 0.0 (3)	−31.87 ± 0.1 (3)	−176.1 ± 1.0 (3)		−146.2 (1)	−150.7 ± 2.6 (3)	−174.6 ± 1.0 (3)
FLB-UD14	Upper Dreyer section				−27.80 ± 0.0 (2)	−27.95 ± 0.0 (2)				−176.4 (1)	−185.1 ± 2.5 (2)
FLB-LD18	Lower Dreyer section	−29.54 ± 0.1 (3)	−30.31 ± 0.3 (3)	−31.90 ± 0.0 (2)	−32.63 ± 0.1 (3)	−33.53 ± 0.1 (3)	−171.2 ± 4.5 (4)			−166.2 ± 2.4 (4)	−185.2 ± 0.8 (4)
FLB-LD19	Lower Dreyer section	−29.73 ± 0.3 (3)			−30.43 ± 0.0 (3)	−30.30 ± 0.1 (3)					−189.1 ± 0.9 (4)
FLB-LD20	Lower Dreyer section	−28.97 ± 0.1 (3)				−30.05 ± 0.1 (3)	−162.5 ± 1.4 (3)				−165.4 ± 3.3 (2)
FLB-LD21	Lower Dreyer section	−29.06 ± 0.1 (3)			−27.31 ± 0.0 (3)	−27.56 ± 0.1 (3)	−172.7 ± 1.8 (3)			−125.3 (1)	−146.8 (1)
FLB-LD22	Lower Dreyer section	−29.64 ± 0.1 (3)	−29.41 ± 0.1 (2)		−29.75 ± 0.1 (3)	−30.66 ± 0.4 (3)	−179.0 ± 1.3 (3)			−170.1 ± 1.5 (3)	−191.6 ± 0.5 (3)

All results are given as the average value of all measurements per sample in per mill (‰), with the δ^13^C values given against VPBD, δD values are given against VSMOW. The standard deviation (STDEV) is provided and the number of measurement is shown in brackets.

**Table 2:** Stable isotope results for the Lombard’s Drift sediment samples

Sample	Section/ Depth	δ^13^C *n*C_25_	δ^13^C *n*C_27_	δ^13^C *n*C_29_	δ^13^C *n*C_31_	δ^13^C *n*C_33_	δD *n*C_25_	δD *n*C_27_	δD *n*C_29_	δD *n*C_31_	δD *n*C_33_
LBD1	0.4 m	−25.09 ± 0.5 (3)	−24.99 ± 0.1 (3)	−28.02 ± 0.3 (3)	−28.56 ± 0.1 (3)	−26.29 ± 0.1 (3)	–113.6 ± 3.2 (4)	−143.3 ± 1.7 (4)	−122.4 ± 2.1 (4)	−139.7 ± 1.3 (4)	−124.1 ± 1.4 (4)
LBD2	0.6 m	−28.50 (1)	−27.77 ± 0.1 (3)	−29.09 ± 0.1 (3)	−28.98 ± 0.0 (3)	−27.51 ± 0.2 (3)	−157.7 ± 7.2 (4)	−138 ± 5.0 (4)	−131.2 ± 5.0 (4)	−124.7 ± 6.8 (4)	−119.4 (1)
LBD3	0.8 m				−31.05 ± 0.2 (3)	−30.42 ± 0.6 (3)					
LBD4	1.1 m		−30.31 ± 0.3 (2)	−31.00 ± 0.1 (2)	−31.12 ± 0.2 (2)	−29.37 ± 0.2 (2)	−126.1 (1)	−142.0 ± 4.4 (4)	−133.3 ± 9.4 (4)	−136.7 ± 8.4 (4)	
LBD5	1.3 m		−29.26 ± 0.0 (3)	−30.35 ± 0.2 (3)	−31.29 ± 0.1 (3)	−30.36 ± 0.1 (3)	−99.7 ± 0.5 (4)			−130.2 ± 1.3 (4)	−107.7 ± 2.8 (4)

All results are given as the average value of all measurements per sample in per mill (‰), with the δ^13^C values given against VPBD, δD values are given against VSMOW. The standard deviation (STDEV) is provided and the number of measurement is shown in brackets.

**Fig. 4:**
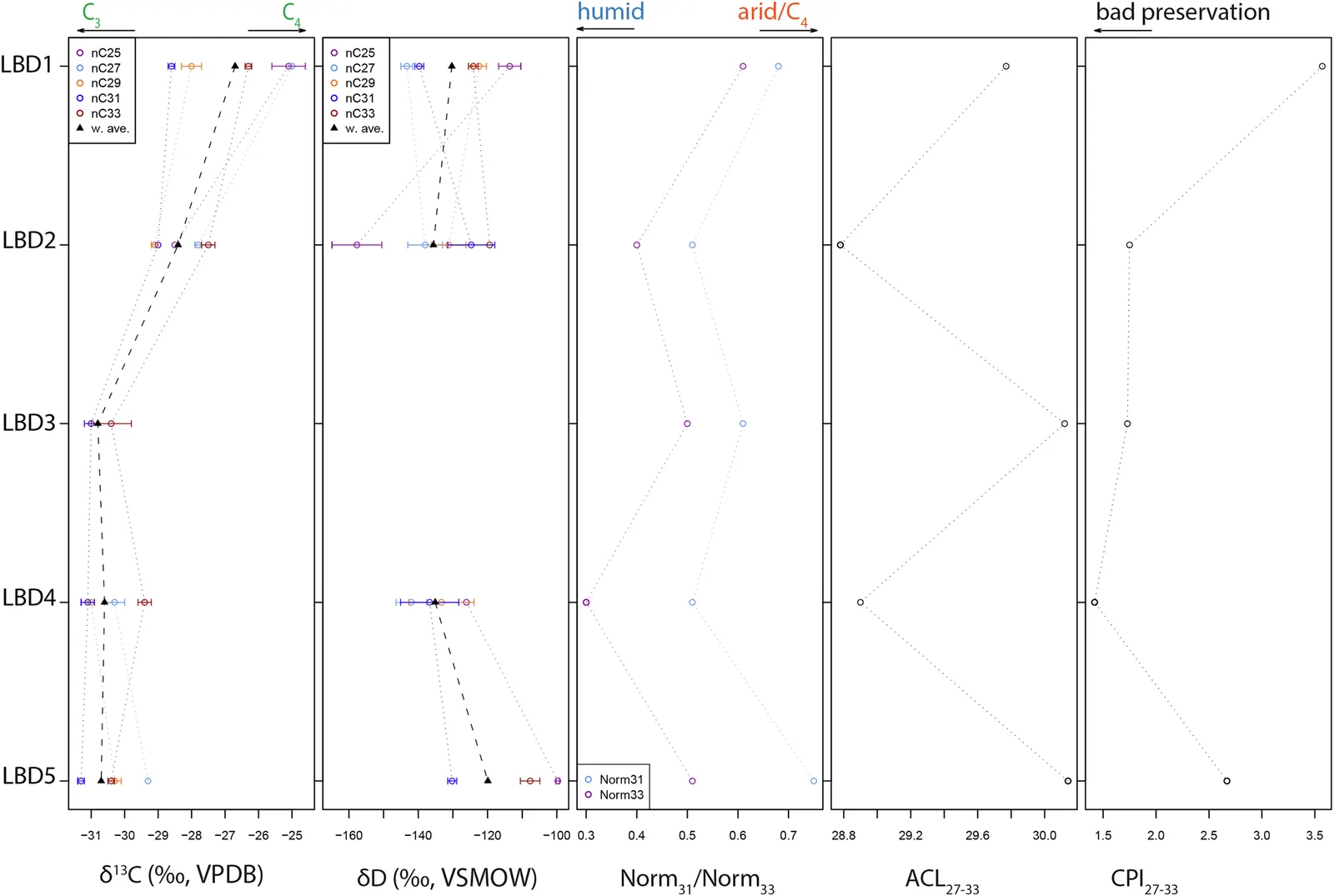
Leaf wax *n*-alkane results for Lombard’s Drift. From left to right: Sample name; δ^13^C isotope values and their weighted average; δD isotope values and their weighted average; *Norm*31 and *Norm*33 index; Average chain length (ACL) of the C_27_-C_33_ *n*-alkanes; Carbon Preference Index (CPI) of the C_27_-C_33_ *n*-alkanes. The number of measurements (n) for mean values, error range and weighted average values is between 1 and 4 per sample and is provided in Supplementary Data 1.

All five LBD samples were measured for stable isotopes (Table 2 and Fig. 4). Average δ^13^C results are −26.8 ± 2.4 ‰ (C_25_), −28.1 ± 2.3 ‰ (C_27_), −29.6 ± 1.3 ‰ (C_29_), −30.2 ± 1.3 ‰ (C_31_), and −28.8 ± 1.8 ‰ (C_33_) and therefore higher than the FLB Pleistocene averages, but lower than the very recent FLB samples. Average δD results at LBD are −124.3 ± 24.8 ‰ (C_25_), −141.1 ± 2.8 ‰ (C_27_), −129.0 ± 5.8‰ (C_29_), −132.8 ± 6.7 ‰ (C_31_), and −117.1 ± 8.4 ‰ (C_33_), on average lower than the FLB samples (Table 2).

### Magnetic properties

The magnetic susceptibility (MS) and anhysteretic remanent magnetisation (ARM) values at FLB show large shifts that correspond to transitions between organic-rich (peat) and sand layers (Figs. S6 and S7). In addition, results revealed clear shifts within Unit M (sample 39) and Unit B (sample 4), invisible to the naked eye, which may be associated with breaks in the sedimentation, erosional hiatuses, or changes in sediment source (Figs. S6–S8). In places, the ARM shifts are larger than the MS shifts, reflecting changes in the concentration of magnetite grains, a common component of local sands derived from the breakdown of dolerite^27^. The MS results of LBD show a clear shift between the values of the peat and pale-green sandy clay and the overlying organic-rich sediment, probably indicating the existence of an unconformity or break in the sedimentation (Figs. S9 and S10).

### Luminescence dating

In the Dreyer Section at FLB, quartz and feldspars from three sediment samples collected from Unit O (early MSA, FLB1), Unit N (early MSA, FLB2) and Unit M (MSA, FLB3) were dated using optically stimulated luminescence (OSL), thermally transferred-optically stimulated luminescence (TT-OSL), and post-infrared infrared stimulated luminescence (pIRIR_225_ and pIRIR_290_) (Fig. 2). The sample from Lombard’s Drift (LBD4) was dated using OSL. Measurements were conducted on different grain size fractions depending on the nature of the samples (4-11 µm for FLB1 and FLB2, 100-140 µm for FLB3, and 100-120 for LBD4). The measurement protocols were applied either on multigrain (MG) aliquots, on single-grain (SG, one grain per hole) or micro-aliquots (MA, two to three grains per hole) (see details in Table 3 and in the Methods section). The equivalent doses (D_e_) were computed using the Central Age Model^30^ (CAM, Table 3).

**Table 3:** Luminescence ages (1 σ) obtained for Florisbad (FLB) and Lombard’s Drift (LBD)

Sample	Depth (m) & Unit	Protocol	*n*	OD (%)	D_e_ (Gy)	Dose rate (µGy/a)					Age (ka)
						Alpha	Beta	Gamma	Cosmic	Total	
FLB1	6.4 (O)	OSL (MG)	11	<1	366 ± 6	159 ± 23	965 ± 11	566 ± 14	92 ± 10	1782 ± 30	205 ± 19
		TT-OSL (MG)	14	4 ± 2	496 ± 9	159 ± 23	965 ± 11	566 ± 14	92 ± 10	1782 ± 30	278 ± 26
		pIRIR_225_ (MG/faded)	10	<1	489 ± 8	362 ± 64	986 ± 11	566 ± 14	92 ± 10	2005 ± 68	244 ± 26
		pIRIR_225_ (MG/corrected)									251 ± 26
		pIRIR_290_ (MG)	15	9 ± 4	408 ± 16	362 ± 64	986 ± 11	566 ± 14	92 ± 10	2005 ± 68	204 ± 23
FLB2	5.9 (N)	OSL (MG)	11	<1	303 ± 5	113 ± 16	788 ± 7	530 ± 13	97 ± 10	1527 ± 24	198 ± 14
		TT-OSL (MG)	10	<1	394 ± 7	113 ± 16	788 ± 7	530 ± 13	97 ± 10	1527 ± 24	258 ± 18
		pIRIR_225_ (MG/faded)	10	<1	365 ± 5	257 ± 46	807 ± 7	530 ± 13	97 ± 10	1691 ± 49	216 ± 18
		pIRIR_225_ (MG/corrected)									233 ± 21
		pIRIR_290_ (MG)	14	8 ± 5	426 ± 17	257 ± 46	807 ± 7	530 ± 13	97 ± 10	1691 ± 49	252 ± 22
FLB3	5.5 (M)	SG OSL (SG)^a^	58	29 ± 3	214 ± 9	21 ± 3	722 ± 11	433 ± 10	101 ± 10	1277 ± 18	168 ± 14
		TT-OSL (MA)	214	28 ± 2	383 ± 10	21 ± 3	722 ± 11	433 ± 10	101 ± 10	1277 ± 18	300 ± 24
		pIRIR_225_ (MG/faded)	15	6 ± 2	327 ± 6	47 ± 8	1106 ± 48	433 ± 10	101 ± 10	1686 ± 51	194 ± 14
		pIRIR_225_ (MG/corrected)									245 ± 21
		pIRIR_290_ (MG)	24	7 ± 1	356 ± 6	47 ± 8	1106 ± 48	433 ± 10	101 ± 10	1686 ± 51	211 ± 15
LBD4	1.5	OSL (MG)	23	9 ± 2	27 ± 1	43 ± 6	1356 ± 12	833 ± 20	170 ± 10	2401 ± 25	11.2 ± 0.8

The equivalent doses (D_e_) were measured on fine grains (4–11 µm) for FLB1 and FLB2, on 100–140 µm grains for FLB3 and on 100–120 µm grains for LBD4. For multigrain (MG) analyses, single-grain (SG) and micro-aliquots (MA) analyses, the D_e_ were computed with the Central Age Model (CAM)^30^ from the number of accepted grains (*n*, for SG and MA: 1500 measured grain positions measured). Alpha and beta dose rates were calculated from the radioactive elements measured by gamma spectrometry (U, Th, and K content, see Table S4) using the conversion factors of Guérin et al.^110^. The gamma dose rate was measured in situ using a portable gamma spectrometer. Cosmic dose rates were estimated from current burial depths below the surface using the equation from Prescott and Hutton^109^. The ages were calculated taking into account beta absorption factors of Guérin et al.^111^ and alpha attenuation factors of Brennan et al.^112^; an s-a value of 4.5 ± 20 % for the quartz and an a-value of 0.08 ± 25 % for the feldspars. The K-content in the feldspars measured using SEM-EDS is11.4 ± 1.4 %. ^a^The values < 12 Gy were considered as contamination and deleted from the dataset (*n* = 4). Note that for MG aliquots, the fine grains of FLB1 and 2 are covering the whole disks while for FLB3 and LBD4, the aliquot size is 1 mm Ø. For the SG OSL analyses of FLB3, one grain per hole was measured in the 150 µm-hole disks; for the MA TT-OSL analyses of FLB3, 2-3 grains per hole were measured in 300 µm-hole disks in order to increase the yield.

The OSL ages obtained for Florisbad are 205 ± 19 ka, 198 ± 14 ka, and 168 ± 14 ka for FLB1, FLB2, and FLB3, respectively, and 11.2 ± 0.8 ka for Lombard’s Drift (LBD4). Representative dose response curves (DRC) obtained for each sample are provided in Fig. S11. Considering that the OSL signal of FLB3 was close to saturation (Fig. S11), single grain measurements were conducted on this sample. The statistics regarding the single grain selection criteria are presented in Table S3. The single grain data of FLB3 was treated considering the effect of D_0_ on the D_e_. Quartz grains were rejected at progressively higher D_0_ values^31,32^ the D_0_ threshold (i.e., the minimum value to avoid saturated grains) is 150 Gy, for which the number of saturated grains reached 0 (Fig. S12). We selected D_e_ with D_0_ ≥ 150 Gy to calculate the CAM D_e_ for FLB3, resulting in a CAM D_e_ of 214 ± 9 Gy and an overdispersion value of 29 ± 3 %.

The OSL age of FLB1 agrees with the pIRIR_290_ age of 204 ± 23 ka. For FLB2 and 3, the pIRIR_290_ ages of 252 ± 22 ka and 211 ± 15 ka are older than the OSL ages (Fig. S13). The pIRIR_225_ faded ages obtained on multigrain aliquots are in agreement with the quartz OSL ages for FLB1 (244 ± 26 ka), FLB2 (216 ± 18 ka), and FLB3 (194 ± 14 ka). Moreover, these ages may suffer from fading and thus need to be corrected^33–36^. Fading rates measured differ from one sample to the other: 0.31 ± 0.16 (FLB1), 0.78 ± 0.21 (FLB2) and 2.27 ± 0.32 (FLB3) (Fig. S14), presumably due to the nature of the grain analyses (polymineral versus potassium feldspars). Corrected ages are 251 ± 26 ka (FLB1), 233 ± 21 ka (FLB2), and 245 ± 21 ka (FLB3). Taking into account the error at 1 σ, the ages obtained using the pIRIR protocols fall within the same range. However, considering the difficulties related to the estimation of the fading rates, which includes large error ranges, we must consider the pIRIR_225_ corrected ages with caution. The TT-OSL ages for all samples also appear to be overestimated. It is known that the pIRIR_290_ and TT-OSL signals do not bleach as fast as the OSL signal [e.g., refs. ^37,38^], and that small residual doses are expected for the pIRIR_225_ signal^35,39^. Due to the slower optical resetting rate of these signals compared to the conventional OSL signal of quartz, we cannot exclude that a residual dose may remain. Nevertheless, the pIRIR_225_ uncorrected ages and the OSL ages overlap; the effects of the residual dose and of the fading may thus be compensated for the pIRIR_225_ signal.

The dose recovery tests (DRT, recovered/given dose) were conducted for each sample and each protocol (Fig. S15). They range from 0.96 ± 0.03 (LBD4) to 1.00 ± 0.02 (FLB2) for the MG OSL protocol. For the SG measurements of FLB3, we sorted the accepted grains according to their D_0_ values and recalculated the recovered/given dose ratio while increasing the D_0_ threshold (Fig. S16). A plateau is reached from D_0_ ≥ 140 Gy (for which the number of saturated grains in the dataset reaches 0), producing a value of 1.03 ± 0.16 for the DRT. The DRT are underestimated for the TT-OSL protocol on multigrain for FLB1 and FLB2 (0.80 ± 0.07), and is 1.52 ± 0.54 for the TT-OSL protocol on micro-aliquot for FLB3. However, for the pIRIR_225_ protocol, the ratios obtained on feldspars range from 1.03 ± 0.08 (FLB3) to 1.15 ± 0.05 (FLB1), and for the pIRIR_290_ protocol, they range from 0.94 ± 0.06 (FLB3) to 1.1 ± 0.24 (FLB2). Overdispersion (OD) values suggest that the samples are homogeneous, with OD values obtained on MG < 9 % (Figs. S17–S19). The SG dataset produced higher OD values (29 %), consistent with OD values measured in well-bleached samples^40^. Considering that the DRT ratios were not satisfactory for the TT-OSL analyses, and that incomplete bleaching of both TT-OSL and pIRIR_290_ signals is suspected, we therefore give more credibility to the ages obtained using conventional OSL on quartz.

The OSL age of LBD4 is 11200 ± 800 years, falling at the very beginning of the Holocene. The DRT value of 0.96 ± 0.03 is satisfactory (Fig. S15), as well as the OD value of 9 % (Fig. S20), which suggests that the grain population is homogeneous and that the OSL signal of the quartz has been fully bleached before deposition.

## Discussion

**Fig. 5:**
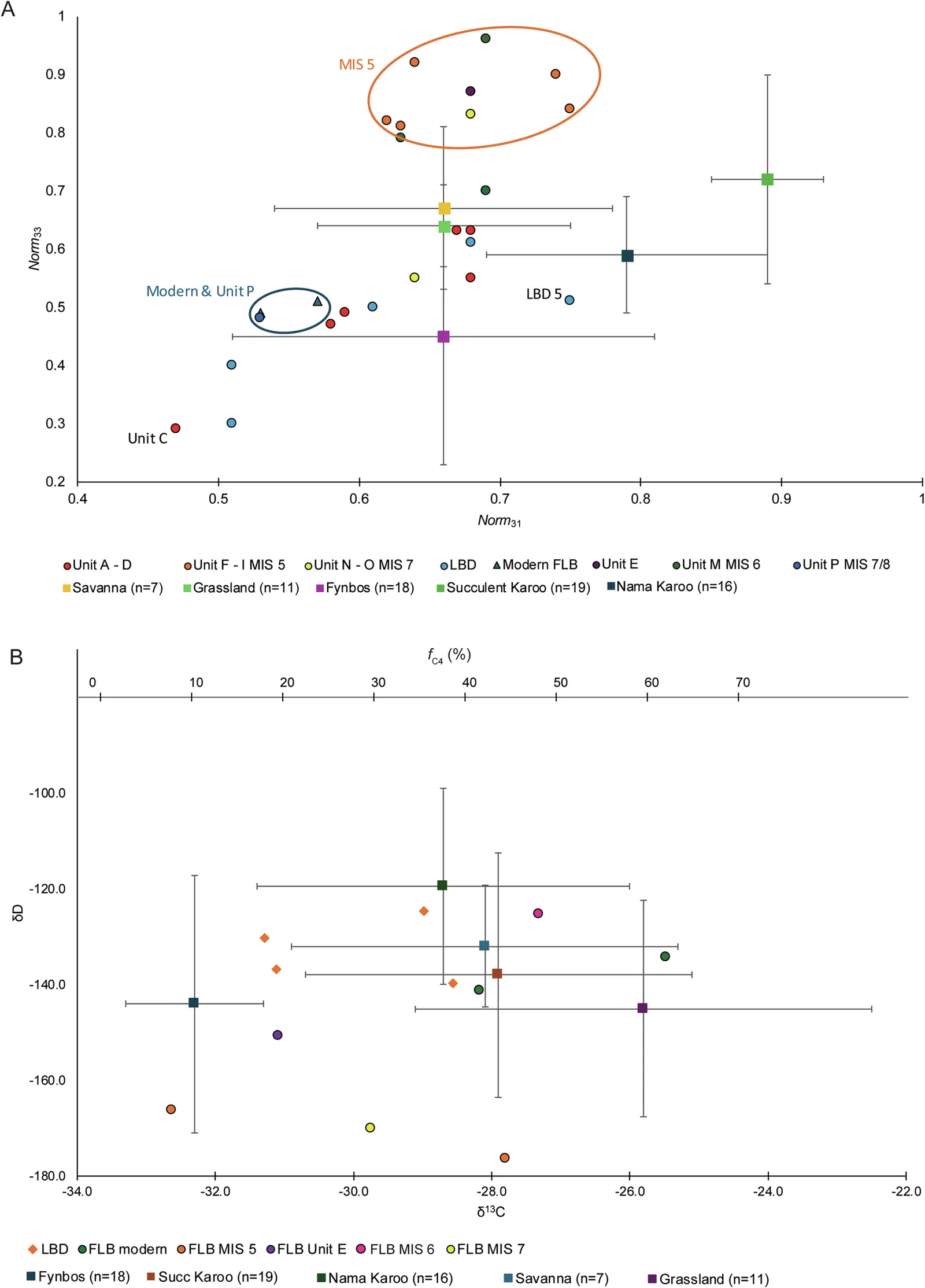
Comparison of the *n*-alkane indices and stable isotope results to modern biomes in South Africa, showing that particularly the MIS 5 and MIS 6 samples do not fall within modern biomes. **A** Scatterplot of the *Norm*31 and *Norm*33 indices for the Florisbad and LBD samples as well as modern biomes in South Africa^41^. **B** Scatterplot of the C_31_ δ^13^C (‰ VPDB) and δD (‰ VSMOW) values for the Florisbad and LBD samples as well as modern biomes in South Africa^41^. The fraction of C_4_ vegetation, *f*_C4_^100^, for C_31_ calculated from modern African vegetation endmembers^101^ is plotted on the top x axis. All error bars are one standard derivation, the centre of the error bar is the average value.

**Fig. 6:**
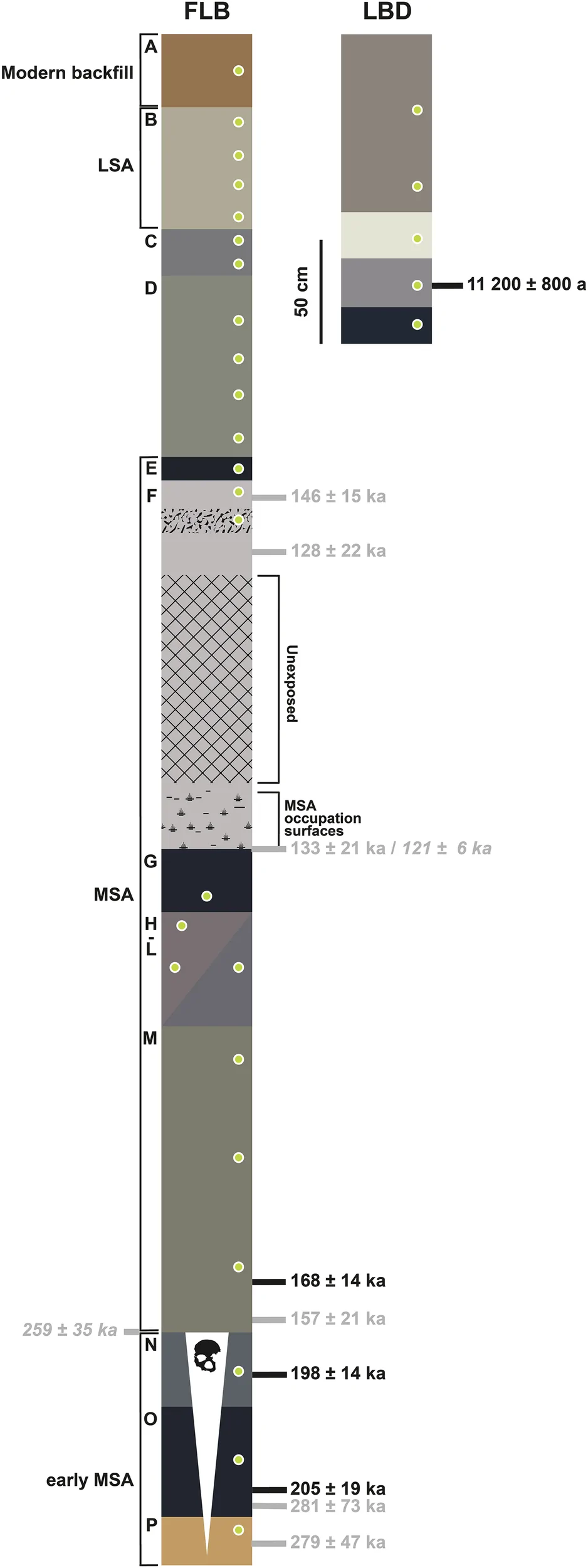
Stratigraphic column synthesising the Dreyer Section and Lombard’s Drift sections and the location of the OSL (regular) and ESR (italic) samples, as well as the plant biomarker samples (yellow dots). Note that the ages from Grün et al.^19^ appear in grey while our OSL ages are in black and that the ages of Unit F were obtained from Test Pit 3 and are shown here to provide a chronological reference to the Late Pleistocene sedimentary contexts. The human fossil was found at the bottom of the spring vent that cut through Units P to N and was sealed by Unit M; in this figure, the age was placed at the transition between Units N and M to indicate that it is stratigraphically younger than Unit N and older than Unit M.

At both sites the youngest samples (FLB24, 3TP1, LBD1) are closest to the modern Grassland Biome stable isotope values^41^, while both δ^13^C and δD values at LBD are on average higher than at FLB (Fig. 5). FLB is calculated to have between 41 % (FLB24) and 61 % (3TP1) C_4_ plant cover, which in the study area mostly represents graminoids adapted to summer rain while at LBD the uppermost sample has the highest percentage of C_4_ plant cover (38 %). The different site settings are a potential factor for this difference as LBD currently has a high amount of *Phragmites australis*, a C_3_ Cyperaceae, growing at the sampling location. The δD values at both sites are within the range of the weighted δD mean values for the Grassland Biome of modern samples^42^. However, the *Norm*31 and *Norm*33 indices are within the modern Fynbos Biome range, reflecting that the local saline spring and wetland support a specific vegetation community even though it is likely that the local communities would only have included few fynbos species, likely some from the Ericaceae, Restionaceae, Asteraceae and Thymelaeaceae for limited periods^28^. In contrast, most Pleistocene samples are not within this isotopic range, except for lower Unit M. The *Norm*31 and *Norm*33 indices all indicate a very different range for the Late Pleistocene (Unit E), MIS 5 (Unit F to I) and MIS 6 (Unit M), compared to the Holocene and modern biome ranges (Fig. 6A). The lower Unit M and Unit N samples are the most heterogenous suggesting different environmental conditions within this stage (Fig. 5). An exception is the lowermost Unit P (MIS 7/8), which falls within the range of the modern sample from the Florisbad wetland (Fig. 5A). All together, these results show non-analogue environments for most parts of the Pleistocene that must be a result of a different vegetation and/or rainfall structure than today. Modern studies showed that specifically for the Summer Rainfall Zone (SRZ) in South Africa, δD values from *n*-alkanes correlated with annual rainfall amounts^42^. However, due to the high evapotranspiration in this area, the range of δD is extended from the pure rainfall range values^42,43^. Another factor is the source of the rainfall and the distance from the site to the source^44^, with FLB and LBD located in an area where most moisture comes mainly from evaporation over the southern African landmass and the Indian Ocean, particularly the Mozambique Channel^45^. Today, moisture from the South Atlantic Ocean is negligible, but might have had an influence in the past when westerlies shifted northwards^46–48^. Further potential influences on δD values are differences in plant physiology, such as between dicots and monocots^49,50^, photosynthetic pathways^51–53^, and ecosystem evapotranspiration^43,54,55^.

The lowermost Unit P at FLB is an aeolian sand dune reworked by spring water, altered by pedogenesis and bioturbation. In contrast, overlying Unit O is peat with variable amounts of quartz silt and sand that marks a complete change in sedimentary environment, as indicated by the large shift in MS and ARM. Unit P was dated to 279 ± 47 ka^23^, while according to the description of the original excavator^19^, the spring vent with fossil remains cut through Peat I (Unit O-N) and was sealed by the Green Sand (Unit M), which means that the human remains would be younger than Peat I, as discussed in detail below. Pollen counts for Unit P include some fynbos species typical in certain interior upland areas outside the well-known Cape Fynbos Biome^28,56^ and indicate cool conditions. Further hints at fynbos-type contributions are the *Norm*33 index values for sample FLB-LD24, which fall within the range of modern Fynbos Biome *Norm*33 values (Fig. 5). In general, biomarker indices for Unit P and O are substantially lower than for the rest of the Pleistocene FLB sequence, but poor preservation prevented stable isotope measurements in Units O and P.

Unit N is a transitional layer between the peat of Unit O (of which it retains a large quantity of decayed plant material) and the green sand of Unit M. The new OSL ages are 198 ± 14 ka for Unit N and 205 ± 19 ka for Unit O. The overlapping range of the OSL ages indicates that these sediments accumulated around 200 ka. Fynbos pollen (Ericaceae and Restionaceae) have been found in Unit O and N, and the calculated percentage of C_4_ plants in Unit N is 30 % (Fig. 5B). Across all our samples, δ^13^C values indicate a C_3_ and C_4_ mixed vegetation. This suggests that at least the lower units around Unit O during Middle Pleistocene occupation at Florisbad have, in addition to C_4_ grasses, a substantial C_3_ plant component. This probably includes local spring elements as well as fynbos components and karroid vegetation associated with cooler climates. Vegetation and cooler climates tempered evapotranspiration resulting in more even seasonal regimes, taking into account that winter rain did not penetrate as far into the interior as the study area^47^. In contrast, karroid vegetation with limited C_4_ grass cover, which is prominent in the early Holocene layers, is probably the result of precession-related dry seasonality^57,58^.

Unit M is a deposit of quartz sand embedded in glauconite clay that accumulated in a shallow lake, probably fed by the spring^27^. This unit includes MIS 6, based on the OSL age of 157 ± 21 ka^19^ for the upper part of Unit M. The lower part of Unit M is dated in this study to 168 ± 14 ka (OSL). The lower δ^13^C and δD values in the upper part of Unit M, and the very high values in the lower part, could at first glance represent the glacial/interglacial divide between MIS 6 and MIS 7, further indicated by a shift in MS and ARM values. However, our biomarker isotope values generally do not show a glacial/interglacial pattern as might be expected^59^. A shallow lake is indicated by the presence of Cyperaceae and rare aquatics like *Typha*. The bedding is similar to that of underwater sedimentation in lower parts of Unit M, and glauconite implies brackish water, which is supported by the presence of Amaranthaceae pollen indicating seasonal evaporation. The prominence of these halophytes is apparently in conflict with indications of lake conditions but strong evaporative conditions may have caused these plants to spread along the lake edges or in dried out ponds. While we expect more grasses and Cyperaceae during humid phases, the low numbers of these, especially in van Zinderen Bakker’s profile in the Main Excavation, may be the result of relative (%) over-representation or selective preservation of pollen in mud cracks^27,28,60,61^. Lower Unit M has the highest δD value of the Pleistocene samples (Fig. 5B), comparable to those of today, indicating a strong summer rainfall regime, and a calculated C_4_ plant cover of 47 %, in line with today’s vegetation. This could suggest interglacial climatic conditions with strong evaporation and associated vegetation comparable to today. However, ACL_27-33_ and *Norm*31 indicate wetter conditions in lower Unit M and more aridity in upper Unit M. Additional drivers, such as precessional signals, could influence the local precipitation signal, which played a prominent role in the interior of Southern Africa^62^.

Upper Unit M (MIS 6) indicates a less intense summer rainfall, or potential winter rainfall influence, a reduction in C_4_ plant cover (25 %) and δD values comparable to Unit N. *Norm*31 and *Norm*33 indices in upper Unit M fall outside the range of modern biomes (Fig. 5A). There is further evidence for more standing water in the environment than today in this timeframe from the wider region. Stratum 2 in Excavation 2 at Wonderwerk Cave, with an early MSA lithic assemblage, is dated to ~240–150 ka^15^. A wetter-than-present phase has been suggested for this time frame based on micromorphological, faunal, and stable isotope studies. The faunal isotopes also show lower δ^13^C values in the enamel of grazers than comparable species today indicating more C_3_ plant presence, consisting of various plant types like woody species or local spring elements including C_3_ grasses such as *Phragmites*^15^. Additionally, in the Nama Karoo Biome, the Bundu Farm faunal assemblage and ostrich eggshell isotopes have been interpreted as representing a wetter phase in the Upper Karoo sometime between MIS 7–8^63,64^.

The Last Interglacial (MIS 5; Unit F to L) environment was a time of oscillating conditions dated to 146 ± 15 ka (OSL) and 121 ± 6 ka (electron spin resonance, ESR)^19,26^. This is reflected by the rapid accumulation of thin sedimentary units that alternate peat (Unit K, Unit G), organic-rich sand (Unit I, Unit L), and white sand (Unit J, Unit H, Unit F)^65^, as indicated also by MS and ARM shifts. The well-preserved biomarkers show δ^13^C values are lower than in upper Unit M, indicating less C_4_ plant influence. In contrast, pollen indicate grassy conditions. The Unit I sample (FLB-LD18) has the lowest measured δ^13^C value of the study (below -30 ‰), with the weighted average of this sample in the range of values for the modern Fynbos Biome^41^ and the lowest calculated C_4_ plant cover with 8 % (Fig. 5B). However, rather than elements of fynbos or karroid vegetation, which is not supported by pollen, the difference could be due to biomarkers being mainly influenced by C_3_ or CAM plants that produce higher *n*-alkane concentrations than grasses (SI 3), as this part of the sequence has the sediment samples with the highest C_33_ homologue concentrations, which drive the low δ^13^C values. High pollen concentrations of the succulent CAM plant group Aizoaceae have been found between Peat I and Peat II^28^. Today, these plants spread when pans in the areas are drying up. ACL, *Norm*31 and *Norm*33 indices indicate relatively dry conditions in MIS 5, which is most likely driven by reflecting these succulents in our biomarker samples. Another possibility for the high C_33_ homologue concentrations in combination with low δ^13^C isotope values is the presence of C_3_ grasses^66^ (SI 3). However, very little is known about the C_3_ grass *n*-alkane distributions and chemotaxonomy in South Africa^67^. *Norm*31 and *Norm*33 indices are substantially higher than in modern biomes (Fig. 5A), indicating another phase of environmental conditions with no modern analogues within South Africa.

Unit G is dated to 121 ± 6 ka by ESR from dating mammal teeth found at the contact between G and F, as well as within the first 30 cm of F^19^. This is also where the main MSA find horizon is located^65^. The pollen record and the biomarker indices show fluctuating, sometimes marshy or moist, and occasional dry or cool, generally grassy conditions. However, biomarkers are in general poorly preserved in Unit G. Sediments from Unit F, made of white sand, reworked by water, indicate beach-like deposits, while pollen indicates a wetland environment. Cool phases are confirmed by the presence of fynbos pollen such as Ericaceae, *Passerina* (Thymelaeaceae), and Restionaceae^28,60^. Lower Unit F sample is a lens of high organic content with good biomarker preservation that must have accumulated quickly. The calculated percentage of C_4_ plants in the landscape is 44 %, in the range of the modern samples. The fauna in Unit F is dominated by lechwe (*Kobus leche*) and blesbok (*Damaliscus pygargus*), with no browsers or other mixed-feeders such as eland (*Taurotragus oryx*) and modern springbok (*Antidorcas marsupialis*)^23,25,68^, potentially reflecting the increased C_4_ grass cover as well as standing water during this time.

The uppermost Pleistocene deposits at FLB are undated but are assumed to fall within MIS 4 (Unit E), associated with a few later MSA artefacts, significantly weathered. Sediments here show land exposure, and rapid accumulation related to Peat III formation with a karroid pollen assemblage with Asteraceae. This matches the biomarker indices that show relatively arid conditions in δD isotopes and ACL and *Norm* indices, but a low δ^13^C value indicates a low C_4_ plant influence (20 %).

Biomarker indices and isotopic results show stark contrasts between the Holocene and Pleistocene samples. This highlights that the Holocene is not the same as previous interglacials. Kathu Pan is one of the few comparable sites in the region spanning MIS 6 to 1. A different rainfall seasonality was inferred for Kathu Pan for MIS 6-3, in contrast to MIS 2–1 based on sediment mineral content^69^. This contrast might be reflected in the stark difference of our biomarker indices between MIS 7–4 and the Holocene (MIS 1). Although we have limited time control for the Holocene at LBD, except for the second lowermost sample (LBD4; 1.1 m depth; OSL age 11.2 ± 0.8 ka), we use the *n*-alkane isotopic data from LBD in combination with FLB to investigate variation during the Holocene in a local setting. The *Norm*31, *Norm*33 and ACL_27-33_ indices at LBD alternate between values that are close to the modern Grassland as well as Fynbos Biomes^41^ and lower values, potentially indicating Nama Karoo influence (Fig. 5). The highest δD values at LBD are at 11.2 ± 0.8 ka, showing again that C_4_ grasses and hydrology are not necessarily linked. Cyperaceae and high amounts of *Typha* pollen indicate potentially standing water, whereas high charcoal concentrations in the uppermost unit are caused by periodic fires (SI 2, Table S11).

The oldest Holocene Unit at FLB is Unit D, dated to 8170 ± 90 BP (uncalibrated) by ^14^C dating^57^. Sediments and pollen indicate lacustrine/lake bottom deposits. Our biomarker indices indicate a slightly more humid environment than today, which corresponds to other regional records that indicate a more humid early part of the Holocene in the region^70–73^. Unit C could be even more humid, with the lowest *Norm*31 and ACL_27-33_ values in the whole sequence. However, this contrasts with pollen indicating dry land by a high amount of Amaranthaceae, and sediments that show a lake retreat and peat formation. However, a high Amaranthaceae pollen presence may not always conflict with humid conditions when accompanied by strong evaporation that results in seasonal drying and exposed pan floors that support these halophytes. The biomarker findings, especially concerning the *Norm*31 and *Norm*33 indices (Fig. 5A) could also represent a narrow layer identified in the pollen study with an extremely high peak in Asteraceae (almost 80 %) between 4500 and 5500 BP^57^. Additionally, high Asteraceae counts in ocean cores off South Africa’s coast have been linked to increased winter rainfall^74^. Regional records show a sharp humid spell around 5-6 ka^70,72,73^. A lower ratio of summer rain, marked drying, seasonal shift in moisture availability to winter rainfall, or both, could be drivers.

The late Holocene (Unit B, 1710–3170 BP uncalibrated) biomarker results agree with the pollen studies showing more arid conditions^28,57^. The marked change in sedimentary environment is also supported by a shift in MS and ARM. C_3_ plants of various kinds and pollen from CAM plants, such as Aizoaceae, can be found in the Holocene pollen at FLB. However, we do not have sufficient preservation for biomarker isotopes in these units. The δ^13^C results at LBD show higher values in the two uppermost samples, potentially indicating increased C_4_ plants in the later part of the Holocene. Poaceae and Cyperaceae pollen concentrations are highest in the uppermost LBD sample (Table S10), indicating grasses and sedges are present, while P_aq_ at LBD indicates a combination of terrestrial and emergent macrophytes throughout.

Our biomarker results show a high C_3_ plant component for most of the Pleistocene units at FLB (except lower Unit M and the lower Unit F sample), as well as the lower units at LBD, with very little or no C_4_ plant presence (18 % in LBD5). This contrasts with the younger Holocene units of these sites, as well as the Holocene sediment samples from the interior of South Africa [e.g., refs. ^41,73^]. However, the values are comparable to results from a study in Lesotho (covering MIS 3 to the mid-Holocene)^75^. This could be the result of a proportionally high production of *n*-alkanes by woody plants compared to grasses and/or the presence of karroid or fynbos elements, CAM plants or C_3_ grasses (SI 3). The woody plants possibly included small shrubs as there is not much pollen evidence of trees in the Pleistocene, which would have been limited as a result of frost^28,60,76,77^. Considering the FLB fauna, we would expect a higher C_4_ grass component as the fauna is composed of typical Florisian Land Mammal Age mammals with specialist grazers such as *Antidorcas bondi* and *Megalotragus priscus*^13,78^. *Antidorcas bondi* has enamel carbon isotope values in the range of a diet dominated by C_4_ plants at FLB^79^. Explanation could be an under-representation of C_4_ grasses in our samples, a seasonal availability of C_4_ grasses^80^ or it could be that *A. bondi* was highly selective of C_4_ grass even in shrubby vegetation. A scenario of year-round production of grass, which implies weak rainfall seasonality, similar to a year-round rainfall zone^78^ is supported by the low δD values (except lower Unit M and perhaps Unit E), which would support a change in rainfall source and/or amount^45,48^. The possibility of more Atlantic winter rainfall would also influence the δ^13^C values and be advantageous for C_3_ plant growth, particularly C_3_ grasses. In contrast, comparably higher δD values in the region in Holocene samples^42,75,81^ could be the result of a strong summer rainfall regime.

The human tooth discovered with the cranium was found in a spring vent of white sand and dated to 259 ± 35 ka using ESR^19^. According to the description of the original excavator^82^, the spring vent cut through Peat I (Units N and O) and was sealed by the Green Sand (Unit M). This means that the human remains would be younger than Peat I, for which our OSL ages obtained on quartz indicate that the top (FLB2) is dated to 198 ± 14 ka (Unit N), and older than the Green Sand, dated to 168 ± 14 ka (FLB3) (Fig. 6). Our ages provide a chronological range for the human remains, ranging from 212 ka to 154 ka, considering the error at 1 σ. This age range appears significantly younger than the ESR age obtained on the human remains, of 259 ± 35 ka for the human tooth^19^, but the two overlap at 2 σ. In their study, Grün et al.^19^ dated two sets of teeth: one was collected in situ from Test Pit 3, the other from the Spring Collection. Little information is provided regarding the calculation of the dose rate, although we can assume that sediment matching with the teeth from Test Pit 3 was collected and measured, and that in situ measurements were conducted, as it was done for the OSL samples analysed in the same study. The other set includes fauna found in the spring vents (i.e., Middle Pleistocene sediments), but their stratigraphic position is unknown. For the dose rate calculation, the same sediment sample (from a hippopotamus mandible) was used for all teeth. Ages are scattered for both sets and were interpreted as the result of material reworking due to spring activity. However, we may also consider issues with dose rate reconstruction, as only one sediment sample was used to derive the dose rate for all teeth from the spring collection, which likely came from different locations within the site since they were collected during an excavation period spanning the 1910s to the 1980s. Only the ESR age estimate of 121 ± 6 ka obtained for the top of Peat II was considered accurate by the authors (Fig. 6). Regarding the OSL ages, ranging from 279 ± 47 ka for Unit P to 146 ± 15 ka for Unit F (Fig. 6), the authors pointed out that they are affected by saturation issues. Their OSL ages place Peat I at 281 ± 73 ka (Units N/O) and the Green Sand (Unit M) at 157 ± 21 ka. Nonetheless, no information is provided regarding the grain size that was analysed or the results of dose recovery tests. In our study, we focused on the most representative fraction, i.e., 4-11 µm for Peat I  (Units O and N) and 100-140 µm for the Green Sand (Unit M), and we give more credit to our OSL ages than to the pIRIR_225_, pIRIR_290_ and TT-OSL ages considering the difficulties in measuring fading rates for the pIRIR_225_ analyses, the issues of the residual dose that may overestimate the ages calculated using the pIRIR_290_ protocol, and the unsatisfactory dose recovery test results of the TT-OSL analyses. Considering the large error range of the previous OSL ages^19^, their age estimates for Units M and O are comparable with our OSL age results. Regarding the human tooth that was directly dated, the same concern about dose rate reconstruction as for the spring collection applies: the dose rate was derived from “the average concentration of radioactive elements of white sand layers”^19^, i.e., Unit F, which is located at least 2 m above the transition between Peat I and the Green Sand. Moreover, the uranium content in the tooth was not measured, and the authors^19^ assumed for the age calculation that it falls within the range of the dental tissues from the spring collection (≤ 268 ppb). A weighted mean age of 259 ± 35 ka was considered for the human remains. Taking into consideration the issues encountered with dose rate reconstruction (sediment and dental tissue) for the human tooth, we would consider this age with caution. The validity of this age was recently questioned on methodological grounds^83^. Moreover, our OSL ages for Units N (198 ± 14 ka) and M (168 ± 14 ka) are similar to the preferred ages presented in a recent dissertation by Pinder^84^, of 191 ± 12 ka (Unit N) and 149 ± 13 ka (Unit M) using multi-grain TT-OSL. However, for Unit O, they obtained a multi-grain TT-OSL age of 136 ± 8 ka and an pIRIR_250_ age of 158 ± 8 ka that do not follow the stratigraphic order, and are younger than all our ages obtained for this unit (205 ± 19 ka, OSL; 278 ± 26 ka, TT-OSL; 244 ± 26 ka, pIRIR_225_; 204 ± 23 ka, pIRIR_290_). Interestingly, our TT-OSL ages all seem to be overestimated compared to those obtained by Pinder^84^. This may be explained by the different protocols used (Arnold et al.^85^, in Pinder^84^ and Porat et al.^86^ in our study). Considering our OSL ages, we can conclude that Units O to M were deposited during a short time interval, spanning MIS 7 to 6, and suggest an age for the human remains likely coeval with the transition between MIS 7 and 6 and/or MIS 6.

The lithic technologies and faunal assemblages associated with the main MSA occupation in Unit F and the earlier MSA horizon in Unit M differ in several respects, suggesting that shifts in ecosystem productivity potentially structured human behaviour and technological decision-making at Florisbad. Although no new technological data are presented here, we reassess previously published lithic and faunal descriptions in light of newly presented evidence for environmental change through the Florisbad sequence^23–25,65,68^. Faunal data from the spring collection, treated as a single analytical unit underlying Unit F owing to sample size and stratigraphic resolution constraints, indicate changes in foraging strategies and patterns of landscape use over time. These behavioural differences are plausibly linked to fluctuations in environmental productivity, potentially driven by regional hydro-climatic variability that influenced the distribution and abundance of key resources in the Florisbad region.

Zooarchaeological parameters provide a framework for evaluating how foragers are expected to respond strategically to shifts in environmental productivity^87–89^. Under conditions of reduced productivity, declining foraging returns are expected to promote broader diets, including increased exploitation of lower-ranked prey such as tortoises and small bovids (e.g. steenbok *Raphicerus campestris*), alongside more selective transport of skeletal parts from kill sites. These conditions also tend to favour intensified processing of transported elements, including increased marrow extraction, and a greater representation of juvenile animals in faunal assemblages. In contrast, more productive environments are generally associated with narrower prey spectra and reduced carcass transport, and higher proportions of adult prey.

Technological systems are likewise expected to respond to changes in environmental productivity. In less productive landscapes, toolkits may emphasise enhanced reliability, enabling foragers to maximise returns under sparse and unpredictable conditions^90–92^. Increased residential or logistical mobility, potentially over greater and more linear distances, would further be expected^93,94^, resulting in greater diversity of lithic raw materials represented in assemblages. Elevated mobility may also favour smaller, lighter tools that were intensively resharpened to extend use-life^95,96^. Alternatively, hominins may have selected larger initial blanks capable of sustaining prolonged reduction sequences over extended transport distances, thereby offsetting anticipated future demands for cutting edges^97,98^. Archaeologically, such strategies would be expressed as reduced on-site manufacture and discard, but increased on-site maintenance, with assemblages having substantial numbers of retouched tools, and bipolar cores exhibiting minimal cortex. Conversely, more productive environments are expected to be associated with less mobile strategies, greater on-site manufacture, and less intensively reduced toolkits.

Although excavated over a relatively small area (~3 m²), Unit M has yielded an assemblage of at least 150–200 lithic artefacts^23^. The assemblage is dominated by hornfels but also includes several dolerite artefacts, indicating greater raw-material diversity than observed in overlying Unit F. Elongated blanks and a relatively high frequency of retouch are evident, with scrapers forming the dominant formal tool type. While hornfels is available relatively close to Florisbad, these technological features are consistent with a more curated technological strategy, in which transported blanks were shaped, maintained, and extended in use on site. Such behaviour suggests longer tool use-lives and mobility strategies involving the transport and conservation of raw material. Stratigraphically, this technological pattern coincides with faunal assemblages associated with the spring vent and with the vegetation reconstruction presented in the current study, together suggesting a landscape structured around wetlands and potentially relatively higher abundances of browsing and mixed-feeding fauna. The spring fauna shows no clear evidence of anthropogenic fragmentation or cut-marking^68^; however, inferences regarding carcass transport remain limited, as the spring collection integrates material from several stratigraphic layers underlying Unit F rather than Unit M alone. Within these constraints, the degree of lithic curation observed in Unit M may reflect adaptive responses to a potentially less productive ecosystem relative to that associated with Unit F.

In contrast, Unit F contains thousands of artefacts, including abundant small debitage and multiple refitting sets indicative of systematic on-site tool production^26^. The assemblage is composed exclusively of hornfels, as mentioned, a locally available but variable raw material. Triangular blanks dominate, while retouch frequencies are low relative to Unit M, and formal tools are rare. Instead, expedient flake production is closely associated with direct evidence for food processing and animal consumption on-site, including an *in*-*situ* hearth with burned bone, extensive cut-marking on faunal remains, and evidence for intensive marrow extraction from long bones, particularly of the extinct blesbok *Damaliscus niro*^24^. The absence of carnivore modification on faunal remains from Unit F, contrasting sharply with the abundant carnivore activity evident in the spring vent assemblage^68^, strengthens the interpretation of human agency in carcass processing in Unit F.

Together, these features point to a more expedient technological strategy during the formation of Unit F, characterised by local acquisition, use, and discard of raw materials within activity-rich occupations along a lake-margin setting. The faunal assemblage of Unit F, dominated by grazers and exhibiting a narrower prey spectrum than the older spring fauna, alongside a stronger C_4_ plant signal, suggests a more open grassland–wetland environment, potentially relatively more productive for hominins.

Taken together, the contrasts between Units M and F potentially reflect shifts in both ecological regimes and human responses to changing ecosystems. During the formation of Unit M, early *Homo sapiens* appear to have operated within fluctuating environmental conditions, including the development of a cooler ecosystem, but relatively low abundances of mixed-feeding fauna such as *Antidorcas marsupialis* (which is not present in Unit F). In this context, tool transport and curation likely played a role in managing subsistence risk. This phase was followed by a shift toward summer rainfall dominance during the later stages of Unit M, accompanied by seasonal drying of the Florisbad spring lake and a short-lived increase in C_4_ grasses.

By contrast, Unit F reflects a shift toward more intensive localised activities, including systematic carcass processing and expedient flake production on-site. Unit F, within MIS 5, shows strong indicators of environmental conditions not seen in modern biomes in South Africa. Differences between Units M and F in raw-material transport, tool maintenance, and discard strategies potentially underscore the role of ecosystem change in shaping behavioural flexibility through time at Florisbad. Technological systems were adjusted to the opportunities and constraints imposed by different environmental contexts.

## Methods

### Extraction of *n*-alkanes

Sediment samples taken from FLB include 24 samples from the Dreyer Section (Fig. 6, Fig. S2). Samples were collected directly from the exposed profile after cutting back the section to avoid contamination and packaged into aluminium foil. Intervals of every 10-20 cm were used, pending on the thickness of the sedimentary unit. Additional samples include one sample from the top of Test Pit 3 and one sample (FLB24) from the modern wetland at the site, both to create a modern biomarker signal for comparison (Fig. 1). At LBD, five sediment samples were taken from the exposed profile between 0.4 and 1.3 m in depth during excavation (Fig. 5). Fieldwork was conducted under the auspices of the National Museum Bloemfontein. Export of samples took place under permits n. 4099 (Florisbad) and n. 4211 (Lombard’s Drift) issued by the South African Heritage Resources Agency (SAHRA). Excavations at Lombard’s Drift were conducted after obtaining the necessary permits from SAHRA (n. 4030) and the Free State Province Department of Economic, Small Business Development, Tourism and Environmental Affairs.

All samples were dried in an oven at 60 °C for 48 h before being sieved through a 1 mm mesh and homogenised by hand using an agate mortar and pestle. 21 samples from FLB and all samples from LBD have been extracted and quantified, following a modified protocol from Wang et al.^99^. Sediment was weighed into sizes of 22 cm^3^ (3 g of sediment; 3-4 vials per sample) or 66 cm^3^ (12-16 g of sediment; one vial per sample) vials and organics were extracted using an Accelerant Solvent Extractor (ASE-200, Dionex for 22 cm^3^ vials; ASE-350, Dionix for 66 cm^3^ vials) at 100 bar and 100˚C using a 9:1 (v = v) mixture of dichloromethane (DCM) and methanol. The extracted organic solution was filtered through activated silica gel and silver nitrate (AgNO_3_) coated silica gel via column chromatography to isolate *n*-alkanes. The product was dried under a stream of nitrogen gas and transferred into vials for gas chromatography.

Individual *n*-alkane homologues were identified with an Agilent 6890 N gas chromatograph equipped with a Restek XTI-5 capillary column (30 m x 320 μm x 0.25 μm) based on the comparison of their retention times in comparison to a standard containing the *n*-alkane homologues *n*-C_20_, *n*-C_24_, *n*-C_28_, *n*-C_32_, *n*-C_36_, *n*-C_38_ and *n*-C_40_ of known concentration. This standard was used to calculate a calibration factor for quantification of the samples and was additionally measured at the start and end of every batch of six samples. All the samples were measured in triplicate. The following GC-FID temperature program was used for the FLB samples (except FLB24): the oven was maintained for 1 min at an initial temperature of 50 °C, then the temperature was increased to 300 °C (10 min hold) before a further increase to 330 °C (10 min hold). For sample FLB24 and the samples from LBD, the following GC-FID temperature program was used: the oven was maintained for 1 min at an initial temperature of 50 °C, then the temperature was increased to 150 °C (5 min hold) before a further increase to 330 °C (5 min hold). The carbon preference index (CPI) has been calculated using the following formula:

CPI_27-33_ = 0.5*[(C_27_ + C_29_ + C_31_ + C_33_) / (C_26_ + C_28_ + C_30_ + C_32_)]  + [(C_27_ + C_29_ + C_31_ + C_33_) / (C_28_ + C_30_ + C_32_ + C_34_)].

Average chain length (ACL) was calculated using the following formula:

ACL_27-33_ = (27 × C_27_ + 28 × C_28_ + 29 × C_29_ + 30 × C_30_ + 31 × C_31_ + 32 × C_32_ + 33 × C_33_) / (C_27_ + C_28_ + C_29_ + C_30_ + C_31_ + C_32_ + C_33_).

*Norm*31 is calculated as: *Norm*31 = C_31_ / (C_29_ + C_31_) and *Norm*33 as: *Norm*33 = C_33_ / (C_29_ + C_33_).

The fraction of C_4_ for C_31_vegetation, *f*_C4_, is calculated using a linear mixing model^100^, with the All Africa endmembers for δ^13^C_C3_ and δ^13^C_C4_^101^ respectively.

### Stable isotope measurements

The δD and δ^13^C values of terrestrial-sourced *n*-alkane homologues of sufficient concentration (i.e. *n*-C_27_, *n*-C_29_, *n*-C_31_, and *n*-C_33_) were analysed using gas chromatography-isotope ratio mass spectrometry (GC-IRMS) at the Leibniz Laboratory for Radiometric Dating and Isotope Research at Kiel University. Samples were measured on an Agilent 6890 gas chromatograph equipped with a Gerstel KAS 4 PTV injector and an Agilent DB-5 capillary column (30 m x 250 μm x 0.25 μm) coupled to a Thermo Scientific MAT 253 isotope ratio mass spectrometer (IRMS). For analyzing carbon or hydrogen isotopes following GC temperature program was used: 50 °C for 5 min, with 40 °C/min to 240 °C, with 20 °C/min to 280 °C, with 10 °C/min to 325 °C, 325 °C for 20 min. Depending on the *n*-alkane concentration, 10.0 μl and between 10.0 and 20.0 μl of each sample for δ^13^C and δD, respectively, has been injected 2–4 times in order to achieve a statistically robust analytical error for each *n*-alkane homologue. To allow large volume injections (LVI), the injector was used in solvent vent mode. The H3 + factor during the measurement period was 10.39 ± 0.17 ppm/nA (*n* = 11). Using Arndt Schimmelmann’s A7 reference mixture from 2017, the δD and δ^13^C values are reported relative to Vienna Standard Mean Ocean Water (‰ VSMOW) and Vienna Pee Dee Belemnite (‰ VPDB) scales, respectively.

#### Magnetic properties

The magnetic susceptibility (MS) and the anhysteretic remanent magnetisation (ARM) of sediment samples was measured in order to identify changes in sediment supply associated with breaks in the sedimentation process or erosional events, and the concentration of magnetite in sediments, respectively (e.g. Lyons et al.^102^). At FLB, 51 samples were collected from the Dreyer Section every 5-10 cm. Of these, 28 samples were taken from the Upper Dreyer Section, while 23 were collected from the Lower Dreyer Section. At LBD, five samples were taken every 20-30 cm in order to cover the four sedimentary units that were identified in the field and analysed only for MS.

Samples for MS were prepared by transferring a few grams of sediment into plastic holders, which were then placed in the MS reader. Two analyses were conducted for each sample and the results were averaged. Low field, bulk magnetic susceptibility was measured with an MFK1-FA (AGICO) at a frequency of 976 Hz, after weighing the samples. Samples for ARM were prepared by mixing a few grams of sediment with 50 % sodium silicate inside plastic holders, a step necessary to stabilise unconsolidated samples during analysis. The mass-normalised ARM was obtained by dividing the magnetic moment of the sample by its mass (expressed in kg). The ARM was designed to isolate the fine-grained magnetite concentration of the mass normalized samples using a 100mT AF field and a biasing 0.1mT DC field as a function of stratigraphic depth. Measurements were carried out with a 3-axis, 4.2 cm-access SQUID magnetometer (755-4 K Superconducting Rock Magnetometer, 2 G Enterprises).

### Luminescence dating

Sediment samples from Unit O (early MSA, FLB1, base of Peat I), N (early MSA, FLB2, top of Peat I) and M (MSA, FLB3, Green Sand) at FLB (Dreyer Section) and from the peat layer at the base of the sequence for LBD (LBD4) were collected in lightproof bags for optically stimulated luminescence (OSL), thermally transferred-OSL (TT-OSL) and post-infrared infrared stimulated luminescence (pIRIR_225_ and pIRIR_290_) dating. The gamma dose rate was measured in situ using a portable gamma-ray spectrometer connected to a LaBr probe (Inspector 1000, Canberra) at the same location as the samples (Fig. 2) and the data treated using the threshold approach^103^.

Considering the sedimentology of the dated units, luminescence measurements were conducted on the most dominant grain size (4-11 µm for FLB1 and FLB2; 100-140 µm for FLB3 and 100-120 µm for LBD4) at Archéosciences Bordeaux, France, using a TL/OSL Lexsyg Research reader for multigrain (MG) measurements and a TL/OSL Risø reader for the single-grain measurements. The samples were wet-sieved at 140, 120, 100 and 20 µm. For FLB1 and FLB2, the 4–11 μm fraction was extracted by decantation from the ≤20 µm fraction using Atterberg according to Stokes’s law. The organic matter and carbonates were eliminated using H_2_O_2_ (30 %) and HCl (10 %), respectively on both 4-11 µm and 100-120 or 100-140 µm fractions.

The 4-11 µm fraction was divided in two; one was kept intact (polymineral fraction for the pIRIR protocols), and the other one was treated using H_2_SiF_6_ (31 %) over one week and rinsed with HCl (10 %) to eliminate feldspars (quartz fraction for OSL and TT-OSL protocols).

The quartz and feldspars from the 100-120 or 100-140 µm fraction were extracted using density separation with heavy liquid (heteropolytungstate of sodium). Three separations were conducted, the first one to extract heavy minerals (d = 2.72 g/cm^3^), the second one to separate quartz and feldspars (d = 2.62 g/cm^3^), and the third one to separate plagioclase and potassium feldspars (d = 2.58 g/cm^3^). To avoid potential feldspar contamination, the quartz fraction was treated using H_2_SiF_6_ (31 %) over one week and rinsed with HCl (10 %).

The MG luminescence measurements were conducted using the single aliquot regeneration (SAR) protocol^104^ on 8 mm aliquots for the 4-11 µm fraction, and on 1 mm aliquots for the 100-120 and 100-140 µm fractions. For the quartz, the standard OSL procedure was applied (Table S5), as well as a simplified TT-OSL protocol^86^ (Table S6). The signal was detected on a TL/OSL Lexsyg Research reader using a combination of UV filters (Schott BG 3-Glas-3 mm in conjunction with a Delta-BP 365/50 EX-Interference 5 mm). The feldspars were measured following the protocol at 225 °C (pIRIR_225_)^35^ (Table S7) and at 290 °C (pIRIR_290_)^105^ (Table S8). The IRSL signal was measured on a TL/OSL Lexsyg Research reader with a combination of optical filters (Schott-BG 39-Glas-3 mm; AHF-Brightline HC 414/46-Interference-3.5 mm). Fading measurements were conducted following Auclair et al.^34^ and the fading rates was calculated according to Huntley and Lamothe^33^ using the calc_FadingCorr() function in the R package Luminescence^106^.

Considering that for FLB3, the conventional OSL signal was close to saturation (Fig. S11), SG measurements were conducted using standard 150 μm holes-disks. Moreover, the preliminary MG TT-OSL data being scattered for this sample, microaliquots (MA) were measured, i.e., the 100-140 µm quartz grains were mounted on the standard 300 μm holes-disk, resulting in several grains per hole to increase the yield^107^. The SG and MA measurements were conducted on a Risø OSL/TL reader using the protocol displayed in Table S9 for the OSL and in Table S10 for the TT-OSL. A green laser was used to measure 1500 grains and the signal was detected using Hoya U340 filters. For LBD4, only MG OSL measurements were conducted due to low overdispersion values (Table S5).

The data were treated using Analyst v. 4.57^108^. Both MG and SG data were selected using the following criteria: a recycling ratio limit of 10 %; a recuperation <5 % of the natural signal; a maximum test dose error of 10 %; and a test dose signal > 3 sigma above background. The MG OSL and TT-OSL signals were integrated using the first 0.3 seconds and the background was subtracted using the last 20 seconds. The MG pIRIR_225_ signal was integrated using the first 1.5 seconds and the background subtracted using the last 20 seconds. The MG pIRIR_290_ signal was integrated using the first 2.5 seconds and the background subtracted using the last 50 seconds. The SG OSL signal was integrated using the first 0.07 seconds and the background was subtracted using the last 0.25 seconds. The MA TT-OSL signal was integrated using the first 0.13 seconds and the background subtracted using the last 0.50 seconds. The DRC were obtained using an exponential function for the SG OSL and MG and MA TT-OSL measurements and an exponential + linear function for the MG OSL and pIRIR measurements.

Dose recovery tests (DRT) were performed to assess the reliability of the results on all samples: for the OSL protocol, three to seven aliquots (for MG measurements) and five SG disks (500 grains measured) were bleached two minutes in a solar simulator (Hönle 500) and a dose of 364 Gy (FLB1 MG), 308 Gy (FLB2 MG), 195 Gy (FLB3 SG) and 25 Gy (LBD4 MG) was given before applying the SAR protocol. For the MG TT-OSL and the pIRIR_290_ protocols, the DRTs were obtained on three to five aliquots after adding a dose on the natural one of 112 Gy, 146 Gy and 56 Gy for the TT-OSL, pIRIR_225_ and pIRIR_290_ measurements, respectively. For the MA TT-OSL protocol, seven 300 µm holes-disks, containing several 100-140 µm quartz grains, were bleached for 24 h. A dose of 379 Gy was given to five of them to measure the DRT and the two remaining disks were used to measure the residual dose, which appears negligible for most of the grain positions. The grains presented no signal or a dim one; only 7/200 grains gave a suitable signal for calculating the residual dose, providing an average value of 22 Gy.

The alpha and beta dose rate were derived from U, Th, and K content measured using a laboratory gamma-ray spectrometer equipped with a high-resolution, broad energy Ge (BEGe) detector. The in situ gamma dosimetry data were treated using the “threshold” technique^103^. The water content (water/dry mass of sediment %) was measured before and after drying the samples in an oven for one week and used for age calculation.

The cosmic dose rate was estimated from the current burial depths below the surface using the equation from Prescott and Hutton^109^. The ages were calculated using the conversion factors of Guérin et al.^110^, taking into account the beta absorption factors of Guérin et al.^111^ and the alpha attenuation factors of Brennan et al.^112^.

### Pollen and charcoal analysis

Five sediment samples from the LBD excavation were collected for pollen and charcoal analysis. Standard palynological methods of HCl, KOH and HF digestion were used for the extraction of pollen palynomorphs similar to that of the Holocene samples^57^. Heavy liquid mineral separation with ZnCl_2_ solution (density 2) was used to extract the palynomorphs. Pollen and charcoal concentrations were determined using known doses of exotic *Lycopodium* spores from Lund University (Batch no 280521291)^113^. The prepared samples were mounted in glycerine jelly on microscope slides. The slides were scanned for pollen and microscopic charcoal under 40X magnification, or 100X oil immersion for more difficult identifications. For accurate determination of the vegetation, the aim was to count 300 pollen grains and spores. Pollen was identified to family, genus, and, where possible, species level. Charcoal counts were divided into small (10-100 µm) and large (>100 µm) classes.

## Supplementary information

Transparent Peer Review file

Supplementary Material

Description of Additional Supplementary files

Supplementary Data 1

## Data Availability

All data that support the findings of this study are available in Zenodo with the identifier https://doi.org/10.5281/zenodo.21981316^114^.
