# Supplementary material for: Non-analog environments supported early Homo sapiens in southern Africa 200 000 years ago
Source: Commun Earth Environ. 2026 Oct 30;7(1):829. doi: 10.1038/s43247-026-04116-4 (PMC13722590; doi:10.1038/s43247-026-04116-4)
Supplement: Supplementary file 1 [file 43247_2026_4116_MOESM1_ESM.pdf]

nature portfolio

Peer Review File

Non-analog environments supported early *Homo sapiens* in southern Africa 200 000 years ago

Corresponding Author: Dr Michaela Ecker

This file contains all reviewer reports in order by version, followed by all author rebuttals in order by version.

Version 0:

Reviewer comments:

Reviewer #1

(Remarks to the Author)
The Florisbad site in South Africa is of considerable paleoanthropological interest because it preserves important archaeological, human fossil, and associated faunal remains dating to the Middle and Late Pleistocene. Here, the authors present new chronological, sedimentological, and environmental data for the site, in addition to a nearby Holocene archaeological site (Lombard's Drift). In doing so, they provide a revised age for the Florisbad human cranium and argue that early human populations in the region were embedded in non-analog ecosystems (i.e., environments unlike any found today), and propose that environmental changes drove behavioral shifts evident in the archaeological record from the site. The new data provided here are important and merit publication, though I believe several of the key interpretations will require strengthening. This includes a more rigorous evaluation of the paleoenvironmental data as well as developing stronger links between environment and lithic technology.

Analysis of environmental data: The d13C and dD values from leaf waxes feature prominently in the argument that the Pleistocene environments are unlike any today (i.e., that they are non-analog). I believe a compelling argument would require more detailed analyses of these data. For example:

-"At both sites, the modern samples are closest to the modern Grassland values in[31]." And "The Unit I sample (FLB-LD18) has the lowest measured δ13C (below -30‰), with the weighted average of sample FLB-LD18 in the range of values for the modern Fynbos Biome." Given the amount of overlap across biomes, I'd like to see a more detailed comparison of how the modern (and paleo) values compare to modern values from southern Africa today (e.g., plot the data for Florisbad relative to values from modern biomes). There is a rich dataset provided the reference cited here.

-"In contrast, the Pleistocene samples are not within this range, except for lower Unit M. The ACL27-33, Norm31 and Norm33 indices all indicate a very different range for Units N to E, compared to the Holocene (Figure S.18). This suggests non-analog environments for most parts of the Pleistocene with a different vegetation and/or rainfall structure than today" This needs strengthening. A difference between Pleistocene and Holocene (or between Pleistocene and any single modern biome) does not automatically imply non-analog environments in the Pleistocene. Presumably, a non-analog environment would include combinations of d13C and dD values (in parallel w/ other paleoenvironmental data) unlike any observed in southern Africa today, hinting at a unique combination of climate and vegetation. This may indeed be the case, but I'm not yet convinced. Can you demonstrate this through a more detailed comparison of Pleistocene to present-day southern Africa? This may entail further analysis of the isotopic data, perhaps in parallel with consideration with associated lines of paleoenvironmental evidence (e.g., pollen, fauna). Also, non-analog in what way? Can you clarify how the environments were unlike anything found in the region today?

"This suggests that at least the lower units around Unit O during Middle Pleistocene occupation at Florisbad have, in addition to C4 grasses, a substantial C3 plant component, probably fynbos-like and karroid vegetation associated with cooler climates, of which only the latter is prominent in the early Holocene layers. This combination of vegetation indicates a non-analog environment that different seasonal rainfall patterns would drive compared to today." Can you elaborate on how seasonal changes in rainfall would drive this? Also, some of the fynbos-associated taxa (e.g., Ericaceae and Restionaceae) do occur occasionally in the nearby region (Chevalier et al 2021)—so are we sure that their presence alongside C4 grasses is non-analog? I'm not arguing that the Pleistocene environment was analogous to today, but I do need more convincing that it was not.

Human adaptation to non-analog environments: The authors propose that environmental changes demonstrated at Florisbad account for changes in the MSA lithic assemblages at the site:
"The assemblage is dominated by hornfels, with elongated blanks and a relatively high frequency of retouch, including scrapers as the dominant formal tool type. These features point to a more curated technological strategy, where transported blanks were shaped and maintained on site, consistent with a technological emphasis on longer tool use-lives and mobility strategies that involved carrying and conserving raw material. This technological behaviour aligns with the spring vent associated faunal spectrums and our vegetation reconstruction, suggesting a landscape where wetlands and possibly higher numbers of browsing fauna structured human activities. In this context, curation and transport of tools may have been adaptive to a more spatially constrained and patchy ecosystem."

What is the link between patchy ecosystems and the observed technological strategies? Robust links between environment and behavior demand some sort of theoretical framework linking one to the other. There is a large, theoretically-informed literature establishing links between environment and lithic technology, none of which is considered here. I encourage the authors to engage with this literature to strengthen the proposed link between environment and lithic technology.

Similar questions apply to the subsequent paragraph, where the authors argue that "In such an ecosystem, reliable and predictable access to resources appears to have supported shorter tool use-lives, reduced curation and opportunistic on-site tool manufacture." This reads to me as a post hoc interpretation. What is the link between "reliable and predictable access to resources" and the observed technologies? The authors need to engage with the relevant literature to make a stronger argument here.

Other edits, questions, and suggestions:
Abstract: "We observe contrasting, non-analogous environments during the Middle and Late Pleistocene." Non-analog in what way? Can you be more specific?

Abstract: "It also provides the foundation to evaluate their cultural adaptation strategies through time." How so? Can you be more specific?

Introduction: insert comma "…seems contradictory at some levels[comma here] with interpretations requiring different taphonomic histories for vegetation reconstruction"

Results: "In contrast, in the Pleistocene samples, C33 is the dominant n-alkane with C31 the second most abundant n-alkane" As you did for the preceding sentence (i.e., "indicating a mainly terrestrial plan wax source"), can you briefly indicate what this means?

Results: "However, no LBD sample shows the disproportional high C33" use subscript for 33

Results: in the text (e.g., "However, while 3TP1 ranges from -28.1‰ (C25) to –23.8‰ (C33), FLB24 shows a different trend in which homologue is highest, ranging from -25.4‰ (C25) to –28.3‰ (C29)" and figures (e.g., Figure 3), would it be possible to also provide calculations for percent C4 biomass? This would facilitate interpretation of the data. Polissar et al. (2019) give an example of how to do this.

Discussion, The Florisbad Sequence: "At both sites, the modern samples are closest to the modern Grassland values…" Modern samples of what?

Discussion, The Florisbad sequence: "Further hints at fynbos-type contributions are the Norm33 index values for sample FLB-LD24." How so? Please clarify.

Discussion, The Florisbad sequence: "This could suggest interglacial climatic conditions with strong evaporation and associated vegetation comparable to today." Important to keep in mind that precipitation changes may not solely follow glacial-interglacial cycles. In southeastern Africa, evidence from lacustrine sediments from Tswaing Crater and the marine core CD154 10–06 P Fe/K record both indicate a strong precessional signal (Chase 2021)

Discussion, The Florisbad sequence: "The faunal isotopes also show lower δ13C values in the enamel of grazers than comparable species today indicating more C3 plant presence [15]." But note that most grazer d13C values do not vary in relation to the availability of woody cover (Robinson et al 2021). Might this indicate consumption of C3 grasses?

Discussion, The Florisbad sequence: "The fauna in Unit F shows only one mixed feeder (a steenbok) and no browsers or other mixed-feeders such as eland and modern springbok" Provide genus and species
Discussion, The Florisbad sequence: "Explanation could be an under-representation of C4 grasses in our samples or it could be that A. bondi was highly selective of C4 grass even in a shrubby vegetation. [65] suggested a scenario of year-round production of grass, which implies weak rainfall seasonality, similar to a year-round rainfall zone." There is also broader evidence that loss of grassland species across the Pleistocene-Holocene transition was not associated with declining availability of grasses on the landscape (Norwood et al 2025), suggesting that what matters here is not extent of grassy vegetation but instead its seasonal availability. And wouldn't reduced seasonality, which means some rainfall in the winter, perhaps lead to presence of C3 grasses? This might explain the low d13C values of some fossil grazers from Florisbad (Codron et al. 2008)

Discussion, Age of the Florisbad Cranium: “Our ages provide a chronological range for the human remains, ranging from 212 ka to 130 ka, considering the error at 1 σ, significantly younger than the age obtained on the human remains by[19]” But considering the two-sigma overlap of previous ages and yours, perhaps they are not inconsistent.

References

Chase B. 2021. Orbital forcing in southern Africa: Towards a conceptual model for predicting deep time environmental change from an incomplete proxy record. Quaternary Science Reviews 265
Codron, D., Brink, J. S., Rossouw, L., & Clauss, M. (2008). The evolution of ecological specialization in southern African ungulates: competition-or physical environmental turnover?. Oikos, 117(3), 344-353.
Chevalier M, Chase BM, Quick LJ, Scott L. 2021. An atlas of southern African pollen types and their climatic affinities. In Quaternary Vegetation Dynamics: The African Pollen Database, ed. J Runge, WD Gosling, A-M Lézine, L Scott, pp. 239-58. Leiden: CRC Press
Norwood A, Rowan J, Faith J. 2025. Reconciling the environmental implications of late Quaternary faunal and pollen records in southern Africa. Quaternary Science Advances 17
Polissar PJ, Rose C, Uno KT, Phelps SR, deMenocal P. 2019. Synchronous rise of African C4 ecosystems 10 million years ago in the absence of aridification. Nature Geoscience 12: 657-60
Robinson J, Rowan J, Barr W, Sponheimer M. 2021. Intrataxonomic trends in herbivore enamel δ13C are decoupled from ecosystem woody cover. Nature Ecology & Evolution 5: 995-+

Reviewer #3

(Remarks to the Author)
This paper seeks to reconstruct the environments represented through the sediment sequence at the South African archaeological site of Florisbad using a variety of proxy data, but focussing on plant wax n-alkanes. In addition, new luminescence dating is conducted at the site. The paper concludes that the Homo sapiens inhabitants of the site lived in non-analogue environments, and that the well-known cranium from the site dates to ~200 ka i.e. significantly younger than published age estimates. The results presented in this paper are important, and should be of considerable interest to readers of Communications Earth and Environment. However, I believe that a major re-write of the luminescence dating sections, is required prior to publication. It should be noted that while I find the explanation of the leaf wax data convincing, I am not a specialist in this field.

Luminescence dating

My main concern with this paper is that the luminescence dating work presented in a rather disorganised manner, and some of the assumptions/decisions made have not been properly justified or explained. Although individually rather picky, collectively the concerns outlined below limit my ability to interpret the luminescence data, and therefore my confidence in the ages presented.

First paragraph of “Luminescence dating”. It is stated that FLB3 was measured using single-grain discs, but it is not clear whether the sample was measured as single grains. Later in the paper it is stated that the TT-OSL was measured as “micro-aliquots” i.e. more than one grain in each 300 µm sample position, but the OSL signal was measured on single-grains. Given that all of the grains measured for each samples were the same size, does this mean that smaller aperture single-grain discs were used, or is this signal also micro-aliquot? This question is only answered much later in the paper in the “methods” section. Also, the “age results” are said to be computed by the central age model (CAM), but this must in fact be the equivalent dose.

Second paragraph of “Luminescence dating”. The dose recovery data are described poorly, and this is not helped by the presentation of the data in Figures S11-S13. The authors define the dose recovery ratio, and then express the results as percentages. This is both unconventional and unhelpful. Figures S11 and S12 are also unconventional – why present data for individual aliquots when the mean (or CAM) dose recovery ratio (by sample and signal) is more informative? All of the mean/CAM data could be displayed in one figure. Data for sample FLB3 are missing from Fig. S12. The units for the radial axes of figure S13 is wrong – you are plotting dose recovery ration not De. For all radial plats, replace the last sentence of the caption with something more specific such as “Plots were created using the plot_RadialPlot() function in the R package Luminescence. Lastly, there is a literature on the overdispersion of data from quartz samples, which supports the assertion that 28% is (just) consistent with a well-bleached sample. However, it tends to implicate beta-microdosimetry not “intrinsic variations” of luminescence characteristics as suggested here.

In the “luminescence dating” section the authors state that they give “more credibility” to the quartz OSL ages, which is defensible, but they then sometimes discuss the pIRIR ages later in the paper, only to dismiss them. It would be tidier to make a case for only believing the OSL ages somewhere in this section, and then not referring to the other age estimates later.

The authors make a strong case for reassessing the age of the Florisbad cranium. However, the authors reject their own TT-OSL data on the basis that the dose recovery results are poor and the TT-OSL ages are older than those produced by conventional OSL. They then cite three TT-OSL ages from a doctoral thesis (Pinder, 2021), two of which are very similar to their conventional OSL ages. Why did Pinder get the “correct” ages using TT-OSL, whereas their TT-OSL dates are interpreted as overestimates for the same sedimentary units? As a minimum, this anomaly should be acknowledged, and

ideally some attempt to explain it should be provided.

Lastly, and most importantly, the authors need to provide much stronger evidence that their quartz OSL ages finite and not minimum ages. All three of the Florisbad OSL samples yielded high equivalent dose values (366, 303 and 185 Gy in age order), at least two of which could easily be "saturated" i.e. beyond the point at which the luminescence signal ceases to grow predictably with increasing dose. There is a considerable literature on this topic, but many studies (e.g. Chapot et al., 2012, Radiation Measurements) suggest that as the OSL signal nears saturation, age underestimates occur. This is because the laboratory induced luminescence is larger than that observed for the same dose administered in nature. The saturation limit is sample dependant, but Chapot observed this effect above ~150 Gy, so it could easily be present in the OSL dates presented in this paper. Successful dose recovery does not test for this phenomenon, since all doses are administered in the laboratory. The authors themselves make reference to problems associated with quartz saturation in a previous study at Florisbad (Grun et al., 1996) and in relation to one of their own samples, FLB3, which was stated to be "close to saturation". Interestingly, FLB3 has the lowest equivalent dose (185 Gy) of the three Florisbad samples, so it is not clear why the other samples should not be closer to saturation. Also, the authors state that they performed single-grain analysis on sample FLB3 because of its closeness to saturation, but don't explain the logic of this decision. It is probably a defensible decision, but the reader needs to know their reasoning. Given that the authors wish to revise the accepted age of the Florisbad cranium downwards (i.e. make it younger) on the basis of quartz OSL dating, that quartz OSL dating is known to yield age underestimates as samples approach saturation, the authors need to convince readers that the new younger age is not the result of undiagnosed saturation. I suggest that the following revisions are made:
1) Show growth curves for all three of the Florisbad samples. This is easy and the data already exist. The curve fitting parameter D0 which governs the rate of saturation for a saturating exponential curve should also be stated.
2) Calculate the effect of Do on De by rejecting quartz grains at progressively higher D0 values for your single-grain OSL dataset. A plateau in equivalent dose has been interpreted as indicating the correct value for age determination e.g Guo et al. (2017) Journal of Quaternary Science 32 and Thomsen et al. (2016) Quaternary Geochronology 31. The logic here is that De doesn't change above a D0 value at which saturation does not cause dose underestimates..
3) Measure lower temperature pIRIR signals for the 4-11 µm samples. The authors believe at least two of their pIRIR290 samples to contain unbleached residual doses which cause age overestimates. Since lower temperature pIRIR signals have higher bleaching rates, a well-bleached signal could be found at lower temperatures e.g. pIRIR225. Unfortunately, since fading rates also increase with decreasing temperatures, the authors would need to demonstrate that they weren't simply lowering the measurement temperature until some combination of fading and bleaching gave the "right" ages. This could be done by measuring g-values (fading rates) for each signal. Also, sample FLB1 could be used as a control, since it yielded a pIRIR290 age similar to that for the OSL measurements, and is therefore presumably well-bleached.
The data already exist to implement suggestions 1 and 2, which I regard as essential. The third suggestion requires a lot of additional work, but would considerably strengthen the case for revising the age of the Florisbad cranium.

General points
1) The paper appears to have been written with Harvard style references and then converted to the numerical reference style. This produces awkward text such as a sentence in the "human adaptation to non-analog environments" section which starts "[65] suggested a scenario of year-round production of grass…". I suggest that the authors re-write sentences where the name of the reference is an integral part of the sentence.
2) The style of the paper is occasionally rather colloquial.
3) Radiocarbon ages are not presented in a consistent style, with mean values and both 1 and 2 sigma ranges appearing at various places in the text/figures.

Version 1:

Reviewer comments:

Reviewer #1

(Remarks to the Author)
Thank you for the opportunity to examine the revised version of this manuscript. The authors have made a strong effort to address our feedback, and I believe the result is a stronger paper. In particular, the revisions have helped make a stronger case for the non-analog nature of these Pleistocene ecosystems, and strengthened the interpretation of the archaeological data. I see that one of the previous reviewers was largely concerned with the luminescence ages. The authors have responded to those concerns, but I lack the expertise to assess these revisions.

I have only one (fairly minor) substantive comment with the revisions. The authors suggest that the evidence for intensive marrow extraction and carcass processing in Unit F does not align with expectations for highly productive environments. Bone marrow is key source of fat (especially on wild ungulates that are relatively lean), and ethnographic observations consistently show that hunter-gatherers regularly exploit marrow from long bones. In other words, within-bone nutrients (e.g., marrow) should be relatively highly ranked relative to other portions of an ungulate carcass, so I wouldn't necessarily interpret evidence for long-bone marrow extraction as a sign of intensification or resource stress. In contrast, evidence for intensive processing of body parts with minimal amounts of marrow (e.g., phalanges) or labor-intensive grease extraction might be indicative of intensification. More broadly, zooarchaeological indicators of intensification are perhaps most effectively used when examining trends through space or time--e.g., where one might observe temporal shifts in exploitation of high versus low ranked prey, shifts in prey age structure, and evidence for changes in carcass processing strategies. But it may be harder to interpret a single assemblage like Unit F as a sign of good or bad times (relative to what?)

I encourage the authors to potentially reconsider this point, but otherwise I think this is a solid paper that adds considerably to our understanding of an important site.

And a few minor edits. Apologies if I missed these in the first round of review.
Line 29: “Such an environment” should be “Such environments”
Line 389: spell genus name in full when it begins a sentence: “A. bondi” to “Antidorcas bondi”
Line 469: genus + species needed for steenbok

Open Access This Peer Review File is licensed under a Creative Commons Attribution 4.0 International License, which permits use, sharing, adaptation, distribution and reproduction in any medium or format, as long as you give appropriate credit to the original author(s) and the source, provide a link to the Creative Commons license, and indicate if changes were made.
In cases where reviewers are anonymous, credit should be given to 'Anonymous Referee' and the source.
The images or other third party material in this Peer Review File are included in the article’s Creative Commons license, unless indicated otherwise in a credit line to the material. If material is not included in the article’s Creative Commons license and your intended use is not permitted by statutory regulation or exceeds the permitted use, you will need to obtain permission directly from the copyright holder.
To view a copy of this license, visit https://creativecommons.org/licenses/by/4.0/

Response to reviewers

We thank the reviewers for their time and the insightful and helpful comments. Our detailed response is below in blue. In addition to the requested changes, the following was added/changed:

- we added Balzeau et al. 2026, a recent re-analysis of the Flroisbad cranium that was published after our submission. This matches Grine et al. 2019, who demonstrate that the molar root fits comfortably in the RM^3 socket, which is important for the integrity of the context. Neither analysis does challenge or change our results.

- Small changes to Figure 3: The radiocarbon age in unit D changed to 8170±90 BP. The IRSL ages have been removed and the OSL date for FLB3 has been updated. This follows the reviewer suggestion to only use the dates that we determined as most reliable. The *Norm*31 and *Norm*33 value for the upper unit F sample was deleted as the C_{29} concentration was not high enough to calibrate this index, it was also changed in the respective table accordingly.

REVIEWER COMMENTS:

Reviewer #1 (Remarks to the Author):

The Florisbad site in South Africa is of considerable paleoanthropological interest because it preserves important archaeological, human fossil, and associated faunal remains dating to the Middle and Late Pleistocene. Here, the authors present new chronological, sedimentological, and environmental data for the site, in addition to a nearby Holocene archaeological site (Lombard's Drift). In doing so, they provide a revised age for the Florisbad human cranium and argue that early human populations in the region were embedded in non-analog ecosystems (i.e., environments unlike any found today), and propose that environmental changes drove behavioral shifts evident in the archaeological record from the site. The new data provided here are important and merit publication, though I believe several of the key interpretations will require strengthening. This includes a more rigorous evaluation of the paleoenvironmental data as well as developing stronger links between environment and lithic technology.

Analysis of environmental data: The d13C and dD values from leaf waxes feature prominently in the argument that the Pleistocene environments are unlike any today (i.e., that they are non-analog). I believe a compelling argument would require more detailed analyses of these data. For example:

-"At both sites, the modern samples are closest to the modern Grassland values in[31]." And "The Unit I sample (FLB-LD18) has the lowest measured δ13C (below -30‰), with the weighted average of sample FLB-LD18 in the range of values for the modern Fynbos Biome." Given the amount of overlap across biomes, I'd like to see a more detailed comparison of how the modern (and paleo) values compare to modern values from southern Africa today (e.g., plot the data for Florisbad relative to values from modern biomes). There is a rich dataset provided the reference cited here.

We added a new figure (now Figure 5, see here below) which contains two parts, both to strengthen our argument. 1) Scatterplot of the *Norm*31 and *Norm*33 indices for the Florisbad

and LBD samples as well as modern biomes in South Africa. 2) Scatterplot of the C_{31} $\delta^{13}C$ (‰ VPDB) and δD (‰ VSMOW) values for the Florisbad and LBD samples as well as modern biomes in South Africa and the fraction of C_4 vegetation. We deleted Figure S18 which was a simpler version of Fig. 5 and which we had forgotten to mention in the text. Aditonally, the text in the Discussion part “The Florisbad sequence” was rephrased and Figure 5 cited.

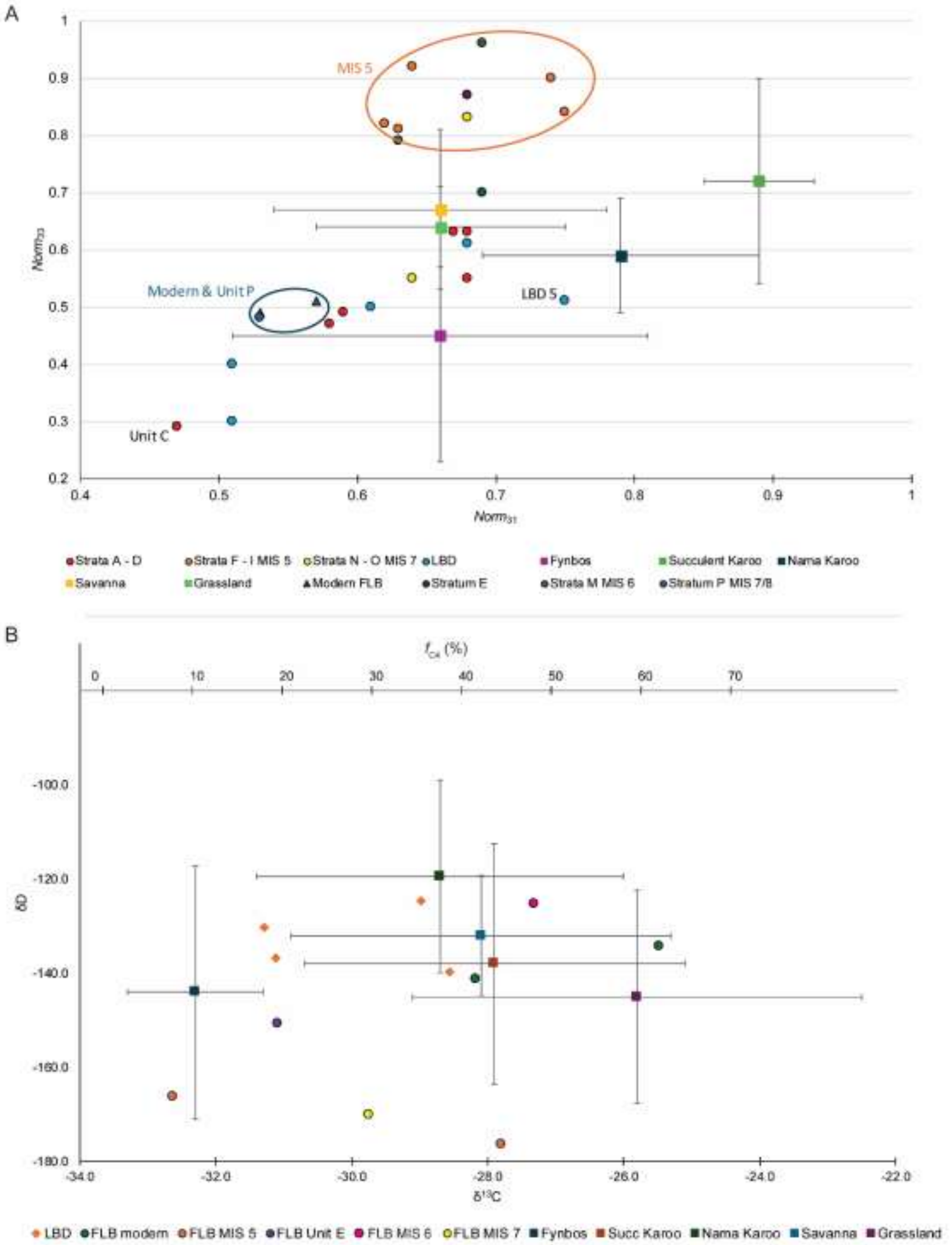

Figure 5. Comparison of the *n*-alkane indices and stable isotope results to modern biomes in South Africa, showing that particularly the MIS 5 and MIS 6 samples do not

fall within modern biomes. A. Scatterplot of the *Norm*31 and *Norm*33 indices for the Florisbad and LBD samples as well as modern biomes in South Africa[39]. B. Scatterplot of the C_{31} $\delta^{13}C$ (‰ VPDB) and δD (‰ VSMOW) values for the Florisbad and LBD samples as well as modern biomes in South Africa[39]. The fraction of C_4 vegetation, f_{C4}[98], for C_{31} calculated from modern African vegetation endmembers[99] is plotted on the top x axis.

-"In contrast, the Pleistocene samples are not within this range, except for lower Unit M. The ACL27-33, Norm31 and Norm33 indices all indicate a very different range for Units N to E, compared to the Holocene (Figure S.18). This suggests non-analog environments for most parts of the Pleistocene with a different vegetation and/or rainfall structure than today" This needs strengthening. A difference between Pleistocene and Holocene (or between Pleistocene and any single modern biome) does not automatically imply non-analog environments in the Pleistocene. Presumably, a non-analog environment would include combinations of d13C and dD values (in parallel w/ other paleoenvironmental data) unlike any observed in southern Africa today, hinting at a unique combination of climate and vegetation. This may indeed be the case, but I'm not yet convinced. Can you demonstrate this through a more detailed comparison of Pleistocene to present-day southern Africa? This may entail further analysis of the isotopic data, perhaps in parallel with consideration with associated lines of paleoenvironmental evidence (e.g., pollen, fauna). Also, non-analog in what way? Can you clarify how the environments were unlike anything found in the region today?

Our new Figure 5 and the revised text show and discuss this, as we included values of modern biomes from the transect by Herrmann et al. (2016, 2017) in the Figure.

"This suggests that at least the lower units around Unit O during Middle Pleistocene occupation at Florisbad have, in addition to C4 grasses, a substantial C3 plant component, probably fynbos-like and karroid vegetation associated with cooler climates, of which only the latter is prominent in the early Holocene layers. This combination of vegetation indicates a non-analog environment that different seasonal rainfall patterns would drive compared to today." Can you elaborate on how seasonal changes in rainfall would drive this? Also, some of the fynbos-associated taxa (e.g., Ericaceae and Restionaceae) do occur occasionally in the nearby region (Chevalier et al 2021)—so are we sure that their presence alongside C4 grasses is non-analog? I'm not arguing that the Pleistocene environment was analogous to today, but I do need more convincing that it was not.

This section was rephrased and a new refernce for precession (Berger and Loutre 1991) was added.

Human adaptation to non-analog environments: The authors propose that environmental changes demonstrated at Florisbad account for changes in the MSA lithic assemblages at the site:
"The assemblage is dominated by hornfels, with elongated blanks and a relatively high frequency of retouch, including scrapers as the dominant formal tool type. These features point to a more curated technological strategy, where transported blanks were shaped and maintained on site, consistent with a technological emphasis on longer tool use-lives and mobility strategies that involved carrying and conserving raw material. This technological

behaviour aligns with the spring vent associated faunal spectrums and our vegetation reconstruction, suggesting a landscape where wetlands and possibly higher numbers of browsing fauna structured human activities. In this context, curation and transport of tools may have been adaptive to a more spatially constrained and patchy ecosystem."

What is the link between patchy ecosystems and the observed technological strategies? Robust links between environment and behavior demand some sort of theoretical framework linking one to the other. There is a large, theoretically-informed literature establishing links between environment and lithic technology, none of which is considered here. I encourage the authors to engage with this literature to strengthen the proposed link between environment and lithic technology.

Similar questions apply to the subsequent paragraph, where the authors argue that "In such an ecosystem, reliable and predictable access to resources appears to have supported shorter tool use-lives, reduced curation and opportunistic on-site tool manufacture." This reads to me as a post hoc interpretation. What is the link between "reliable and predictable access to resources" and the observed technologies? The authors need to engage with the relevant literature to make a stronger argument here.

We agree with the reviewers that this point was not developed strongly from a theoretical point of view. We have now completely re-written this section and added the following new references:

Broughton, J. M. *Resource Depression and Intensification during the Late Holocene, San Francisco Bay: Evidence from the Emeryville Shellmound Vertebrate Fauna*. (University of Washington, 1995).

Cannon, M. D. A model of central place forager prey choice and an application to faunal remains from the Mimbres Valley, New Mexico. *Journal of Anthropological Archaeology* **22**, 1–25 (2003).

Munro, N. D. Zooarchaeological Measures of Hunting Pressure and Occupation Intensity in the Natufian: Implications for Agricultural Origins. *Current Anthropology* **45**, S5–S34 (2004).

Torrence, R. Time budgeting and hunter-gatherer technology. *Hunter-gatherer economy in prehistory* 11–22 (1983).

Torrence, R. *Time, Energy, and Stone Tools*. (Cambridge University Press, 1989).

Bousman, C. B. Hunter-gatherer adaptations, economic risk and tool design. *Lithic technology* **18**, 59–86 (1993).

Ambrose, S. H. & Lorenz, K. G. Social and ecological models for the Middle Stone Age in southern Africa. in *The emergence of modern humans: An archaeological perspective* 3–33 (1990).

Mackay, A., Stewart, B. A. & Chase, B. M. Coalescence and fragmentation in the late Pleistocene archaeology of southernmost Africa. *Journal of human evolution* **72**, 26–51 (2014).

Kuhn, S. L. *Mousterian Lithic Technology: An Ecological Perspective*. (Princeton University Press, 1995). doi:10.1515/9781400864034.

Kuhn, S. L. Neanderthal technoeconomics: an assessment and suggestions for future developments. *Neanderthal lifeways, subsistence and technology: one hundred fifty years of Neanderthal study* 99–110 (2011).

Morrow, T. A. Bigger is better: comments on Kuhn's formal approach to mobile tool kits. *American Antiquity* **61**, 581–590 (1996).

Braun, D. R. & Harris, J. W. Technological developments in the oldowan of koobi fora: innovatives techniques of artifact analysis. *Treballs d'Arqueologia* 117–144 (2003).

Henderson, Z. L. Spatial patterning at southern African middle Pleistocene open-air sites: Florisbad, Duinefontein 2/2 and Mwanganda's Village. *Ph.D. Dissertation. University of Cambridge* (2001).

Other edits, questions, and suggestions:
Abstract: "We observe contrasting, non-analogous environments during the Middle and Late Pleistocene." Non-analog in what way? Can you be more specific?

This was rephrased.

Abstract: "It also provides the foundation to evaluate their cultural adaptation strategies through time." How so? Can you be more specific?

Introduction: insert comma "…seems contradictory at some levels[comma here] with interpretations requiring different taphonomic histories for vegetation reconstruction"
Comma inserted.

Results: "In contrast, in the Pleistocene samples, C33 is the dominant n-alkane with C31 the second most abundant n-alkane" As you did for the preceding sentence (i.e., "indicating a mainly terrestrial plan wax source"), can you briefly indicate what this means?

Added: indicating a strong presence of C_4 plants and/or grasses.

Results: "However, no LBD sample shows the disproportional high C33" use subscript for 33
Corrected.

Results: in the text (e.g., "However, while 3TP1 ranges from -28.1‰ (C25) to −23.8‰ (C33), FLB24 shows a different trend in which homologue is highest, ranging from -25.4‰ (C25) to −28.3‰ (C29)" and figures (e.g., Figure 3), would it be possible to also provide calculations for percent C4 biomass? This would facilitate interpretation of the data. Polissar et al. (2019) give an example of how to do this.

We did the calculation and included it in the new Figure 5. We added to the Methods section to include the calculation method.

Discussion, The Florisbad Sequence: "At both sites, the modern samples are closest to the modern Grassland values…" Modern samples of what?

We have replaced "modern" with "recent" and included the list of sample names in brackets.

Discussion, The Florisbad sequence: "Further hints at fynbos-type contributions are the Norm33 index values for sample FLB-LD24." How so? Please clarify.

We added a reference to the new Figure 5 and rephrased the sentence. Further hints at fynbos type contributions are the Norm33 index values for sample FLB-LD24, which fall within the range of modern fynbos biome Norm33 values.

Discussion, The Florisbad sequence: "This could suggest interglacial climatic conditions with strong evaporation and associated vegetation comparable to today." Important to keep in mind that precipitation changes may not solely follow glacial-interglacial cycles. In southeastern Africa, evidence from lacustrine sediments from Tswaing Crater and the marine core CD154 10–06 P Fe/K record both indicate a strong precessional signal (Chase 2021)
We agree that precipitation changes in South Africa are more complex than glacial-interglacial cycles. We added a rephrased paragraph and prefer to cite *Partridge, T.C., Demenocal, P.B., Lorentz, S.A., Paiker, M.J., Vogel, J.C., 1997. Orbital forcing of climate over South Africa: a 200 000-year rainfall record from the Pretoria Saltpan. Quaternary Science Reviews 16, 1–9*, rather than Chase 2021 as a primary reference for the interior of SA.

Discussion, The Florisbad sequence: "The faunal isotopes also show lower δ13C values in the enamel of grazers than comparable species today indicating more C3 plant presence [15]." But note that most grazer d13C values do not vary in relation to the availability of woody cover (Robinson et al 2021). Might this indicate consumption of C3 grasses?

We have not been specific enough here. We assumed this indicates presence and consumption of C_3 grasses by grazing species (as suggested in the referenced publication) rather than only (or additional) woody plant cover. We have added a sentence to clarify.

Discussion, The Florisbad sequence: "The fauna in Unit F shows only one mixed feeder (a steenbok) and no browsers or other mixed-feeders such as eland and modern springbok" Provide genus and species We added genus and species.

Discussion, The Florisbad sequence: "Explanation could be an under-representation of C4 grasses in our samples or it could be that A. bondi was highly selective of C4 grass even in a shrubby vegetation. [65] suggested a scenario of year-round production of grass, which implies weak rainfall seasonality, similar to a year-round rainfall zone." There is also broader evidence that loss of grassland species across the Pleistocene-Holocene transition was not associated with declining availability of grasses on the landscape (Norwood et al 2025), suggesting that what matters here is not extent of grassy vegetation but instead its seasonal availability. And wouldn't reduced seasonality, which means some rainfall in the winter, perhaps lead to presence of C3 grasses? This might explain the low d13C values of some fossil grazers from Florisbad (Codron et al. 2008)

We agree that a presence of C_3 grasses is the likely explanation of low $\delta^{13}C$ values in fossil grazers at Florisbad, with *A. bondi* being one with the highest $\delta^{13}C$ values (Codron et al. 2008). We have rephrased this part of the discussion and added the Norwood et al. 2025 citation.

Discussion, Age of the Florisbad Cranium: “Our ages provide a chronological range for the human remains, ranging from 212 ka to 130 ka, considering the error at 1 σ, significantly younger than the age obtained on the human remains by[19]” But considering the two-sigma overlap of previous ages and yours, perhaps they are not inconsistent.

We rephrased this paragraph to include the age overlap at 2σ.

References

Chase B. 2021. Orbital forcing in southern Africa: Towards a conceptual model for predicting deep time environmental change from an incomplete proxy record. Quaternary Science Reviews 265
Codron, D., Brink, J. S., Rossouw, L., & Clauss, M. (2008). The evolution of ecological specialization in southern African ungulates: competition-or physical environmental turnover?. Oikos, 117(3), 344-353.
Chevalier M, Chase BM, Quick LJ, Scott L. 2021. An atlas of southern African pollen types and their climatic affinities. In Quaternary Vegetation Dynamics: The African Pollen Database, ed. J Runge, WD Gosling, A-M Lézine, L Scott, pp. 239-58. Leiden: CRC Press
Norwood A, Rowan J, Faith J. 2025. Reconciling the environmental implications of late Quaternary faunal and pollen records in southern Africa. Quaternary Science Advances 17
Polissar PJ, Rose C, Uno KT, Phelps SR, deMenocal P. 2019. Synchronous rise of African C4 ecosystems 10 million years ago in the absence of aridification. Nature Geoscience 12: 657-60
Robinson J, Rowan J, Barr W, Sponheimer M. 2021. Intrataxonomic trends in herbivore enamel δ13C are decoupled from ecosystem woody cover. Nature Ecology & Evolution 5: 995-+

Reviewer #3 (Remarks to the Author):

This paper seeks to reconstruct the environments represented through the sediment sequence at the South African archaeological site of Florisbad using a variety of proxy data, but focussing on plant wax n-alkanes. In addition, new luminescence dating is conducted at the site. The paper concludes that the Homo sapiens inhabitants of the site lived in non-analogue environments, and that the well-known cranium from the site dates to ~200 ka i.e. significantly younger than published age estimates. The results presented in this paper are important, and should be of considerable interest to readers of Communications Earth and Environment. However, I believe that a major re-write of the luminescence dating sections, is required prior to publication. It should be noted that while I find the explanation of the leaf wax data convincing, I am not a specialist in this field.

Luminescence dating

My main concern with this paper is that the luminescence dating work presented in a rather disorganised manner, and some of the assumptions/decisions made have not been properly justified or explained. Although individually rather picky, collectively the concerns outlined below limit my ability to interpret the luminescence data, and therefore my confidence in the ages presented.

First paragraph of "Luminescence dating". It is stated that FLB3 was measured using single-grain discs, but it is not clear whether the sample was measured as single grains. Later in the paper it is stated that the TT-OSL was measured as "micro-aliquots" i.e. more than one grain in each 300 µm sample position, but the OSL signal was measured on single-grains. Given that all of the grains measured for each samples were the same size, does this mean that smaller aperture single-grain discs were used, or is this signal also micro-aliquot? This question is only answered much later in the paper in the "methods" section. Also, the "age results" are said to be computed by the central age model (CAM), but this must in fact be the equivalent dose.

We followed the policy of the journal and placed the Methods section at the end, where one can find details about the methodology. However, in the caption of Table 3 presenting the ages, we specified how the equivalent doses (D_e) were measured: on fine grains (4-11 µm) for FLB1 and FLB2, on 100-140 µm grains for FLB3 and on 100-120 µm grains for LBD4. In the protocol column of the same table, we detailed how were the grains analysed: as multigrain (MG), single-grain (SG) and micro-aliquots (MA). More specifically, for multigrain (MG) aliquots, the fine grains of FLB1 and 2 cover the whole disc; for FLB3, 1 mm Ø aliquot were measured. For the single-grain (SG) OSL analyses of FLB3, one grain per hole was measured in 150 µm-hole discs; for the micro-aliquots (MA) TT-OSL analyses of FLB3, 2-3 grains per hole were measured in 300 µm-hole discs in order to increase the yield. We added this information to the caption of Table 3 to avoid repetitions between the Results and Methods sections.

Second paragraph of "Luminescence dating". The dose recovery data are described poorly, and this is not helped by the presentation of the data in Figures S11-S13. The authors define the dose recovery ratio, and then express the results as percentages. This is both unconventional and unhelpful. Figures S11 and S12 are also unconventional – why present data for individual aliquots when the mean (or CAM) dose recovery ratio (by sample and signal) is more informative? All of the mean/CAM data could be displayed in one figure. Data for sample FLB3 are missing from Fig. S12. The units for the radial axes of figure S13 is wrong – you are plotting dose recovery ration not De. For all radial plats, replace the last sentence of the caption with something more specific such as "Plots were created using the plot_RadialPlot() function in the R package Luminescence. Lastly, there is a literature on the overdispersion of data from quartz samples, which supports the assertion that 28% is (just) consistent with a well-bleached sample. However, it tends to implicate beta-microdosimetry not "intrinsic variations" of luminescence characteristics as suggested here.

We replaced Figs. S11-S13 with one figure where the mean recovered/given dose ratios are presented (numbered Fig. S14). We changed the caption of all radial plots as suggested. We added a reference regarding the 28% of overdispersion consistent with well-bleached samples in the text. The calculated DRT value corresponded to the mean recovered value/given value

± the standard deviation in %. We corrected the text presenting mean recovered value/given value ± the standard deviation.

In the "luminescence dating" section the authors state that they give "more credibility" to the quartz OSL ages, which is defensible, but they then sometimes discuss the pIRIR ages later in the paper, only to dismiss them. It would be tidier to make a case for only believing the OSL ages somewhere in this section, and then not referring to the other age estimates later.

Corrected.

The authors make a strong case for reassessing the age of the Florisbad cranium. However, the authors reject their own TT-OSL data on the basis that the dose recovery results are poor and the TT-OSL ages are older than those produced by conventional OSL. They then cite three TT-OSL ages from a doctoral thesis (Pinder, 2021), two of which are very similar to their conventional OSL ages. Why did Pinder get the "correct" ages using TT-OSL, whereas their TT-OSL dates are interpreted as overestimates for the same sedimentary units? As a minimum, this anomaly should be acknowledged, and ideally some attempt to explain it should be provided.

We did not use the exact same TT-OSL protocol as Pinder (2021). They used the protocol of Arnold et al. (2015), whereas we applied the one proposed by Porat et al. (2009). We commented on this aspect in the main text.

Arnold, Lee J., Martina Demuro, Josep María Parés, Alfredo Pérez-González, Juan Luis Arsuaga, José María Bermúdez de Castro, and Eudald Carbonell. 2015. 'Evaluating the suitability of extended-range luminescence dating techniques over early and Middle Pleistocene timescales: Published datasets and case studies from Atapuerca, Spain', Quaternary International, 389: 167-90.

Porat, N., G. A. T. Duller, H. M. Roberts, and A. G. Wintle. 2009. 'A simplified SAR protocol for TT-OSL', Radiation Measurements, 44: 538-42.

Lastly, and most importantly, the authors need to provide much stronger evidence that their quartz OSL ages finite and not minimum ages. All three of the Florisbad OSL samples yielded high equivalent dose values (366, 303 and 185 Gy in age order), at least two of which could easily be "saturated" i.e. beyond the point at which the luminescence signal ceases to grow predictably with increasing dose. There is a considerable literature on this topic, but many studies (e.g. Chapot et al., 2012, Radiation Measurements) suggest that as the OSL signal nears saturation, age underestimates occur. This is because the laboratory induced luminescence is larger than that observed for the same dose administered in nature. The saturation limit is sample dependant, but Chapot observed this effect above ~150 Gy, so it could easily be present in the OSL dates presented in this paper. Successful dose recovery does not test for this phenomenon, since all doses are administered in the laboratory. The authors themselves make reference to problems associated with quartz saturation in a previous study at Florisbad (Grun et al., 1996) and in relation to one of their own samples, FLB3, which was stated to be "close to saturation". Interestingly, FLB3 has the lowest equivalent dose (185 Gy) of the three Florisbad samples, so it is not clear why the other

samples should not be closer to saturation. Also, the authors state that they performed single-grain analysis on sample FLB3 because of its closeness to saturation, but don't explain the logic of this decision. It is probably a defensible decision, but the reader needs to know their reasoning. Given that the authors wish to revise the accepted age of the Florisbad cranium downwards (i.e. make it younger) on the basis of quartz OSL dating, that quartz OSL dating is known to yield age underestimates as samples approach saturation, the authors need to convince readers that the new younger age is not the result of undiagnosed saturation. I suggest that the following revisions are made:
1) Show growth curves for all three of the Florisbad samples. This is easy and the data already exist. The curve fitting parameter D0 which governs the rate of saturation for a saturating exponential curve should also be stated.

We provide below a figure showing the growth curves obtained on natural and artificially irradiated fine grains samples FLB1 and 2, and on a natural 100-140 µm grain size multigrain aliquot and single grain of FLB3. We can see that for a similar dose, FLB3 measured as multigrain aliquot is close to saturation (E = 230 ± 23 Gy), while with single grain measurement, we are able to screen the single grain data and select unsaturated grains (e.g., F = 275 ± 21 Gy). We started our OSL measurements on multigrain aliquots for all samples, but considering the growth curves of FLB3, we decided to measure single grains to be able to reject saturated grains. Despite having a CAM D_e lower than the other samples, FLB3 shows a saturation of the signal measured on multigrain aliquots at ca. 200 Gy. This difference may be explained by the grain size used for measurements: 4-11 µm for the peat layers (FLB1 and FLB2) and 100-140 µm for the sand layer (FLB3). Moreover, performing single grain measurements on FLB3 provided information on the grain population (see Table R1 below). Note that we initially selected the individual D_e that are < $2D_0$ (Wintle and Murray 2006) in the previous version of the manuscript. We reviewed our single grain age for FLB3 following your recommendation (see next comment), added this information in the description of the results and both figure and table below in the supplementary material.

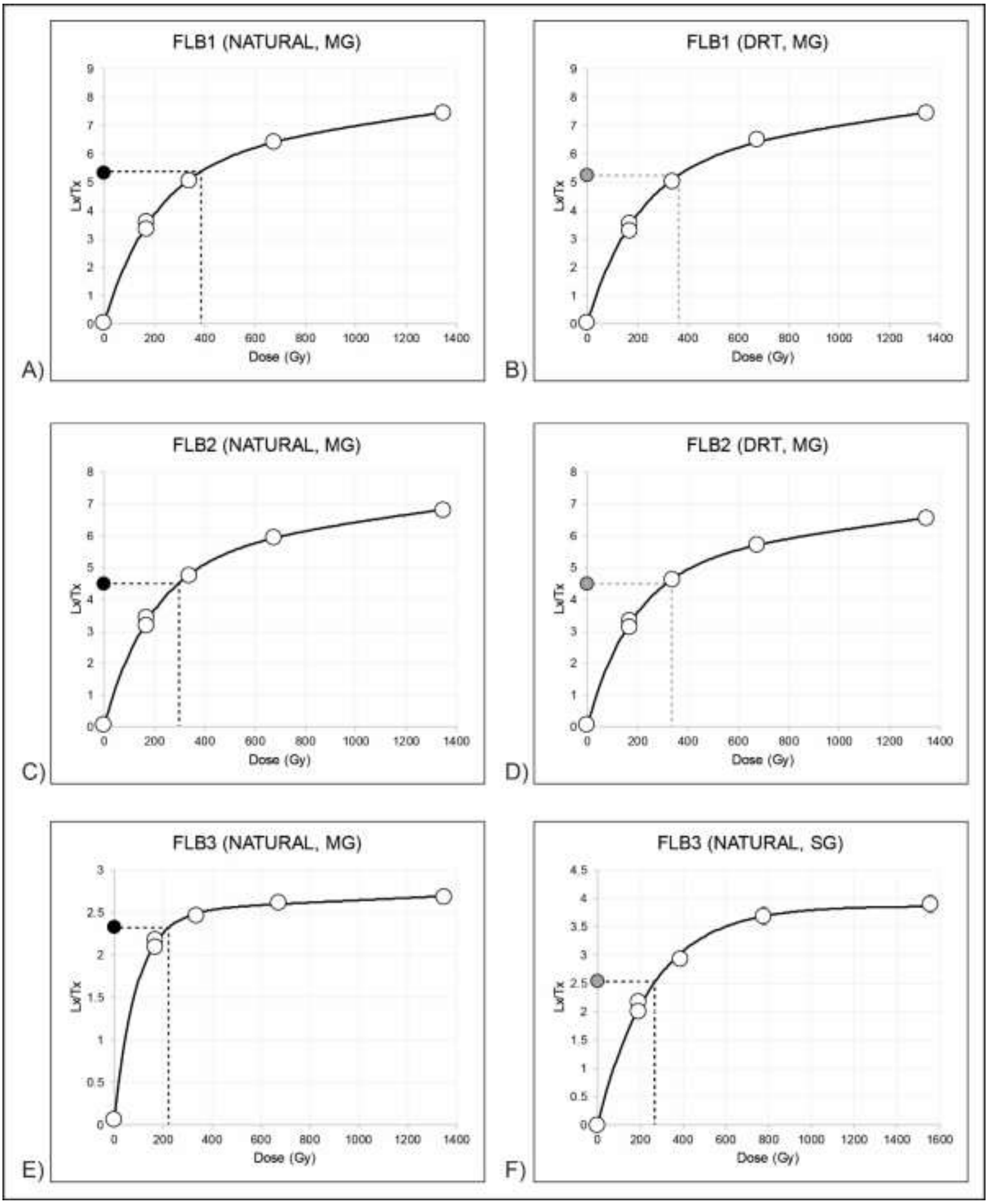

Fig. R1. Dose response curves (DRC) obtained for FLB1 and FLB2 (4-11 µm), and FLB3 (100-140 µm) on a natural multigrain aliquot (A = 380 ± 22 Gy; C = 303 ± 15 Gy; E = 226 ± 20 Gy), on a natural single grain of FLB3 (F = 275 ± 21 Gy), and on an irradiated aliquot of FLB1 and FLB2 (B: given dose = 364 Gy; recovered dose = 364 ± 19 Gy; D: given dose = 309 Gy; recovered dose = 322 ± 16 Gy). D_0 for natural samples are 206 ± 20 Gy (A), 193 ± 18 (C), 96 ± 10 Gy (E) and 254 ± 18 (F).

FLB3	
Number of grains measured[1]	1500
Number of grains that passed criteria[2]	309
Number of rejected grains[3]	250
Accepted grains[4]	59

Table R1. Statistics on the selected single grains for FLB3. [1]Total number of individual quartz grains measured using the SAR protocol; [2]Number of grains that provided a luminescence signal passing the following criteria: recycling ratio ≠ 1 ± 10%; recuperation < 5%; error on the test dose <10%; T_n signal > 3σ above background; [3]Among the grains that passed criteria, those for which the D_0 was ≥ to 150 Gy (following Guo et al., 2017). [4]Number of grains used to calculate the equivalent dose using the CAM.

2) Calculate the effect of Do on De by rejecting quartz grains at progressively higher D0 values for your single-grain OSL dataset. A plateau in equivalent dose has been interpreted as indicating the correct value for age determination e.g Guo et al. (2017) Journal of Quaternary Science 32 and Thomsen et al. (2016) Quaternary Geochronology 31. The logic here is that De doesn't change above a D0 value at which saturation does not cause dose underestimates.

In the first version of our manuscript, we did reject saturated grains following the recommendation of Wintle and Murray (2006), i.e., taking into account $D_e < 2 \times D_O$. We reviewed our single grain age for FLB3 following Guo et al. (2017): for the DRT results, we sorted the accepted grains according to their D_0 values and recalculate the recovered dose while increasing the D_0 threshold (Fig. R2). A plateau at a value close to 1 is reached from D_0 ≥ 140 Gy. Starting from this value, the number of saturated grains in our dataset reached 0. Proceeding the same way with our single grain D_e values, we identified the "optimum D_0 threshold" (i.e., the minimum value to reach to avoid saturated grains). For FLB3, starting from D_0 = 150 Gy, the number of saturated grains reached 0, hence using this value as D_0 threshold for our dataset (Fig. R3). The new CAM D_e for FLB3 is higher, of 214 ± 9 Gy, with an overdispersion of 29 ± 3%. The age is 168 ± 14 ka. We corrected the text accordingly.

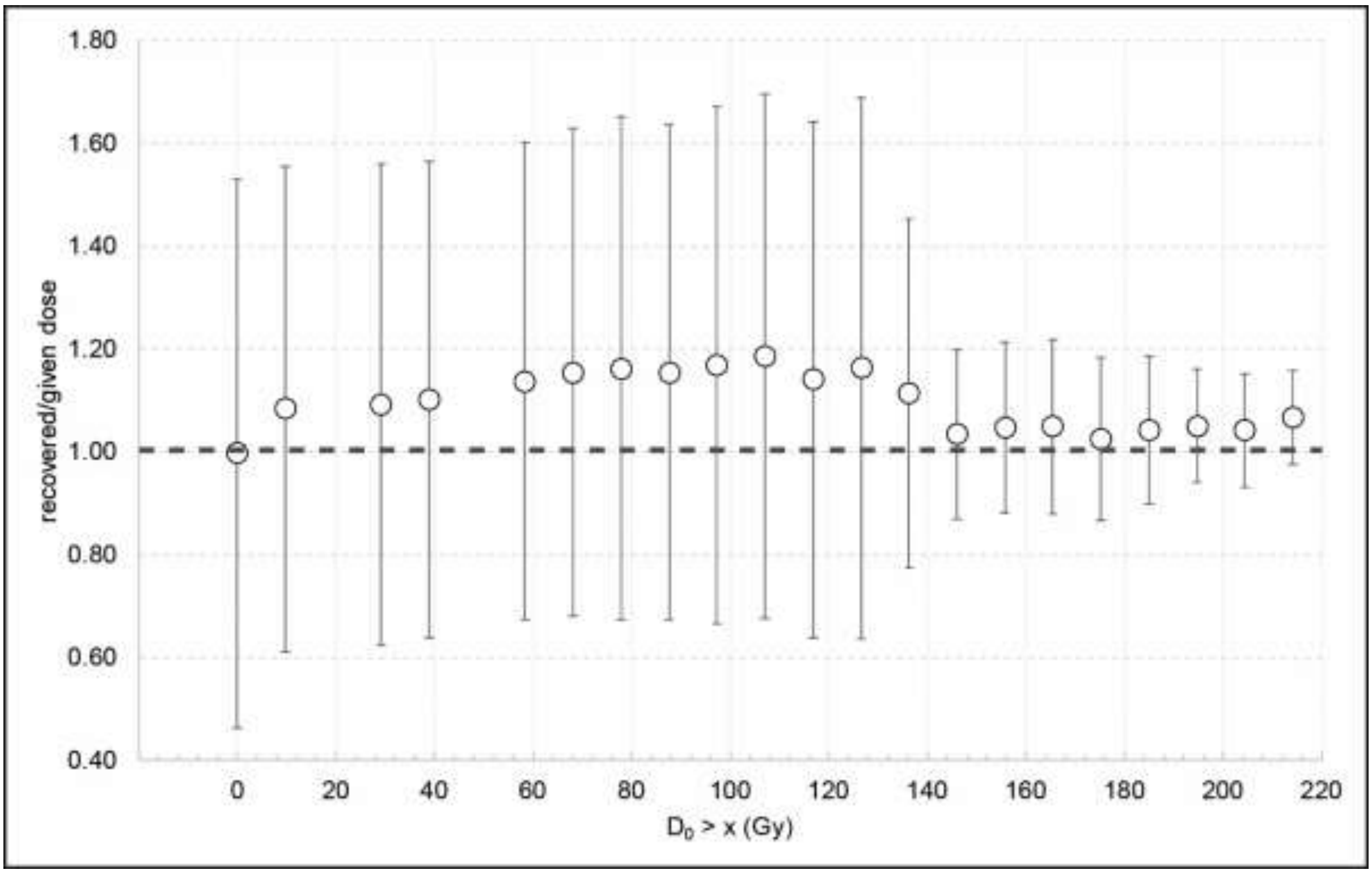

Fig. R2. DRT values sorted as a function of the D_0 threshold. The recovered/given dose ratio was calculated increasing the minimum D_0 threshold, from 0 to 214 Gy. The grey dashed line indicates the recovered/given dose value of 1.

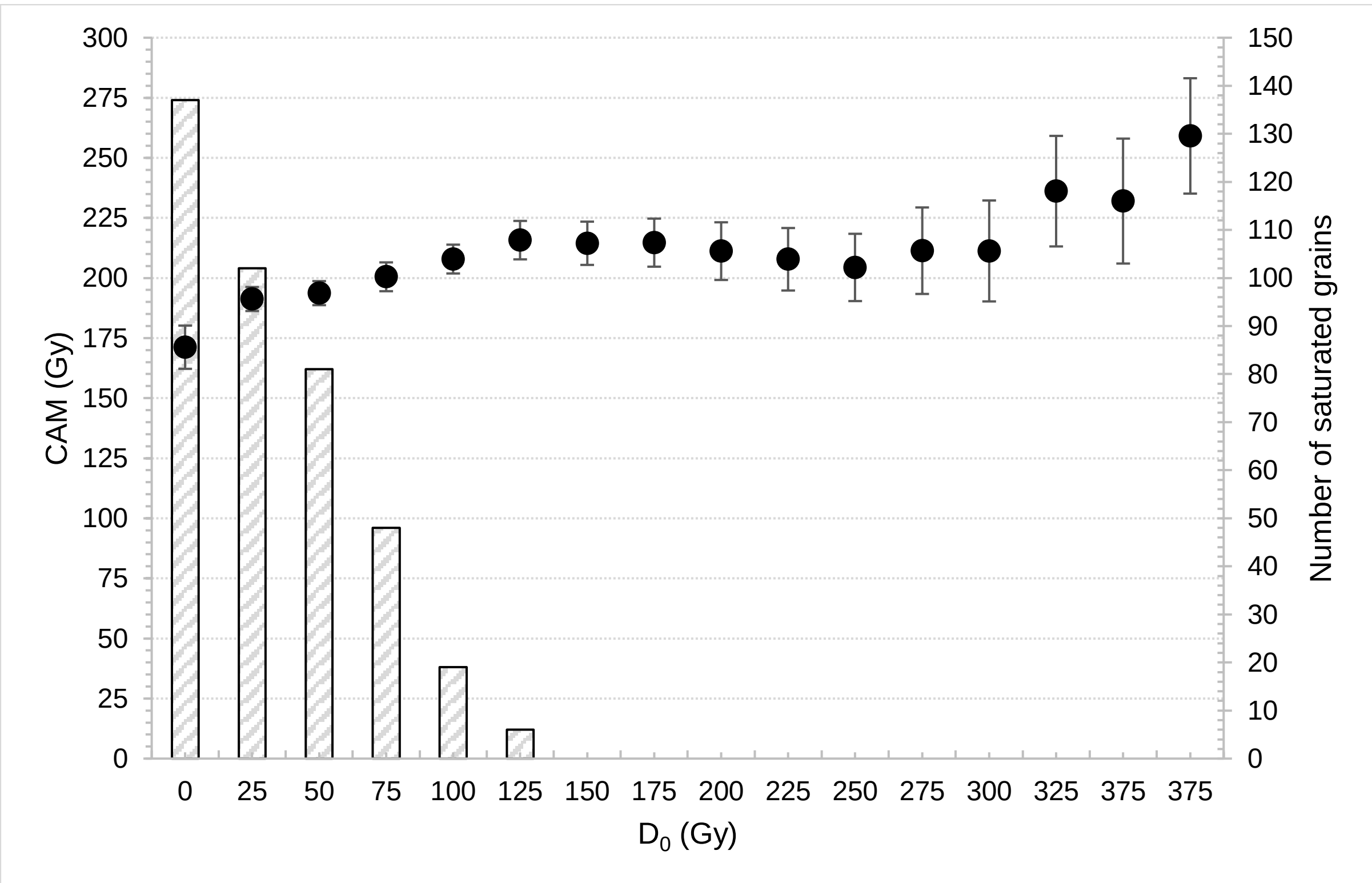

Fig. R3. Number of saturated grains (i.e., grains that passed the selection criteria but for which no intersection of the L_N/T_N signal on the growth curve was reached), and CAM values

calculated increasing the D_0 threshold from 0 to 375 Gy. Starting from a value of 150 Gy, no saturated grains are observed.

3) Measure lower temperature pIRIR signals for the 4-11 µm samples. The authors believe at least two of their pIRIR290 samples to contain unbleached residual doses which cause age overestimates. Since lower temperature pIRIR signals have higher bleaching rates, a well-bleached signal could be found at lower temperatures e.g. pIRIR225. Unfortunately, since fading rates also increase with decreasing temperatures, the authors would need to demonstrate that they weren't simply lowering the measurement temperature until some combination of fading and bleaching gave the "right" ages. This could be done by measuring g-values (fading rates) for each signal. Also, sample FLB1 could be used as a control, since it yielded a pIRIR290 age similar to that for the OSL measurements, and is therefore presumably well-bleached.
The data already exist to implement suggestions 1 and 2, which I regard as essential. The third suggestion requires a lot of additional work, but would considerably strengthen the case for revising the age of the Florisbad cranium.

We conducted $pIRIR_{225}$ measurements fort the three samples, as well as fading measurements (see details of the procedure in the Methods section and results in Table 3 and SOM Figure S13). The $pIRIR_{225}$ faded ages are in agreement with the OSL ages for both FLB1 and FLB2, and appear overestimated for FLB3. Only the $pIRIR_{225}$ corrected age of FLB1 falls within the same range of the OSL age. We discuss these issues in the Results section.

General points
1) The paper appears to have been written with Harvard style references and then converted to the numerical reference style. This produces awkward text such as a sentence in the "human adaptation to non-analog environments" section which starts "[65] suggested a scenario of year-round production of grass…". I suggest that the authors re-write sentences where the name of the reference is an integral part of the sentence.

This is correct, and we have re-written all sentences accordingly.
2) The style of the paper is occasionally rather colloquial.

Large parts of the paper have been re-written and the whole paper has been proof-read again with minor changes. We hope this improved the style.
3) Radiocarbon ages are not presented in a consistent style, with mean values and both 1 and 2 sigma ranges appearing at various places in the text/figures.

This is our mistake and indeed confusing. We have adjusted the text, figures and tables to show conistsently the uncalibrated dates from Scott and Nyakale 2002. However, we give ranges for units where there are several radiocarbon dates and the date with ± for Unit D where there is only one date.

Response to reviewers

We thank the reviewer for their time and the insightful and helpful comment. We have made all requested changes. Our detailed response is below in blue.

Reviewer #1 (Remarks to the Author):

Thank you for the opportunity to examine the revised version of this manuscript. The authors have made a strong effort to address our feedback, and I believe the result is a stronger paper. In particular, the revisions have helped make a stronger case for the non-analog nature of these Pleistocene ecosystems, and strengthened the interpretation of the archaeological data. I see that one of the previous reviewers was largely concerned with the luminescence ages. The authors have responded to those concerns, but I lack the expertise to assess these revisions.

I have only one (fairly minor) substantive comment with the revisions. The authors suggest that the evidence for intensive marrow extraction and carcass processing in Unit F does not align with expectations for highly productive environments. Bone marrow is key source of fat (especially on wild ungulates that are relatively lean), and ethnographic observations consistently show that hunter-gatherers regularly exploit marrow from long bones. In other words, within-bone nutrients (e.g., marrow) should be relatively highly ranked relative to other portions of an ungulate carcass, so I wouldn't necessarily interpret evidence for long-bone marrow extraction as a sign of intensification or resource stress. In contrast, evidence for intensive processing of body parts with minimal amounts of marrow (e.g., phalanges) or labor-intensive grease extraction might be indicative of intensification. More broadly, zooarchaeological indicators of intensification are perhaps most effectively used when examining trends through space or time--e.g., where one might observe temporal shifts in exploitation of high versus low ranked prey, shifts in prey age structure, and evidence for changes in carcass processing strategies. But it may be harder to interpret a single assemblage like Unit F as a sign of good or bad times (relative to what?)

I encourage the authors to potentially reconsider this point, but otherwise I think this is a solid paper that adds considerably to our understanding of an important site.

We rephrased/deleted bone marrow processing intensity from relevant sentences in the discussion. There is one complete paragraph on it, which was deleted. We agree with the reviewer comment, but at the moment the state of the assemblage does not allow us to make a strong claim either way.

And a few minor edits. Apologies if I missed these in the first round of review.
Line 29: "Such an environment" should be "Such environments" corrected
Line 389: spell genus name in full when it begins a sentence: "A. bondi" to "Antidorcas bondi" corrected
Line 469: genus + species needed for steenbok this was added

We have made the changes requested by the editor:

We list our remaining requests below:
1) Please be more cautious about interpreting long-bone marrow extraction in Unit F as evidence of intensification or resource stress. Please see our answer to the reviewer comment

above.
2) Please ensure that the revised chronology is fully consistent throughout the manuscript, including the main text, figures, tables and related discussion. In particular, if the FLB3 age has been updated to 168 ± 14 ka following the D0-threshold analysis, please make sure that all corresponding values, including the Unit M age and the derived age range for the cranium, are updated consistently wherever they appear.

We checked all instances and confirmed the FLB3 age is correct at all mentions.

Additional changes include:

- A schematic image of the vent was added to Figure 6

- Table 1 was moved to the supplementary material as new Table S2 to fulfil the journal formatting requirements for long tables. All Tables and their mention in the main manuscript and the supplementary were re-numbered accordingly.

- Table 2 was split into the new Table 1 and Table 2 S2 to fulfil the journal formatting requirements for long tables. All Tables and their mention in the main manuscript and the supplementary were re-numbered accordingly.

- Figure S11 was corrected and replaced

- The stratigraphy of Unit F-L was explained in greater detail in the supplementary.

- Unit E was changed from "Late MSA" to "MSA" in Table S2 and Figure 3 as it is not clear if the lithic fall for certain within a Late MSA technocomplex (MIS 4 or younger).

- The error ranges for OSL were added in Table S2.

- very few small changes in spelling and grammar were done in the manuscript and supplementary information
