# Supplementary material for: Non-analog environments supported early Homo sapiens in southern Africa 200 000 years ago
Source: Commun Earth Environ. 2026 Oct 30;7(1):829. doi: 10.1038/s43247-026-04116-4 (PMC13722590; doi:10.1038/s43247-026-04116-4)
Supplement: Supplementary file 2 [file 43247_2026_4116_MOESM2_ESM.pdf]

Supplementary Information for

Non-analog environments supported early *Homo sapiens* in southern Africa 200 000 years ago

Michaela Ecker[1,2*], Maïlys Richard[3,4], Nils Andersen[5], Beatrice Bin[3,6], Will Archer[7,8,9,10], C. Britt Bousman[11], Josep M. Parés[6], Andri C. van Aardt[12], Louis Scott[12], Michael B. Toffolo[3,6,12*]

1 Institute of Prehistoric and Protohistoric Archaeology, Kiel University, Kiel, Germany;

2 Archaeology Department, McGregor Museum, Kimberley, South Africa

3 Archéosciences Bordeaux, UMR 6034 CNRS-Bordeaux Montaigne University, Pessac, France

4 Department of Early Prehistory and Quaternary Ecology, University of Tübingen, Tübingen, Germany

5 Leibniz Laboratory for Radiometric Dating and Isotope Research, Kiel University, Kiel, Germany

6 Geochronology and Geology Programme, Centro Nacional de Investigación sobre la Evolución Humana (CENIEH), Burgos, Spain

7 Max Planck Partner Group, Department of Archaeology and Anthropology, National Museum, Bloemfontein, South Africa;

8 Florisbad Quaternary Research Station, National Museum, Bloemfontein, South Africa;

9 Department of Geology, University of the Free State, Bloemfontein, South Africa;

10 Department of Anthropology, The George Washington University, Washington DC, USA

11 Center for Archaeological Studies, Department of Anthropology, Texas State University, San Marcos, Texas, USA

12 Department of Plant Sciences, University of the Free State, Bloemfontein, South Africa

*corresponding authors: M. Ecker (mecker@ufg.uni-kiel.de) and M. Toffolo (michael.toffolo@u-bordeaux-montaigne.fr)

Content:

Supplementary text:

SI 1: Florisbad and Lombard's Drift

SI 2: Pollen and charcoal analysis at Lombard's Drift

SI 3: Biomarkers – extended results and discussion

Supplementary figures: Figure S1 to S20

Supplementary tables: Table S1 to S11

References

SI 1 - Florisbad and Lombard's Drift

Florisbad (28°46'4.93"S; 26°4'10.42"E) is located 41 km to the northwest of Bloemfontein and 1.5 km to the south of a large seasonal lake called Soutpan[1,2]. The site is the type locality of the Florisian Land Mammal Age, based on a large Middle Pleistocene faunal assemblage[3–7], and has produced multiple human occupational contexts associated with Middle Stone Age (MSA) and Later Stone Age (LSA) lithic industries[8–12]. The most important find unearthed at Florisbad is the partial cranium of an early *Homo sapiens* (previously known as *H. helmei*) discovered in 1932 by Thomas Frederick Dreyer, which was dated to 259 ± 35 ka by applying electron spin resonance to an upper right third molar recovered with the fragments of the cranium[13–18]. Other notable materials, not commonly found at open-air sites of this age, include a wooden implement[19] and oribatid mites[20].

Florisbad is a spring mound featuring a thermal spring that feeds into an adjacent wetland rich in reeds (*Phragmites australis*) and sedges (*Juncus rigidus*, *Schoenoplectus triqueter*)[21,22]. The site is characterised by a complex excavation history that spans more than one hundred years[23]. Since the 1910s, a Main Excavation area has been excavated into the leeward side of the spring mound, where the human cranium was found, and expanded in the 1950s, 1980s, and 1990s, reaching bedrock at several locations. Additional test trenches were excavated to bedrock in the 1980s near the top of the mound (Test Pit 3) and at its edge, close to the wetland (Test Pit 1 and Test Pit 2)[2,24]. The Main Excavation trench is today protected by concrete bricks, although a few standing sections are available for sampling, notably the Dreyer Section, which features the entire stratigraphic sequence (except for the last 2000 years).

The stratigraphic sequence of the mound comprises alternating layers of peat (organic sediments) and sand that accumulated under different environmental conditions in the past ~280,000 years, as indicated by a coring programme and following luminescence and electron spin resonance dating[10,15,25–27]. These layers are organised into sedimentary units according to different but complementary nomenclature systems. The sedimentary layers in the Main Excavation area were designated by Kuman & Clarke[1] and Kuman et al.[10] from younger to older in alphabetical order from B to P based on their composition, whereas Toffolo et al.[24] named the sedimentary units of Test Pit 2 and 3 from top to bottom using progressive numbers from 1 to 10, based on their composition and formation processes. The two systems have been linked[24] based on micromorphological analysis, absolute dating, and archaeological associations (Table S1). In several places and at various stages of the Middle and Late Pleistocene, these layers were intruded by spring vents that issued water to the surface and thus represent a displacement of the spring eye over time. These vertical channels, now filled with sand, 'sampled' the existing surface at the time and the horizontally bedded stratigraphic layers by capturing bones and teeth, which fossilised under the favourable chemistry of the spring water[28]. Middle Pleistocene faunal remains were found almost exclusively within spring vents, and for that reason they cannot be associated with specific sedimentary units[3,8].

Microstratigraphic analysis of the site showed that peat is linked to dry periods, whereas sand layers are associated with wet periods that produced bodies of water such as the Soutpan palaeolake and spring-fed ponds of variable depth and surface extent[24]. These interpretations

are supported by the analysis of pollen from peat layers and some of the Middle Pleistocene sand layers, which pointed out more subtle changes within sediment layers, and revealed an alternation of Cyperaceae and aquatic plants indicative of wet conditions (mainly at the top of sand layers and the bottom of peat layers) and with a small fynbos component represented by Ericaceae and Restionaceae, with Amaranthaceae and Asteraceae signalling dry periods[26,29–32]. Pollen results provided the first time-resolved information about past vegetation at Florisbad, although sand layers produced relatively low pollen counts. To fill this gap, bulk sediment samples were collected from the Dreyer Section to extract plant biomarkers, measure their stable isotopes, and assess the magnetic properties of sediments in search of sedimentary gaps. In addition, we collected samples for luminescence dating from the Middle Pleistocene sequence to improve the accuracy of previous dating programmes.

About 6 km southeast of Florisbad, within the Soetdoring Nature Reserve, the Lombard's Drift spring was excavated to assess its potential for archaeological occupations and palaeoenvironmental proxies. Lombard's Drift is mentioned by Grobler & Loock[21] together with the nearby Florisbad and Vlakkraal springs, but no fieldwork had been carried out at the site before we determined its position in July 2022 (28°48'20.36"S; 26°6'37.04"E). The spring is located on the right bank of the Modder River, and it appears as a low-lying spring mound merged with a natural levee surrounded by shallow water and covered by reeds and sedges. In July 2023, we laid out a 2 x 1 m trench on the lower portion of the levee and excavated it until we reached the water table at 1.5 m depth. From top to bottom, we identified four sedimentary units, including i) a thick layer of dark-grey clayey sand, ii) a thin layer of pale-green sandy clay, and iii) a layer of dark-grey clayey sand that transitioned to iv) black peat. No artefacts were found, and only one bone fragment appeared at the bottom of the trench. The latter exhibited a degree of fossilisation similar to what was observed in Middle Pleistocene faunal remains at Florisbad, based on infrared spectroscopy[28]. However, the sedimentary context was rich in organics, which prompted the analysis of plant biomarkers. Samples were also collected for luminescence dating, magnetic susceptibility measurements, and pollen analysis.

SI 2 – Pollen and charcoal analysis at Lombard's Drift

Five samples were collected for pollen and charcoal analysis at Lombard's Drift, parallel to the sediment samples for biomarkers. They were not rich enough to allow reliable pollen counts, but prominent charcoal counts were recorded in the top two layers that show the highest concentrations (Table S11). The low pollen content in the samples could probably be attributed to corrosion due to oxidising conditions under regular, strong seasonal moisture input and desiccation cycles in some levels of the excavated deposits that would not exclude microbial action[29–33]. Higher charcoal content in the upper layers of the excavation could be due to fire as an ecological factor affecting the surrounding grassland areas during the Holocene. Furthermore, charcoal > 100 µm indicates fires that happened close to the site; however, charcoal < 100 µm is indicative of fires on the broader environment.

SI 3 Biomarkers – extended results and discussion

The 21 extracted lipid biomarker samples, covering all layers of the Upper and Lower Dreyer Section at Florisbad, showed good preservation for *n*-alkanes in several layers, while others showed evidence of substantial degradation of *n*-alkane chains. Indicators for degradation are high even-chain homologues signals, low extraction amounts, and low carbon preference

index (CPI) values (Table S2). Particularly poor preservation, indicated by a value of CPI < 2, is present at the bottom of the sequence (Units P, O), parts of Peat II (Units G, H), and most of the Holocene units (Units A to D). Humic acid identified in marshy layers could be one explanation for bad organic preservation in the sandy layers[32]. However, the picture is not as consistent for the *n*-alkanes. What is clear is that age is not a main influence on *n*-alkane preservation, otherwise we would expect significantly better preservation in the Holocene units, which is not the case.

Ten samples from Florisbad, all with good CPI values, were prepared for stable isotope measurement, and eight could be measured successfully. Sample UD01 spilled in the vial before measurement, and sample UD03 had too low concentrations to be measured. The amount of *n*-alkanes at Florisbad shows a wide range across samples from 42.3 ng/g (Sample UD13) to 6005.5 ng/g sediment (Sample LD15) when adding up the C_{26}-C_{33} homologues. However, the concentrations should not be taken as exact values and are an overestimation. When measuring the stable isotopes in the mass spectrometer, we noticed that concentrations were by a factor of two lower across samples when compared to the results calculated from the gas chromatograph measurement. However, as this is a consistent overestimation that does not vary, the general trend in distribution of individual homologue abundances and indices calculations should not be affected. The amount of *n*-alkanes at Lombard's Drift is on average higher than at Florisbad and ranges from 1182.40 ng/g (Sample LBD3) to 2474.75 ng/g (Sample LBD4) when adding up the C_{24}-C_{35} homologues. The Lombard's Drift samples were prepared and measured a year later, with a new internal standard, and there is no discrepancy in concentrations between gas chromatograph and mass spectrometer measurements.

Florisbad's location within an active spring, subject to fluctuating water tables both past and present, introduces a complex array of potential biomarker sources. While aeolian sediment units likely present a local to regional scale factor that influence vegetation cover, units with evidence for wetness need to consider water transport, peat formation and aquatic vegetation. Previously published Florisbad pollen studies suggested that during high lake levels the local lake edge pioneers representative of perennial drying are present in the pollen record, masking a regional vegetation signal[29–32]. Global studies of lake sediments and surrounding plants suggest that leaf wax input tends to be dominated by direct leaf waxes of local woody plants[34–39]. Furthermore, good correlation between soil wax biomarkers and modern biome distributions for South Africa[40,41] suggests a local signal should be present. Modern contamination must be considered in an open-air site context where some sediment units show extensive bioturbation[24]. Modern contamination has been demonstrated for pollen in parts of Test Pit 2 for example, but not so extensively for the Main Excavation[32], where our samples were collected. The consistent difference between Holocene and Pleistocene units, the good match with the pollen record and the varying preservation, all suggest that modern contamination is not influencing the *n*-alkane signal in any significant manner.

The Florisbad sequence is made of four well-identified organic sediment layers that were called "peat layers" (Peat I-IV; Table S1). Peats are usually organic-rich, made up of decomposed plant material that can produce C_{19}-C_{33} *n*-alkanes *in-situ*, reflecting a very local signal[42]. However, most studies on peats are from the northern hemisphere in temperate environments and few studies exist for tropical and subtropical Africa [e.g. [43,44]]. Aquatic plants that grow under water, and often contribute to peat accumulation, can use inorganic

carbon pools that are affected by a range of factors other than atmospheric CO_2, complicating the interpretation of stable isotope values.

A primary control on ^{13}C is submergent vs emergent plant physiology, with submerged plants enriched in $\delta^{13}C$, especially in peats[42]. At Florisbad, the *n*-alkane homologue distributions do not show a clear abundance of C_{23}-C_{25} for submergent plants, however, there are higher abundances of these homologues in some samples (Figure S4). At both Florisbad and Lombard's Drift, the relative abundance of homologues is most likely a mixture of plant types (terrestrial vs aquatic and C_3 vs C_4 plants) and degradation. The P_{aq} index is often used to determine the amounts of submerged, emergent and terrestrial plants in sediment samples[45,46]. Generally, P_{aq} values of < 0.1 indicate terrestrial plant input, while values ranging between 0.1 – 0.7 signal emergent macrophytes or a mixture of inputs, and values of > 0.7 point to submerged macrophyte inputs[45]. At Florisbad, P_{aq} ranges between 0.21 and 0.69 (Table S2). This indicates a varying mix of terrestrial and emergent macrophytes in all layers, with higher indices closer to 0.7 (sample FLB12, Peat III/Unit E) potentially having additionally submerged macrophytes in the mix. In comparison, the P_{aq} at LBD with a narrow span from 0.45 to 0.52 across all samples (Table S2) indicates a potential combination of terrestrial and emergent macrophytes, and is in the same range as the modern samples from Florisbad. However, the δD values for LBD, on average are higher than at FLB, indicating mainly terrestrial plant sources and do not overlap with arid zone values for algae and submergent plants (arid zone mean -160 ‰)[47].

What stands out is the unusually high nC_{33} concentrations in most of the Pleistocene samples (Units E to N, Figure S4). Sample FLB24 was taken to test the hypothesis that the unusually high C_{33} abundances are representing marsh conditions with reeds such as today. The results suggest that this is not the case. More than 50 % of the Cyperaceae in the summer rainfall region today, (e.g. *Phgragmites australis*, common at Lombard's Drift) are C_3 plants[48]. A recent study by Tweedy et al.[49] on high elevation C_3 grasses in Kenya showed that C_3 grasses from the PACMAD clade (containing the subfamilies Panicoideae, Arundinoideae, Chloridoideae, Micrairoideae, Aristidoideae and Danthonioideae) produce very long chain *n*-alkanes, showing C_{33} or C_{35} maxima in abundance. While we have very high C_{33} maxima, we have very low or no C_{35} in our samples. Other C_3 grasses show distribution patterns in the abundance of homologues that are indistinguishable from C_4 grass or C_3 woody plants. Furthermore, it is unclear how comparable the modern humid, closed mountain habitat from Kenya is with the past Free State environment. However, there are studies showing that there is much yet to learn about C_3 grasses and their presence in our samples cannot be excluded[49]. Considering the distribution of C_3 grasses in the interior of South Africa it is known that they are associated with cool growing seasons, and this explains their prominence in the highlands of the eastern escarpment[50]. Another influence could be CAM plants with high nC_{33} homologues, such as Aizoaceae[51]. High pollen concentrations of Aizoaceae have been found between Peat I and Peat II[32]. The high concentration of C_{33} is the main influence on the $\delta^{13}C$ weighted average signal, meaning that our $\delta^{13}C$ values could reflect changes in C_3/C_4 vegetation, with $\delta^{13}C$ values < -30 ‰ indicating C_3 plant origin.

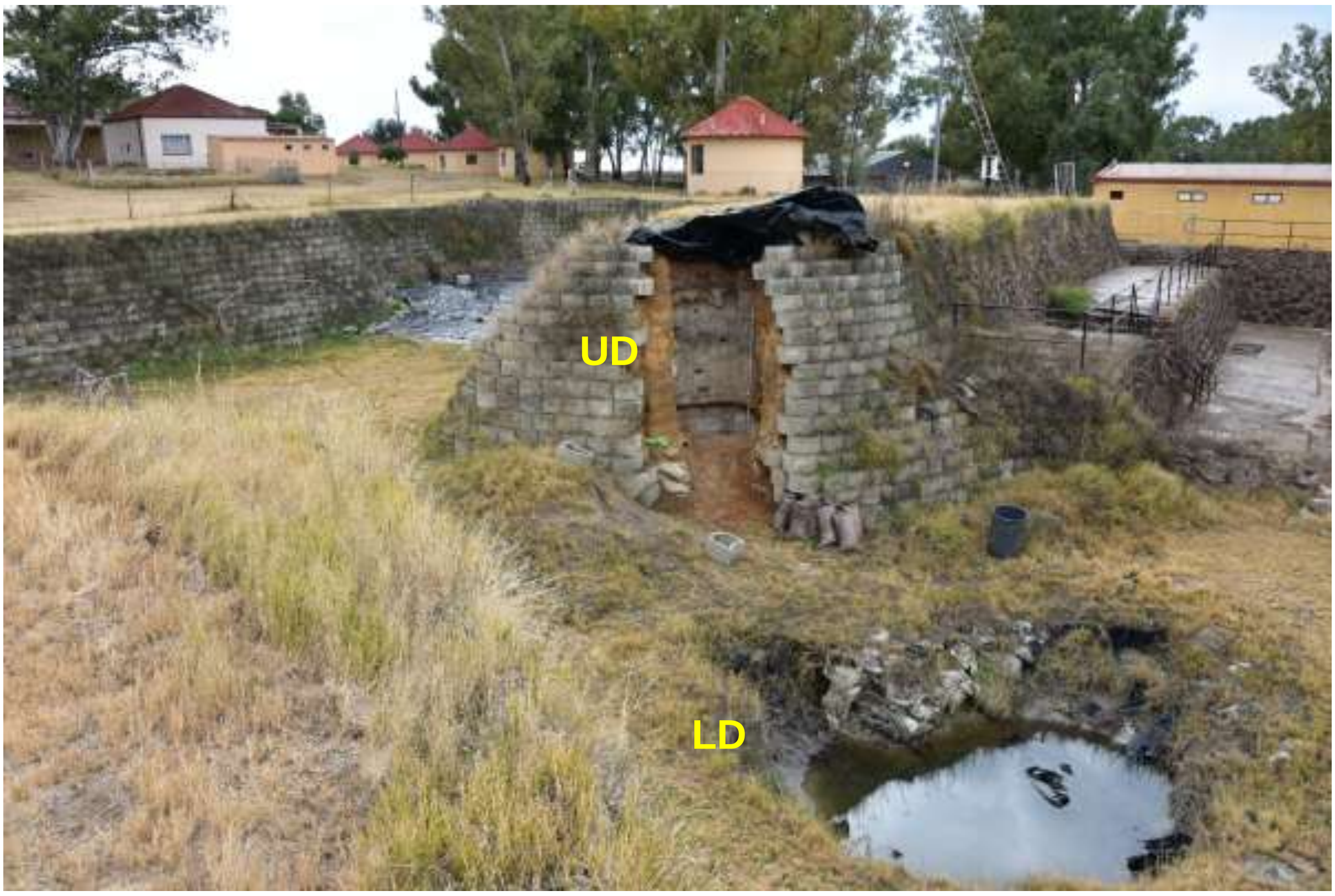

Figure S1. The Dreyer Section before sample collection. The upper portion (UD) spans the Late Pleistocene and Holocene, whereas the lower portion (LD) includes the Middle Pleistocene. Note the level of the water table, which was pumped out in order to collect sediment samples.

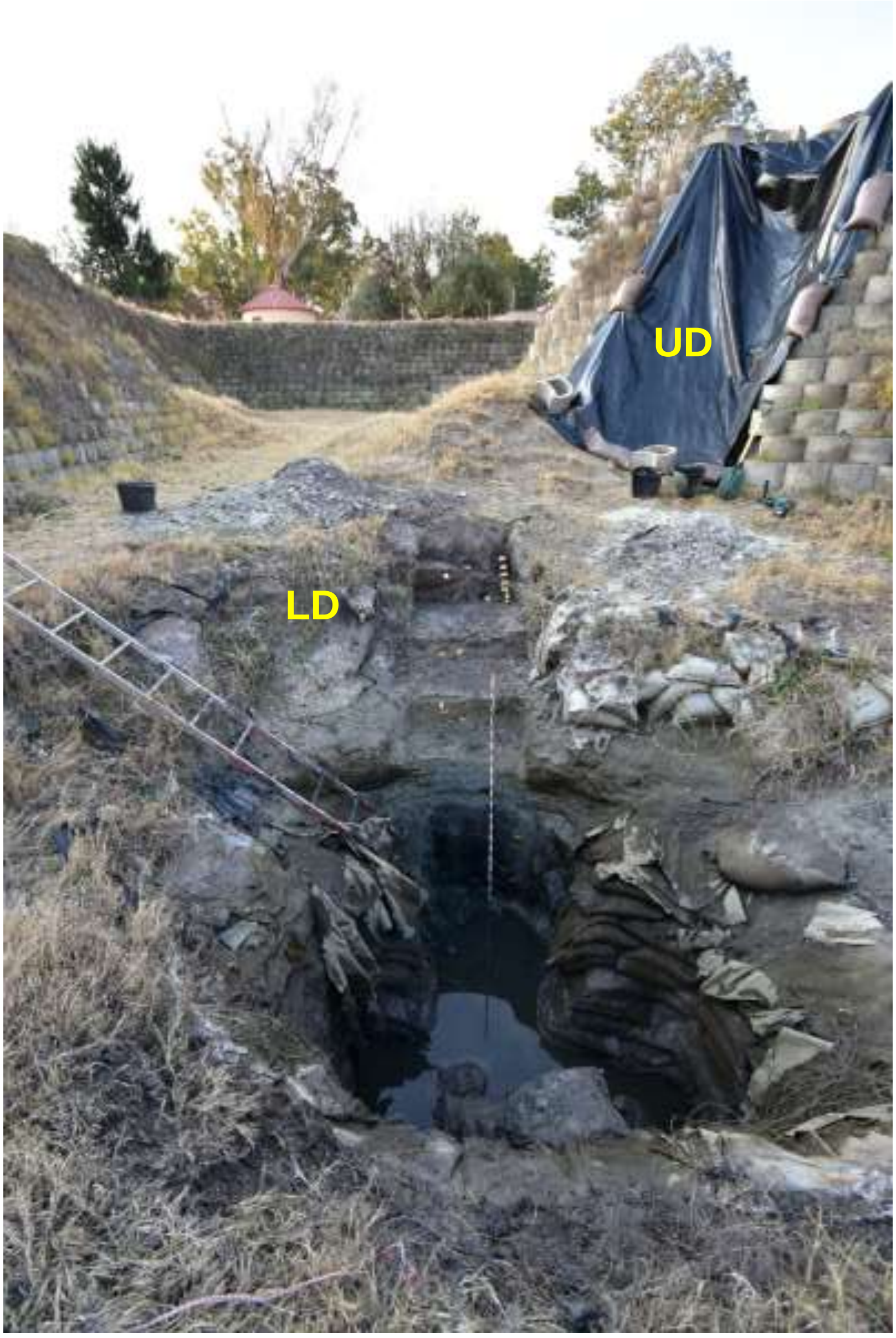

Figure S2. The lower Dreyer Section during sample collection.

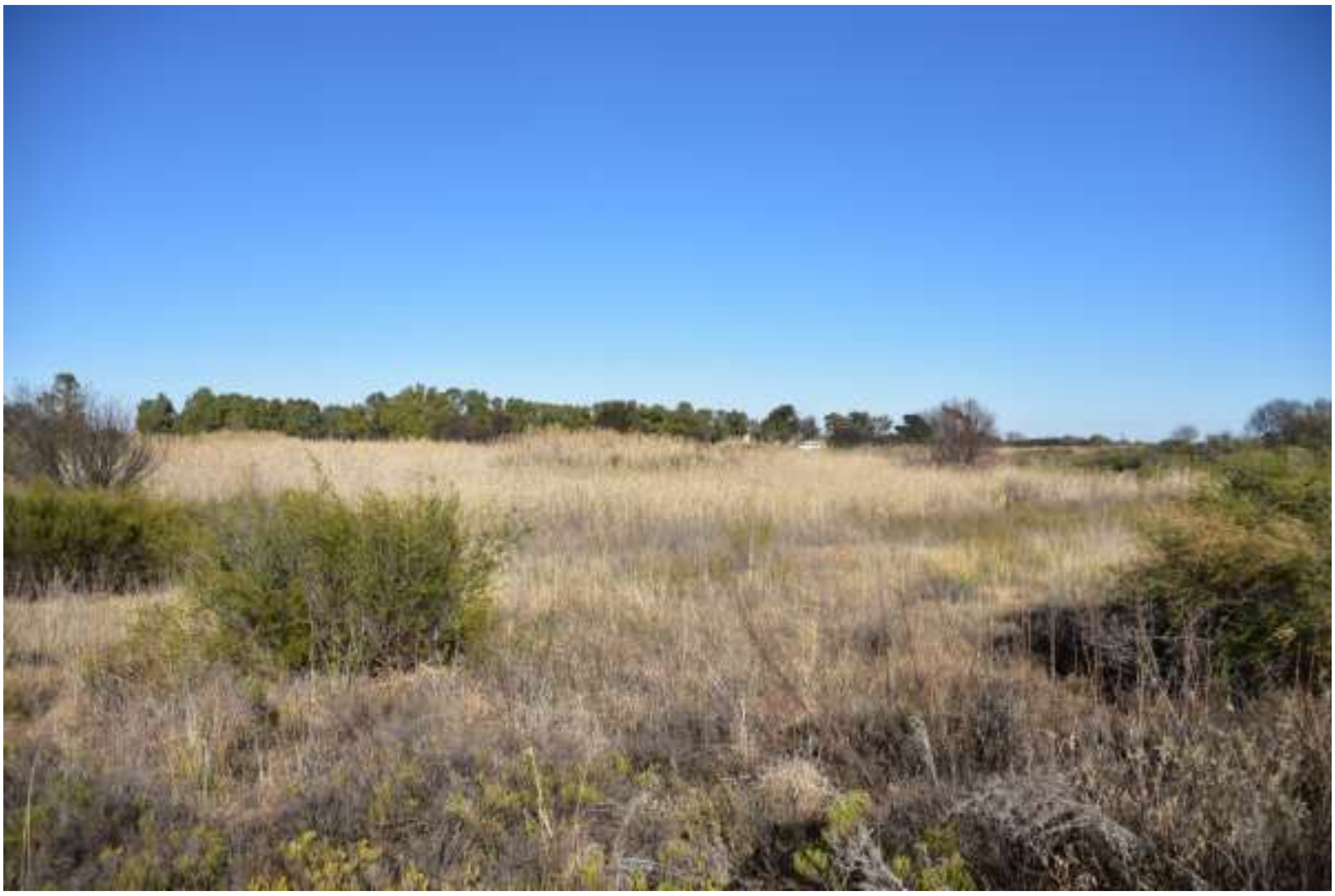

Figure S3. The Lombard's Drift spring, pictured from the north. Note the small mound in the centre of the photo, covered in reeds. The line of trees in the background marks the Modder riverbed.

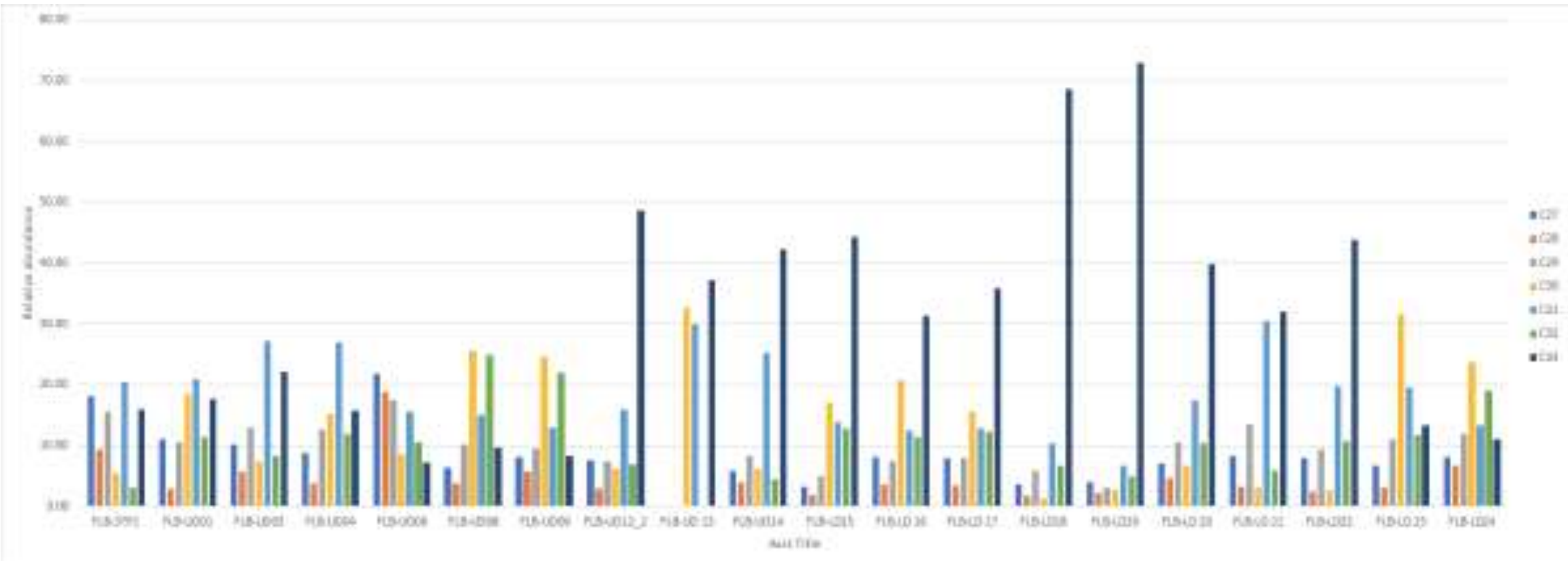

Figure S4. Relative abundance of C_{27}-C_{33} *n*-alkane homologues at Florisbad. Samples are ordered from youngest to oldest. Because of the low concentrations of *n*-alkanes in the samples, C_{25} and C_{35} could not be detected in many samples.

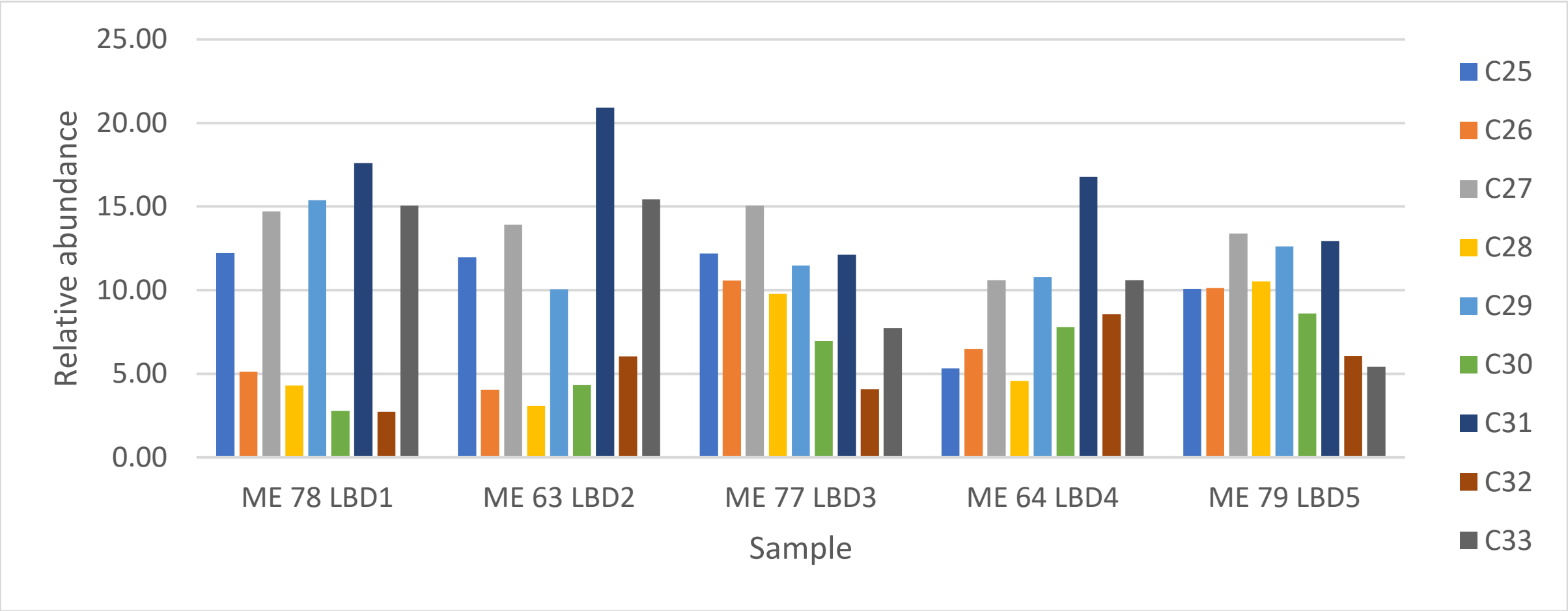

Figure S5. Relative abundance of C_{25}-C_{35} *n*-alkane homologues at Lombard's Drift. Samples are ordered from youngest to oldest.

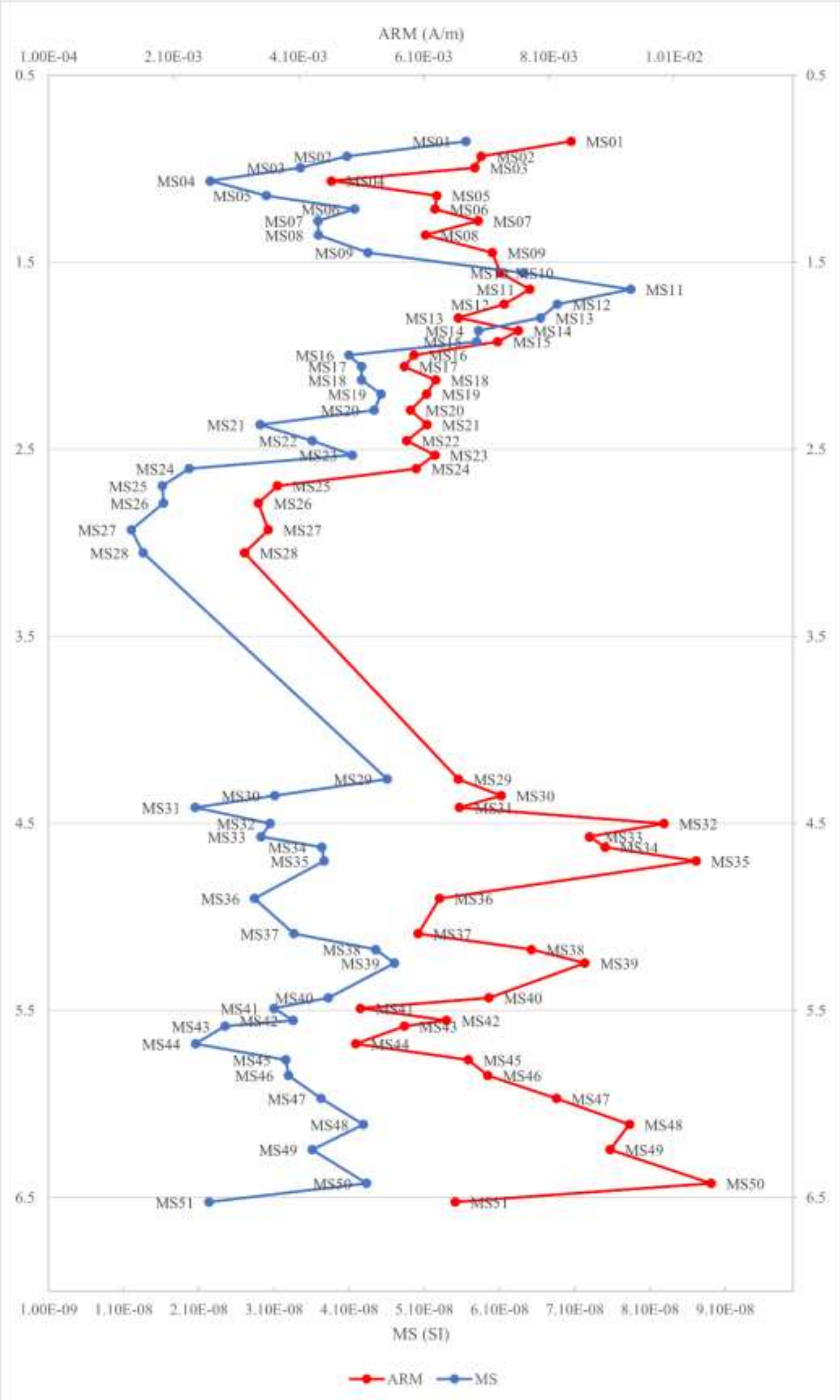

Figure S6. Magnetic susceptibility (MS) and anhysteretic remanent magnetisation (ARM) values for the Dreyer Section.

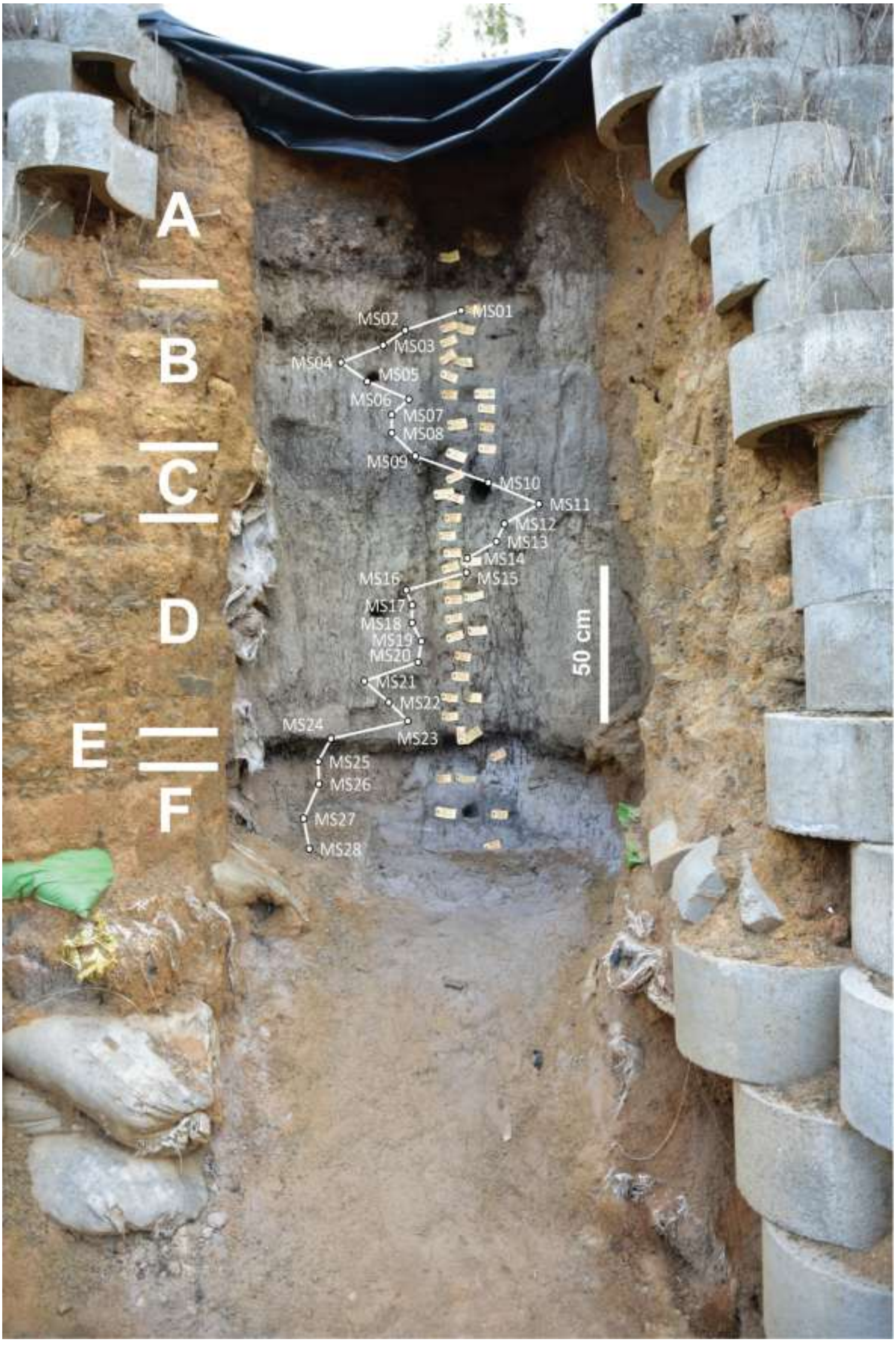

Figure S7. Magnetic susceptibility shifts for the upper portion of the Dreyer Section.

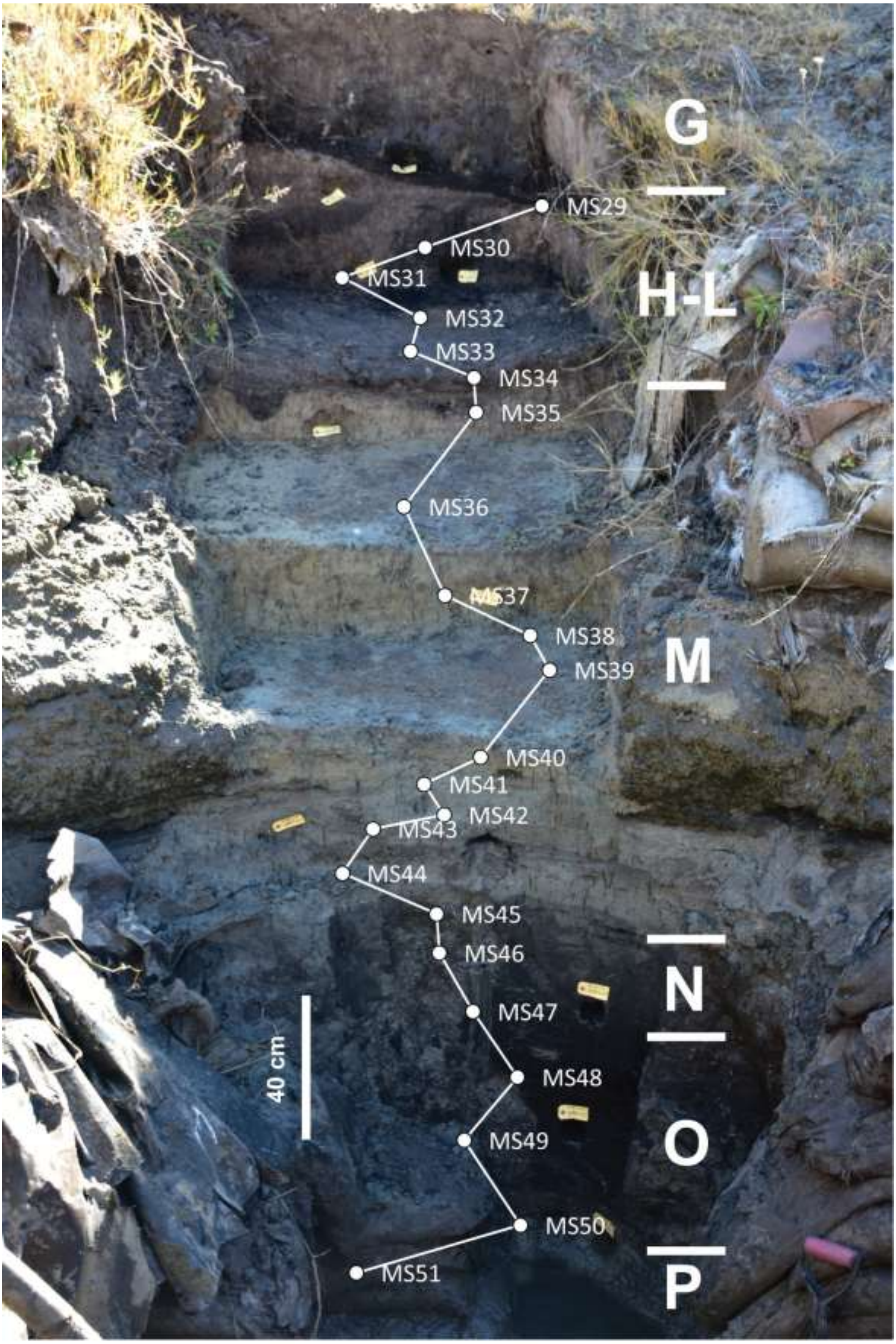

Figure S8. Magnetic susceptibility shifts for the lower Dreyer Section.

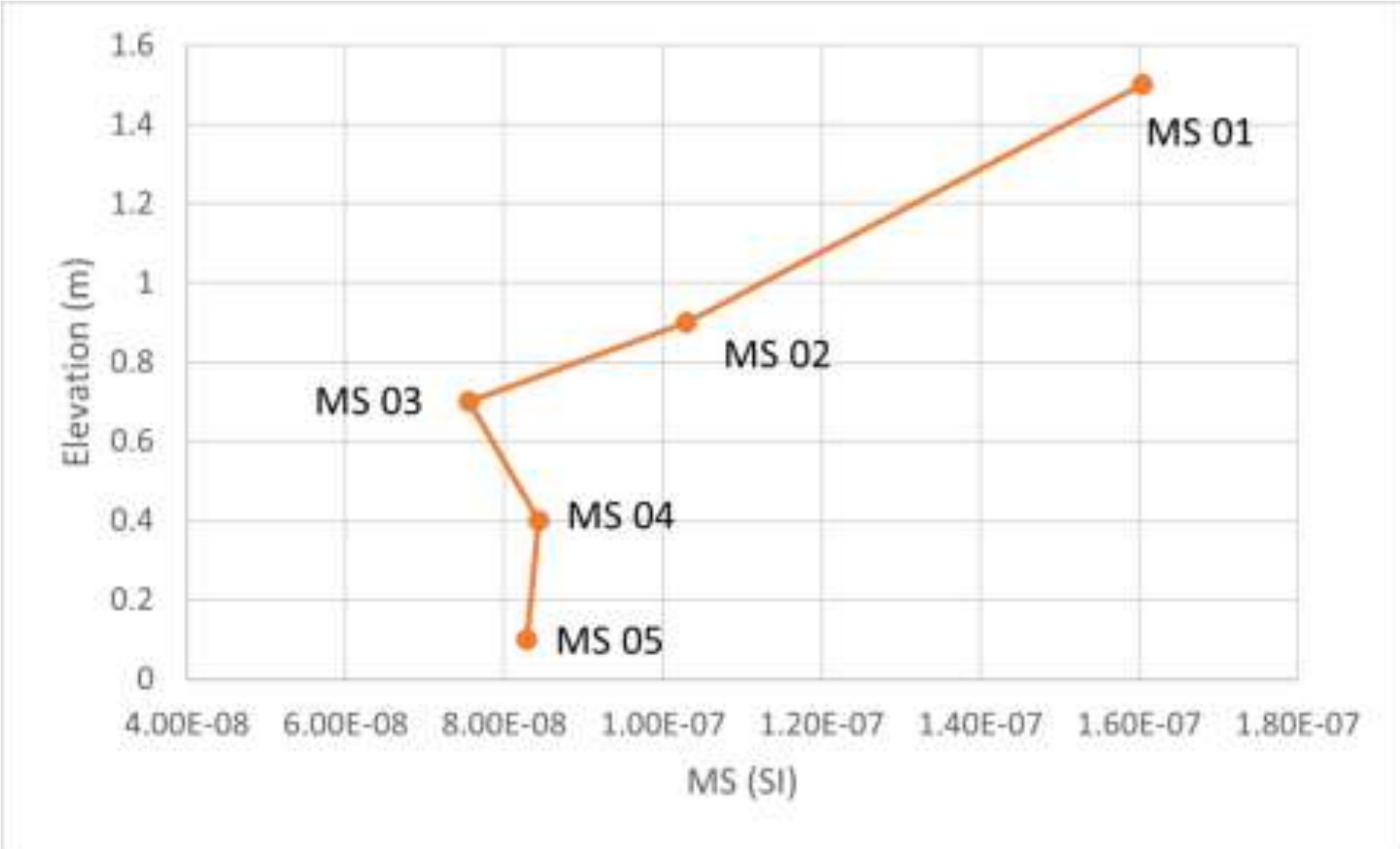

Figure S9: Magnetic susceptibility values for Lombard's Drift.

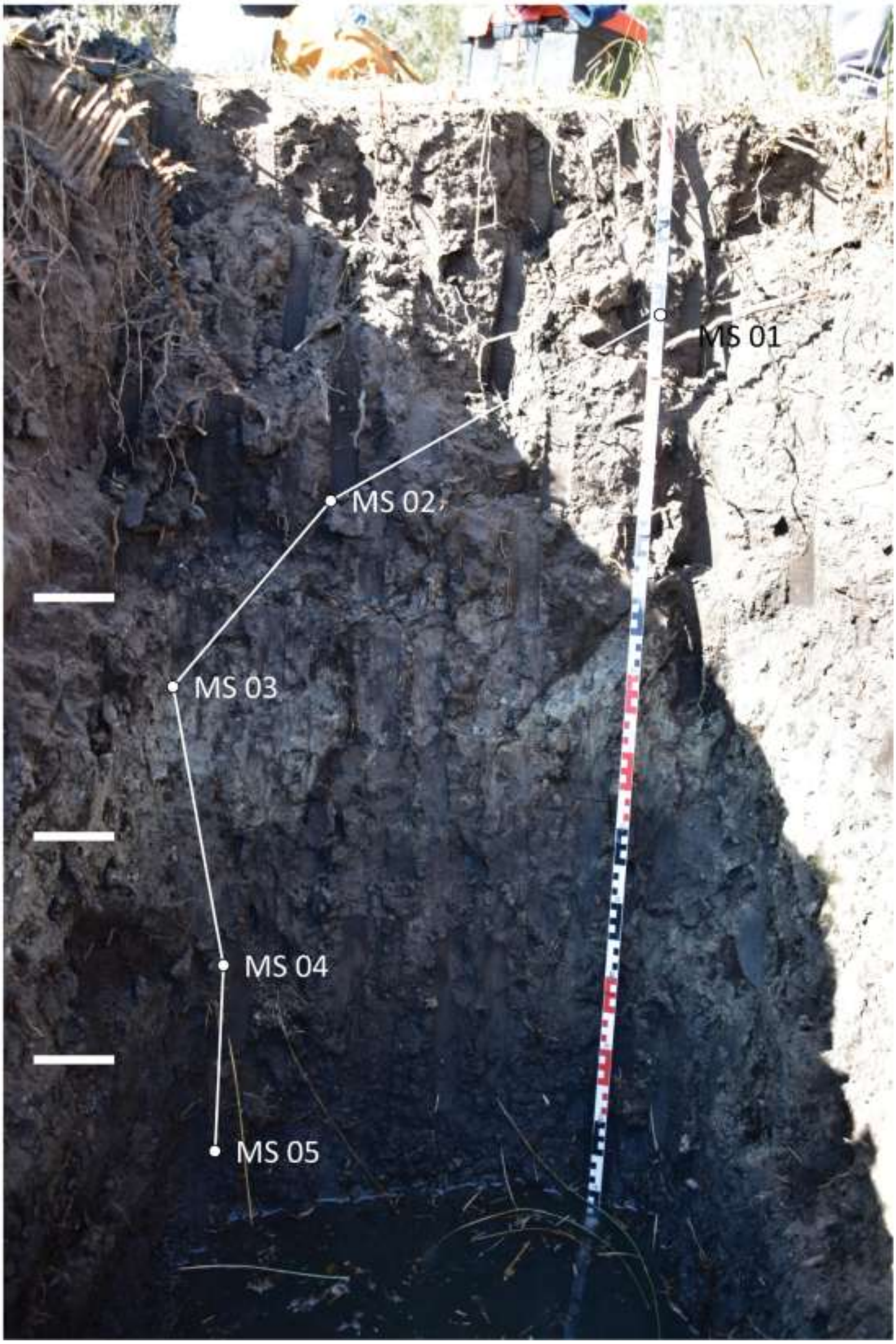

Figure S10. Magnetic susceptibility shifts for Lombard's Drift (colour changes in scale bar every 20 cm).

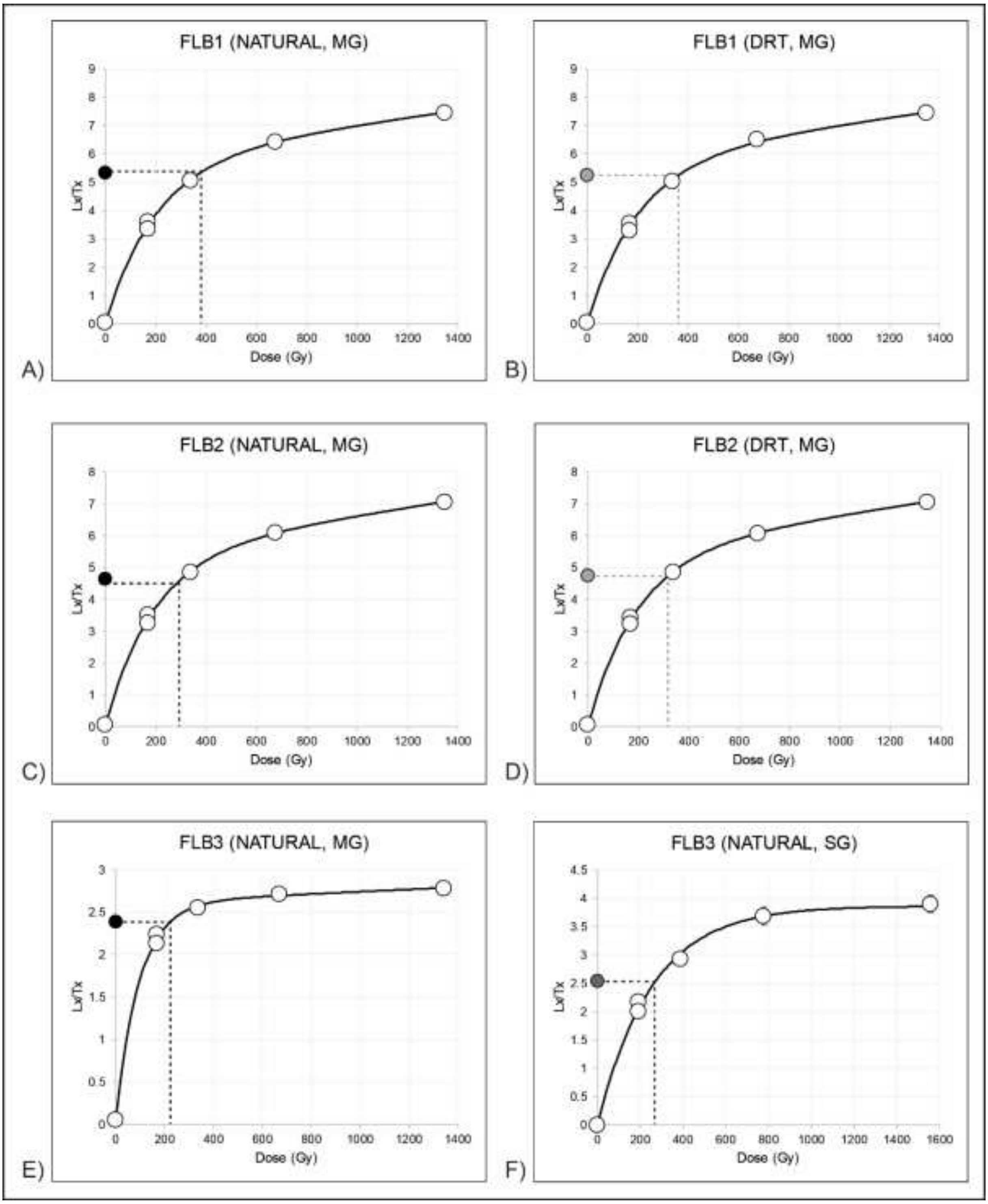

Figure S11. Dose response curves (DRC) obtained for FLB1 and FLB2 (4-11 µm), and FLB3 (100-140 µm) on a natural multigrain aliquot (A = 380 ± 22 Gy; C = 303 ± 15 Gy; E = 226 ± 20 Gy), on a natural single grain of FLB3 (F = 275 ± 21 Gy), and on an irradiated aliquot of FLB1 and FLB2 (B: given dose = 364 Gy; recovered dose = 364 ± 19 Gy; D: given dose = 309 Gy; recovered dose = 322 ± 16 Gy). D_0 for natural samples are 206 ± 20 Gy (A), 193 ± 18 (C), 96 ± 10 Gy (E), and 254 ± 18 Gy (F).

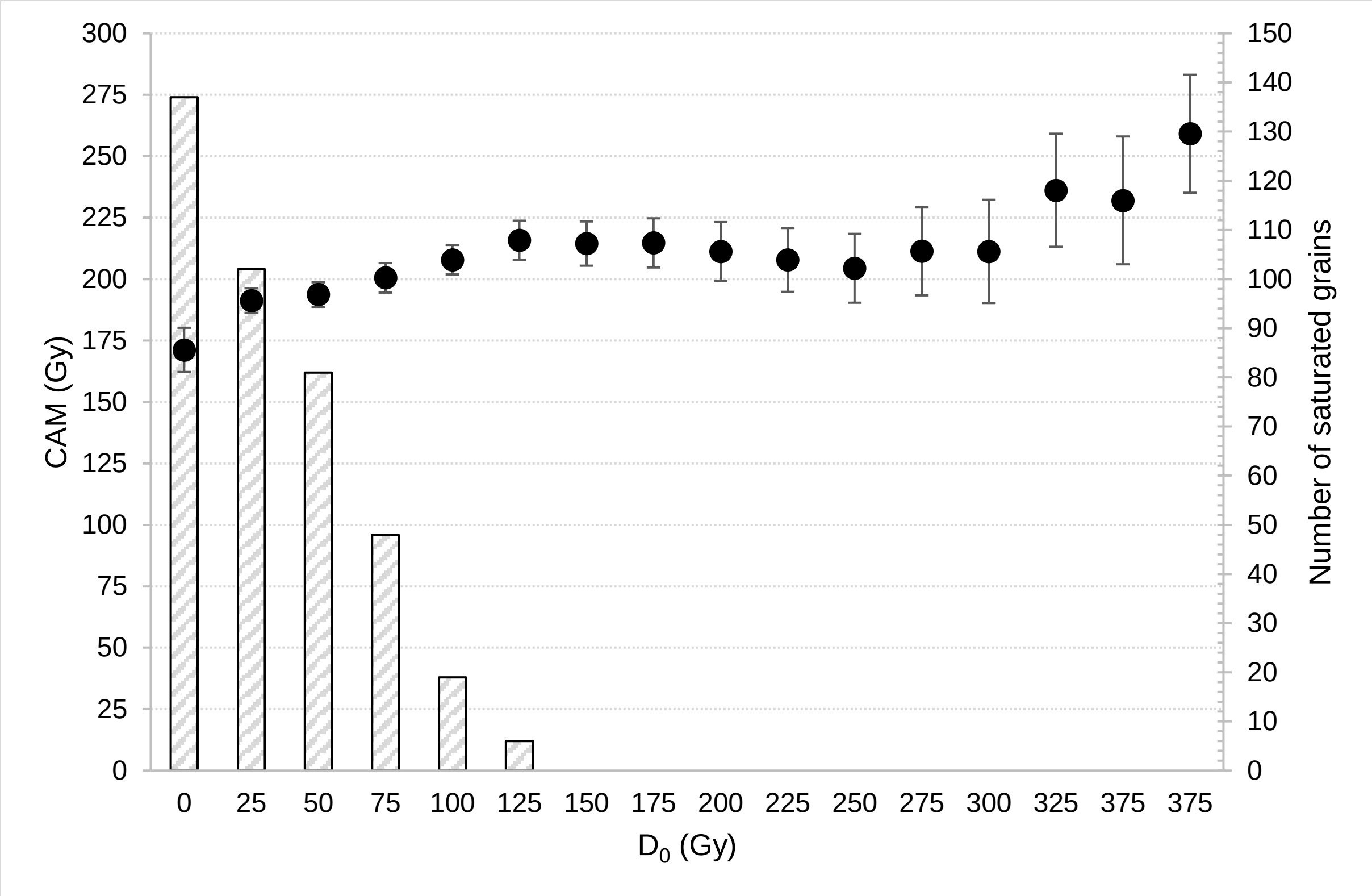

Figure S12. Number of saturated grains (i.e., grains that passed the selection criteria but for which no intersection of the L_N/T_N signal on the growth curve was reached), and CAM values calculated increasing the D_0 threshold from 0 to 375 Gy. Starting from a value of 150 Gy, no saturated grains are observed.

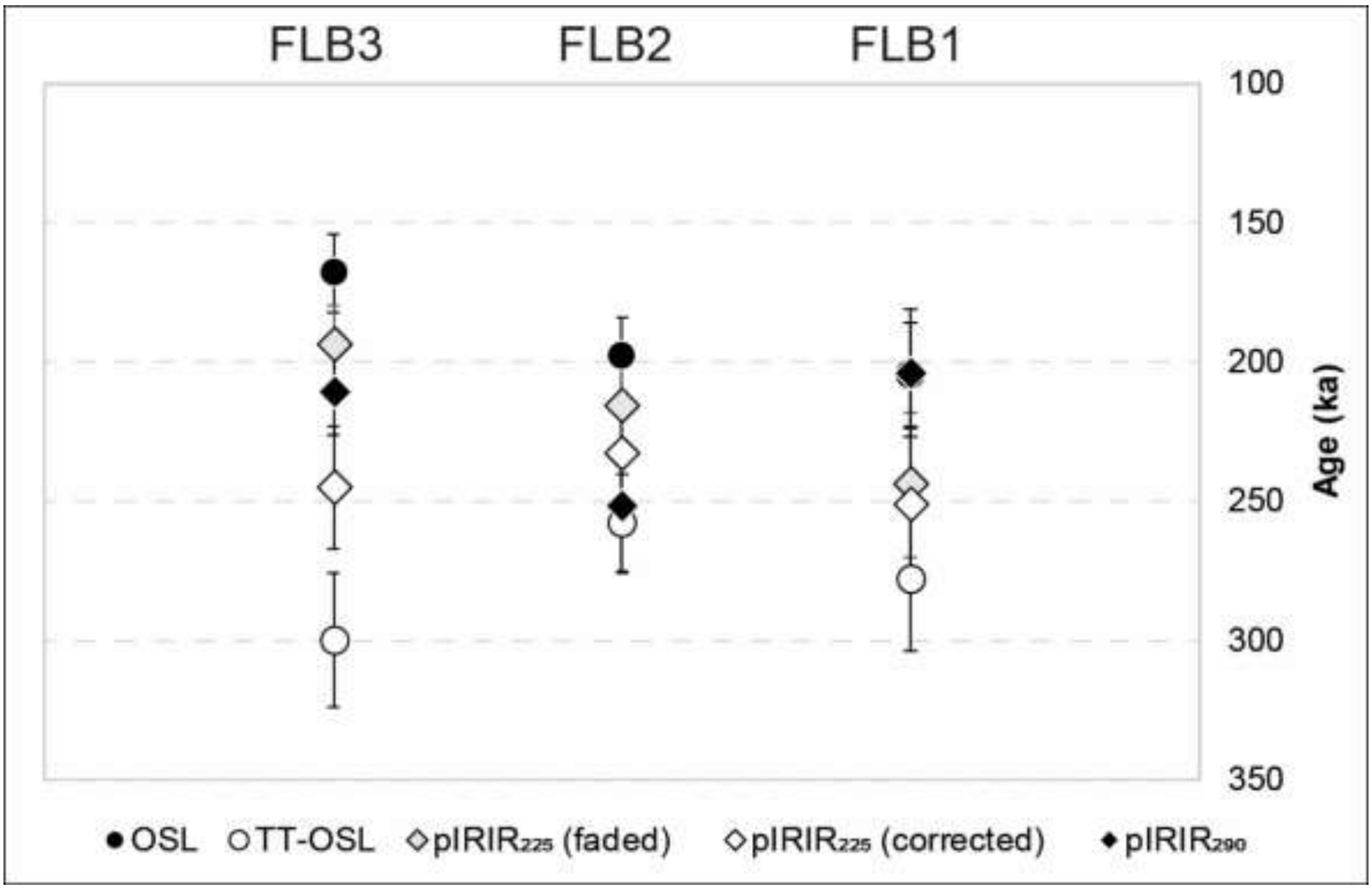

Figure S13. Synthesis of the ages obtained on quartz (circles) and feldspars (diamonds).

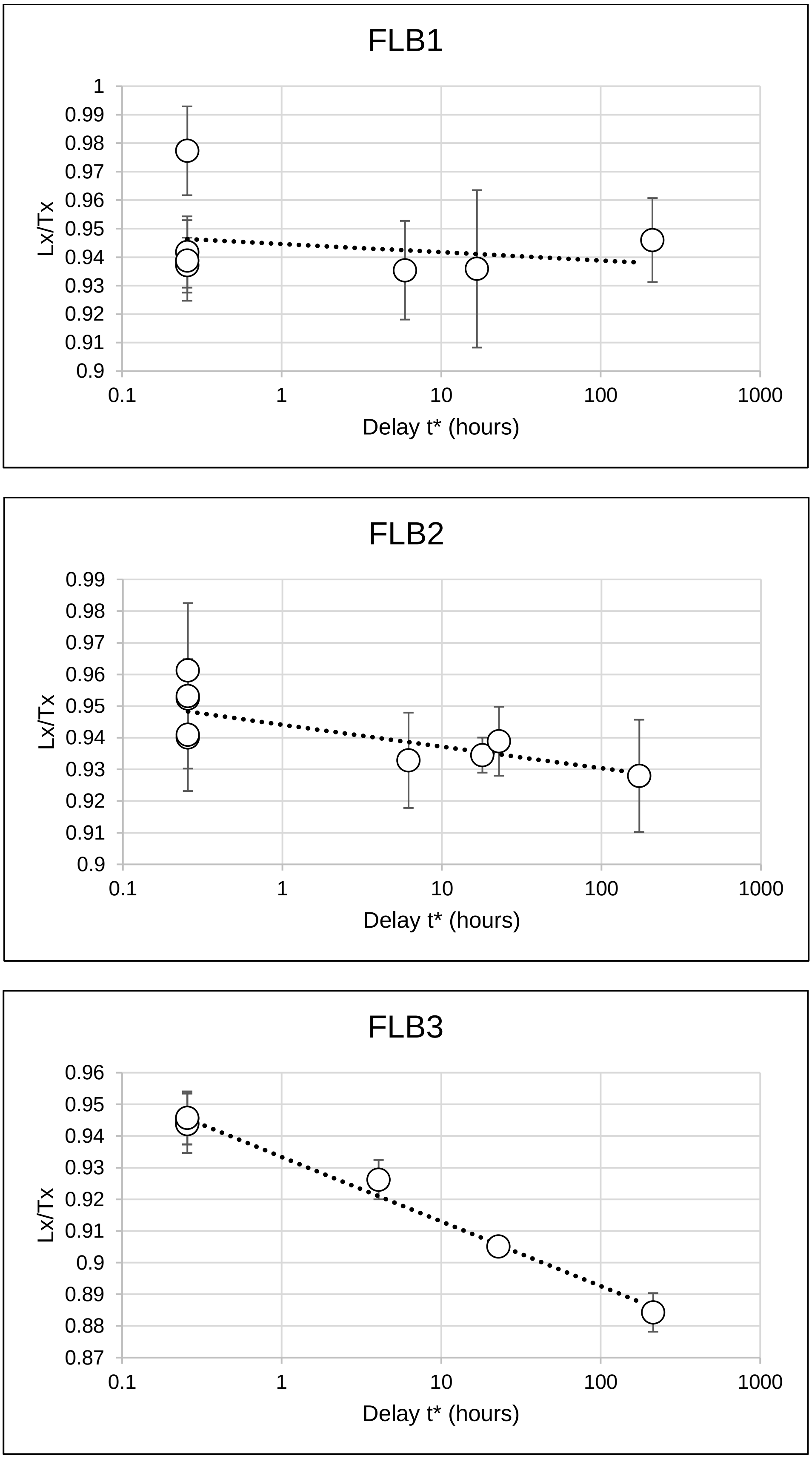

(a) (b) (c)

Figure S14. Fading test results obtained for the polymineral fraction of FLB1 (a) and FLB2 (b) and the K feldspars of FLB3 (c). Each point represents the mean L_x/T_x value with the standard deviation obtained on three to five MG aliquots measured for each delay time.

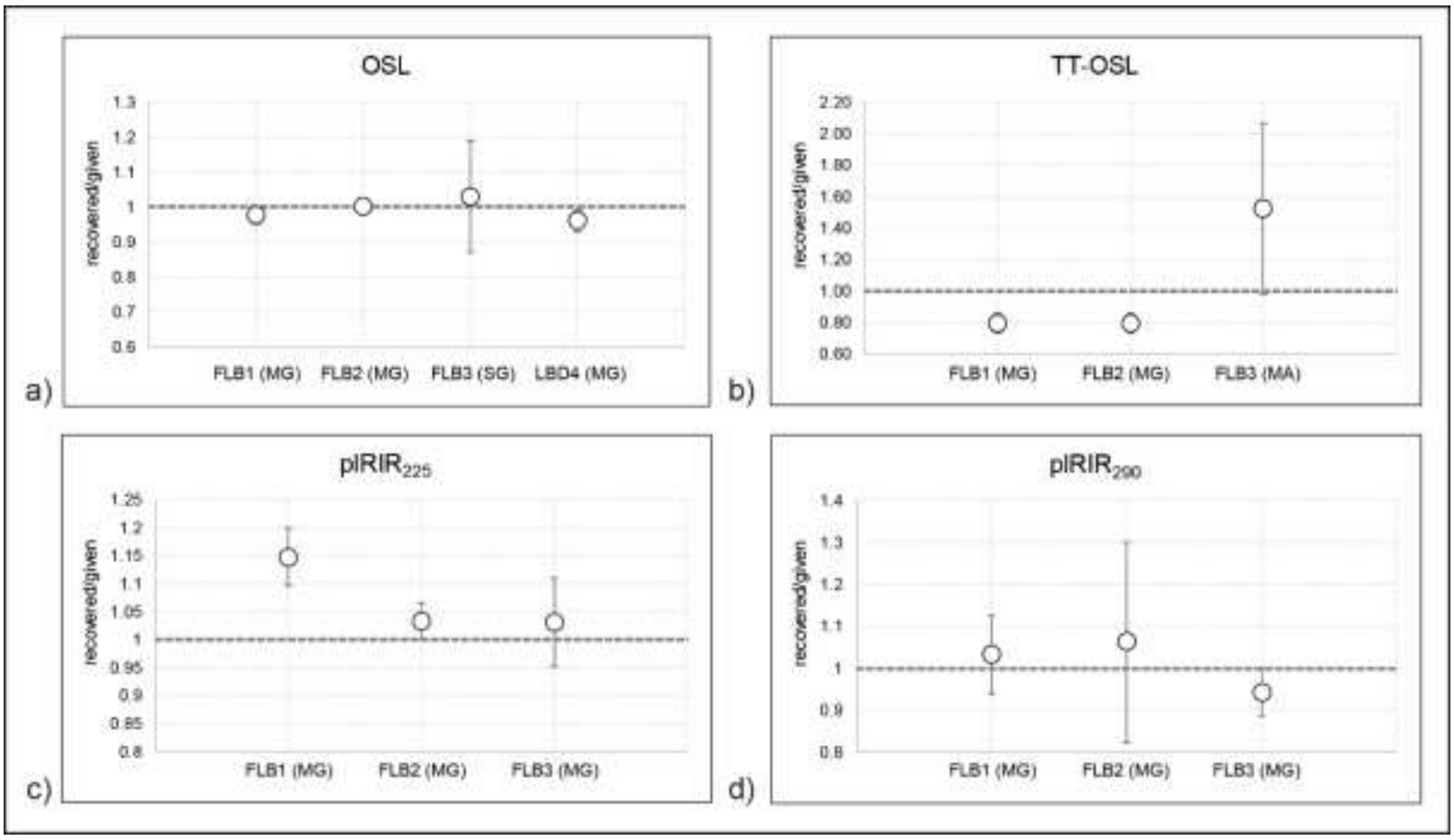

Figure S15. Dose recovery tests results obtained for each protocol on the Florisbad (FLB) and Lombard's Drift (LBD) samples. MG = multigrain; SG = single grain; MA = micro-aliquots.

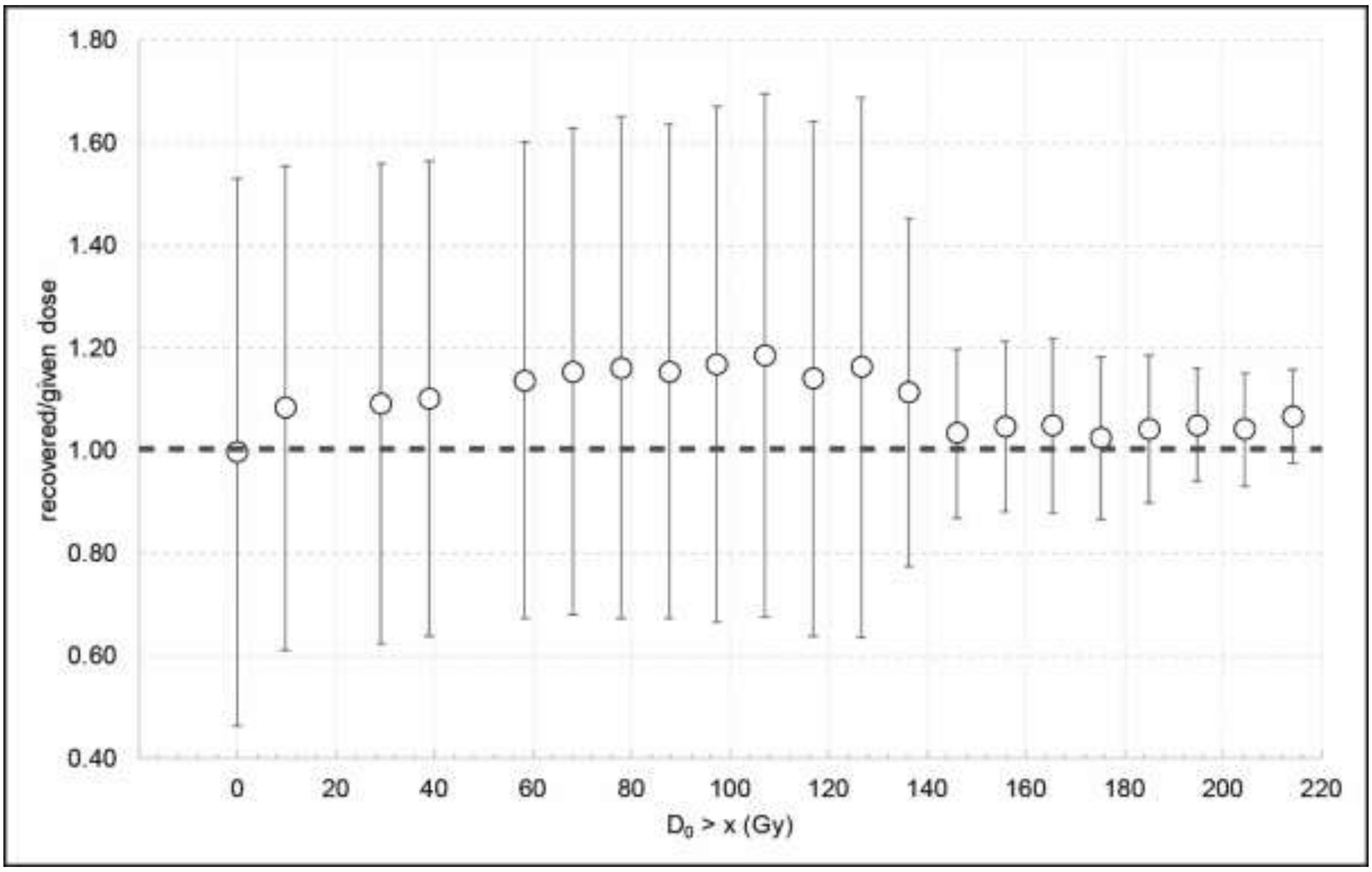

Figure S16. SG OSL DRT values of FLB3 sorted as a function of the D_0 threshold. The recovered/given dose ratio was calculated increasing the minimum D_0 threshold, from 0 to 214 Gy. The grey dashed line indicates the recovered/given dose value of 1. Starting from a value of 140 Gy, no saturated grains are observed.

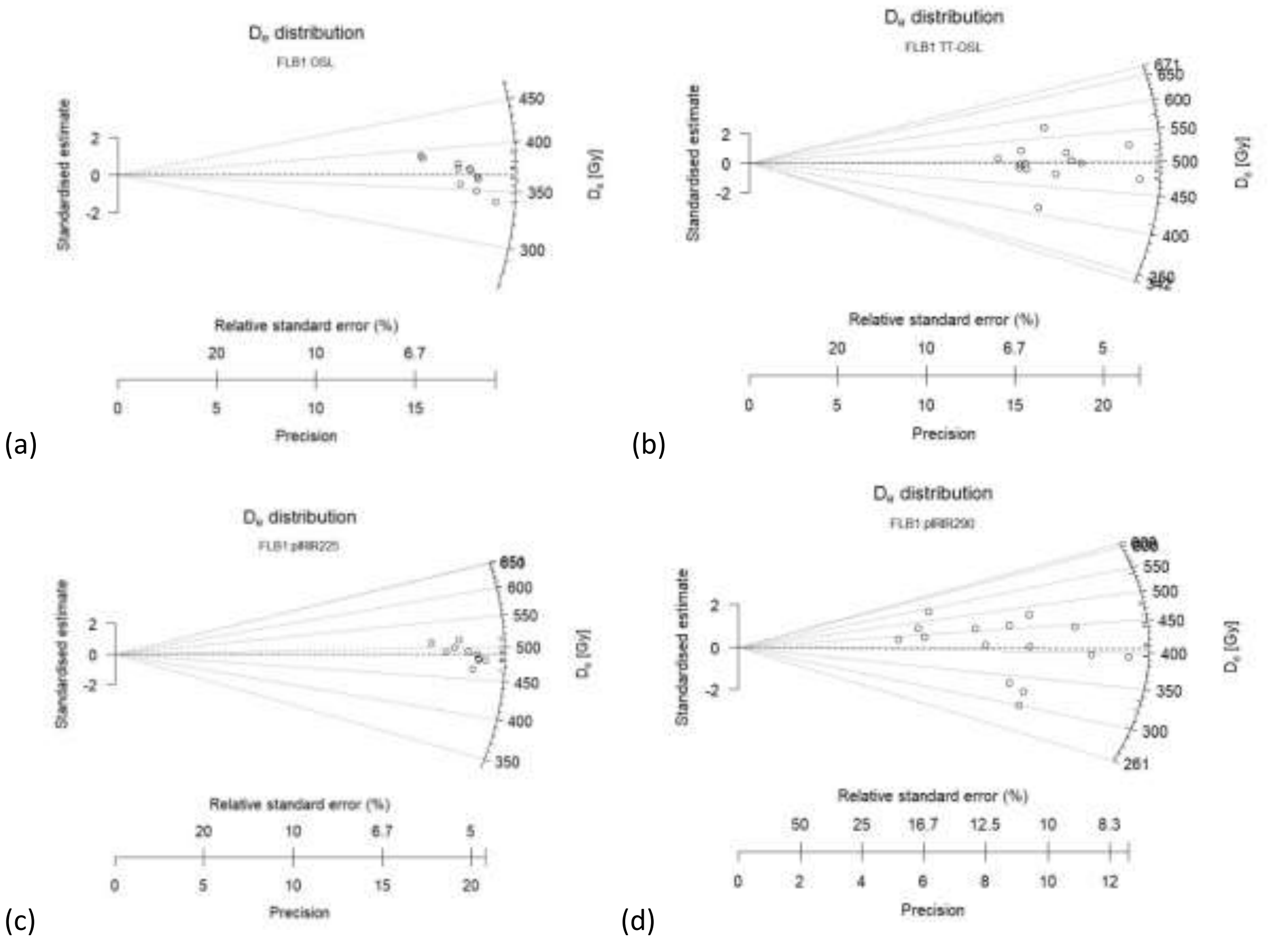

Figure S17. Radial plots showing the multigrain D_e distribution for FLB1 (a) OSL; (b) TT-OSL; (c) $pIRIR_{225}$ and (d) $pIRIR_{290}$. Plots were created using the plot_RadialPlot() function in the R package Luminescence[53].

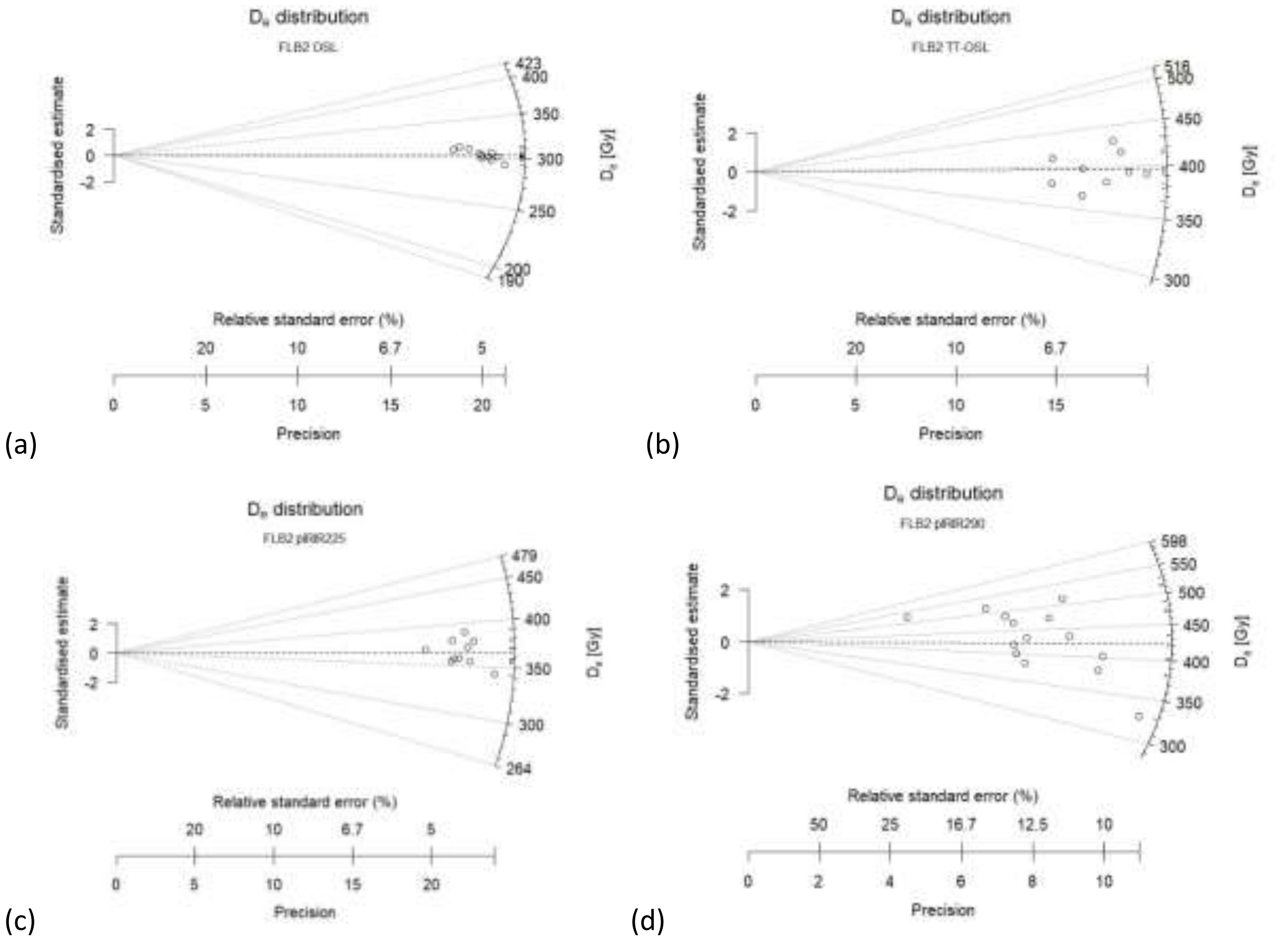

Figure S18. Radial plots showing the multigrain D_e distribution for FLB2 (a) OSL; (b) TT-OSL; (c) $pIRIR_{225}$ and (d) $pIRIR_{290}$. Plots were created using the plot_RadialPlot() function in the R package Luminescence[53].

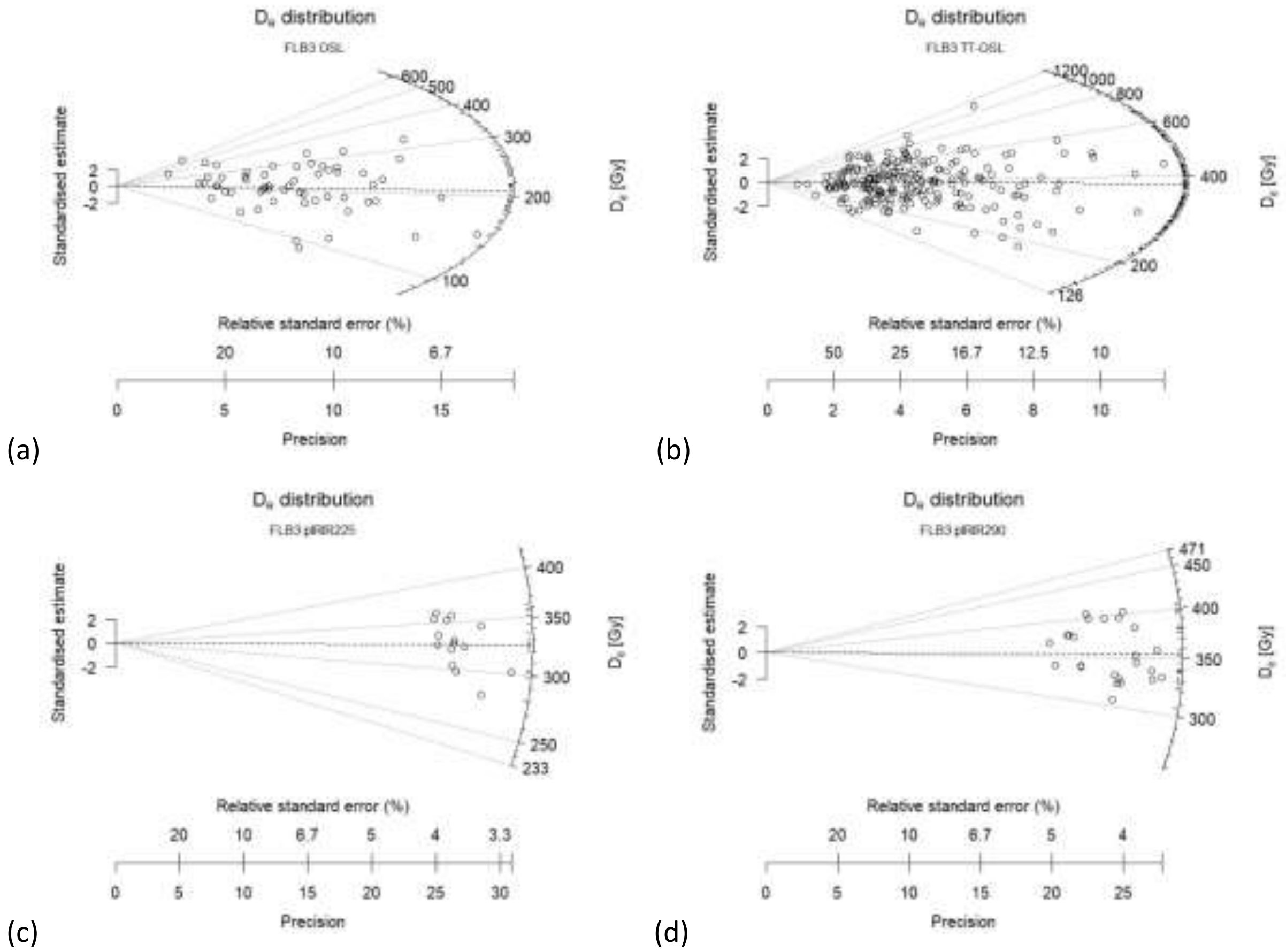

Figure S19. Radial plots showing the D_e distribution for FLB3 (a) single grain OSL; (b) micro-aliquot TT-OSL; (c) $pIRIR_{225}$ multigrain and (d) $pIRIR_{290}$ multigrain. Plots were created using the plot_RadialPlot() function in the R package Luminescence[53].

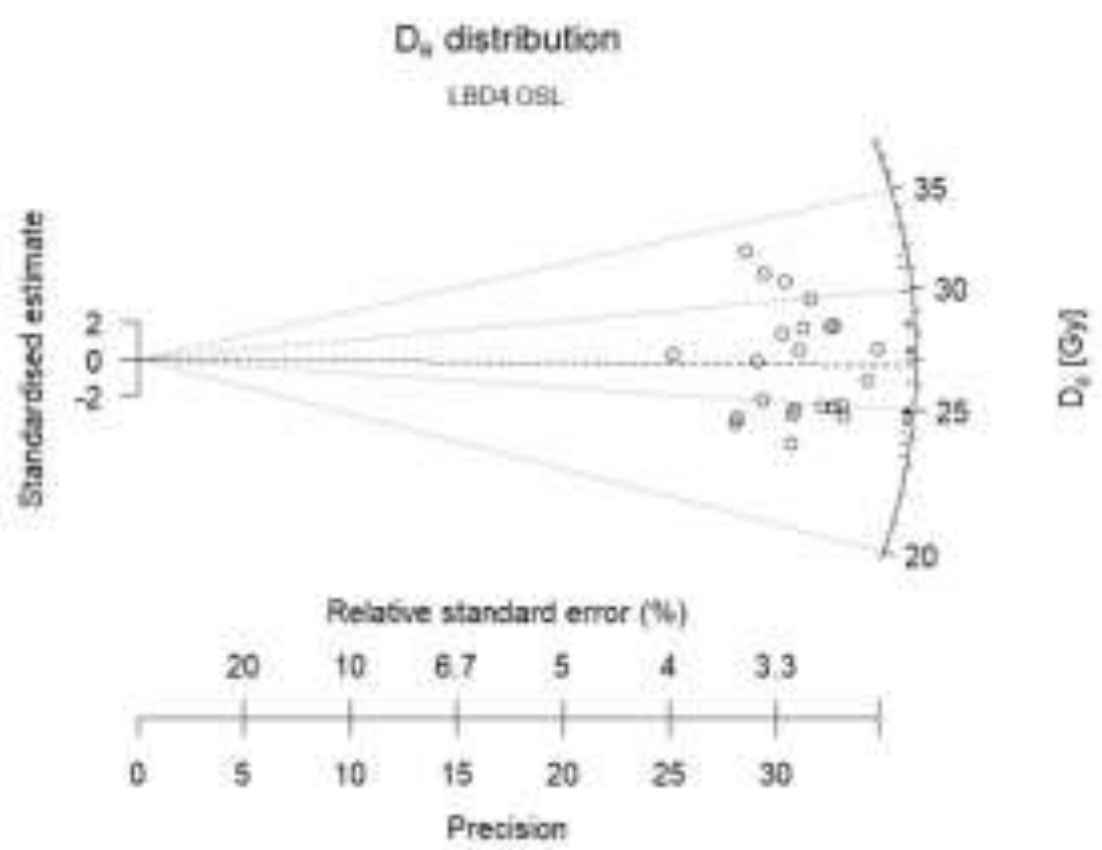

Figure S20. Radial plots showing the multigrain OSL D_e distribution for LBD4. Plot was created using the plot_RadialPlot() function in the R package Luminescence[53].

Table S1. Sedimentary Units identified at Florisbad. [a]: Test Pit 2 and the Main Excavation do not include Units 1-3 of Toffolo et al.[24]. Age ranges according to Grün et al.[15], Scott & Nyakale[26], and Pinder[27].

Test Pit 2[a] and 3	Corresponding Main Excavation[a] units
1: LSA, 3.1-0.8 ka	-
2: LSA, 9.1-3.8 ka	-
3	-
4: 12.1-9.4 ka	B (Peat IV): LSA, 3.3-1.7 ka; C: 7.4-4.2 ka; D: 13.3-8.1 ka
5 (Peat III)	E (Peat III): MSA
6: MSA, 170-111 ka	F: MSA, 138-121 ka
7 (Peat II): 141-124 ka	G to L (Peat II): MSA, 131 ka
8: 213-149 ka	M: MSA, 157-149 ka; N: MSA, 191 ka
9 (Peat I): 176-161 ka	O (Peat I): early MSA, 281-136 ka
10: 254 ka	P: early MSA, 280-185 ka
11: Ecca shale, Permian	Q: Karoo dolerite, Jurassic

Table S2. Distribution of biomarkers from Florisbad and Lombard's Drift. From left to right: Sample name and location (Florisbad) or depth below surface (Lombard's Drift), Stratigraphic Unit after Kuman et al.[10] for Florisbad; Dating after Scott & Nyakale[26] (^{14}C), Grün et al.[15] (OSL, ESR) and new OSL dates; Sum (ng/µl), *Norm*31 and *Norm*33 index; Average chain length (ACL) of the C_{27}-C_{33} *n*-alkanes; Carbon Preference Index (CPI) of the C_{27}-C_{33} *n*-alkanes; P_{aq} after Ben Arous et al.[46] formula B.

**Sample**	**Section**	**Unit & cultural material**	**Ages**	$\boldsymbol{Norm_{31}}$	$\boldsymbol{Norm_{33}}$	$\mathbf{ACL_{27\text{-}33}}$	$\mathbf{CPI_{27\text{-}33}}$	$\mathbf{P_{aq}}$
				**Florisbad**				
FLB24	Modern marsh	n/a	2024	0.53	0.49	30.00	5.65	0.59
3TP1	3rd test pit	Late Holocene LSA, remains of domestic stock & pottery	Last 2000 y	0.57	0.51	29.97	2.35	0.63
UD1	Upper Dreyer section	A		0.67	0.63	30.50	1.51	0.66
UD2	Upper Dreyer section	B LSA	*1710-3170 BP*			Not extracted		
UD3	Upper Dreyer section	B LSA	*1710-3170 BP*	0.68	0.63	30.69	2.62	0.49
UD4	Upper Dreyer section	B LSA	*1710-3170 BP*	0.68	0.55	30.55	1.78	0.36
UD5	Upper Dreyer section	B LSA	*1710-3170 BP*			Not extracted		
UD6	Upper Dreyer section	C	*4220-6290 BP*	0.47	0.29	29.26	1.63	0.21

UD7	Upper Dreyer section	C	*4220-6290 BP*	Not extracted				
UD8	Upper Dreyer section	D	*8170±90 BP*	0.59	0.49	30.35	0.71	0.32
UD9	Upper Dreyer section	D	*8170±90 BP*	0.58	0.47	30.10	0.64	0.49
UD10	Upper Dreyer section	D	*8170±90 BP*	Not extracted				
UD11	Upper Dreyer section	D	*8170±90 BP*	Not extracted				
UD12	Upper Dreyer section	E MSA		0.68	0.87	31.66	3.88	0.73
UD13	Upper Dreyer section	F MSA		n/a	n/a	32.11	2.06	0.43
UD14	Upper Dreyer section	F		0.75	0.84	31.55	4.46	0.45
LD15	Lower Dreyer section	G MSA	121 ± 6 ka (ESR)	0.74	0.90	31.98	1.98	0.28
LD16	Lower Dreyer section	H MSA		0.63	0.81	31.26	1.46	0.46

LD17	Lower Dreyer section	H MSA		0.62	0.82	31.37	1.82	0.48
LD18	Lower Dreyer section	I MSA		0.64	0.92	32.26	7.63	0.67
LD19	Lower Dreyer section	M MSA	157 ± 21 ka (OSL)	0.69	0.96	32.43	6.52	0.62
LD20	Lower Dreyer section	M MSA	157 ± 21 ka (OSL)	0.63	0.79	31.41	2.97	0.47
LD21	Lower Dreyer section	M MSA	168 ± 14 ka (OSL)	0.69	0.70	31.05	5.38	0.63
LD22	Lower Dreyer section	N Early MSA	198 ± 14 ka (OSL)	0.68	0.83	31.46	4.19	0.62
LD23	Lower Dreyer section	O Early MSA	205 ± 19 ka (OSL), 281 ± 73 ka (OSL)	0.64	0.55	30.56	1.03	0.16
LD24	Lower Dreyer section	P Early MSA	279 ± 47 ka (OSL)	0.53	0.48	30.23	0.79	0.33
			**Lombard's Drift**					
LBD1	0.4 m	n/a	Holocene	0.68	0.61	30.29	3.57	0.49
LBD2	0.6 m	n/a	Holocene	0.51	0.40	29.51	1.75	0.49
LBD3	0.8 m	n/a	Holocene	0.61	0.50	30.20	1.73	0.52
LBD4	1.1 m	n/a	11.2 ± 0.8 ka (OSL)	0.51	0.30	29.53	1.42	0.45

LBD5	1.3 m	n/a	unknown	0.75	0.51	30.44	2.67	0.45

Table S3. Statistics on the selected single grains for FLB3. [1]Total number of individual quartz grains measured using the SAR protocol; [2]Number of grains that provided a luminescence signal passing the following criteria: recycling ratio ≠ 1 ± 10 %; recuperation <5 %; error on the test dose <10 %; T_n signal > 3 σ above background; [3]Among the grains that passed criteria, those for which the D_0 was ≥ to 150 Gy (see Fig. S12). [4]Number of grains used to calculate the equivalent dose using the CAM.

FLB3	
Number of grains measured[1]	1500
Number of grains that passed criteria[2]	309
Number of rejected grains[3]	250
Accepted grains[4]	59

Table S4. U, Th, and K contents of sediment samples analysed with a laboratory gamma-ray spectrometer equipped with a high-resolution, broad energy Ge (BEGe) detector, and measured water content.

**Sample**	**U (ppm)**	**Th (ppm)**	**K (%)**	**Water content (%)**
FLB1	2.43 ± 0.10	7.62 ± 0.07	1.42 ± 0.02	59
FLB2	1.17 ± 0.04	4.24 ± 0.03	0.95 ± 0.01	24
FLB3	1.14 ± 0.07	4.31 ± 0.05	0.83 ± 0.01	18
LBD4	2.17 ± 0.02	9.20 ± 0.09	1.60 ± 0.02	22

Table S5. OSL MG SAR protocol applied to quartz.

**Step**	**Treatment**
1	Given dose
2	Preheat (260 °C for 10 s)
3 (L_x)	Blue stimulation for 100 s at 125 °C
4	Given test dose
5	Cutheat (200 °C for 0 s)
6 (T_x)	Blue stimulation for 100 s at 125 °C
7	Return to 1

Table S6. TT-OSL MG SAR protocol applied to quartz.

Step	Treatment
1	Given dose
2	Preheat (260 °C for 10 s)
3	Blue stimulation for 300 s at 125 °C
4	Preheat (260 °C for 10 s)
5 (L_x)	Blue stimulation for 100 s at 125 °C
6	Given test dose
7	Preheat (220 °C for 10 s)
8 (T_x)	Blue stimulation for 100 s at 125 °C
9	Optical draining (blue stimulation for 100 s at 300 °C)
10	Return to 1

Table S7. $pIRIR_{225}$ MG SAR protocol applied to feldspars.

Step	Treatment
1	Given dose
2	Preheat (250 °C for 60 s)
3	Infrared stimulation for 100 s at 50 °C
4 (L_x)	Infrared stimulation for 100 s at 225 °C
5	Given test dose
6	Preheat (250 °C for 60 s)
7	Infrared stimulation for 100 s at 50 °C
8 (T_x)	Infrared stimulation for 100 s at 225 °C
9	Optical draining (infrared stimulation for 40 s at 290 °C)
10	Return to 1

Table S8. $pIRIR_{290}$ MG SAR protocol applied to feldspars.

Step	Treatment
1	Given dose
2	Preheat (320 °C for 60 s)
3	Infrared stimulation for 200 s at 50 °C
4 (Lx)	Infrared stimulation for 200 s at 290 °C
5	Given test dose
6	Preheat (320 °C for 60 s)
7	Infrared stimulation for 200 s at 50 °C
8 (Tx)	Infrared stimulation for 200 s at 290 °C
9	Optical draining (infrared stimulation for 100 s at 325 °C)
10	Return to 1

Table S9. OSL SG SAR protocol applied to quartz.

Step	Treatment
1	Given dose
2	Preheat (260 °C for 10 s)
3 (Lx)	SG green laser stimulation for 1 s
4	Given test dose
5	Cutheat (200 °C for 0 s)
6 (Tx)	SG green laser stimulation for 1 s
7	Optical draining (blue stimulation for 120 s at 280 °C)
8	Return to 1

Table S10. TT-OSL MA SAR protocol applied to quartz.

Step	Treatment
1	Given dose
2	Preheat (260 °C for 10 s)
3	Green stimulation for 100 s at 125°C
4	Preheat (260 °C for 10 s)
5 (Lx)	SG green laser stimulation for 2 s at 125°C
6	Given test dose
7	Preheat (220 °C for 10 s)
8 (Tx)	SG green laser stimulation for 2 s at 125°C
9	Optical draining (green stimulation for 100 s at 300 °C)
10	Return to 1

Table S11. Table of the pollen counts and concentrations found in the Lombard's Drift profile. Samples are numbered from top to bottom and were collected in the same spot as biomarker and magnetic susceptibility samples.

	Pollen 1		Pollen 2		Pollen 3		Pollen 4		Pollen 5	
	Count	Concentration	Count	Concentration	Count	Concentration	Count	Concentration	Count	Concentration
Poaceae	4	38	2	3	6	5	1	23	5	12
Cyperaceae	2	19	-	-	1	1	-	-	4	10
Asteraceae	2	19	4	7	-	-	-	-	-	-
Tribulus	-	-	1	2	-	-	2	46	-	-
Cheno/Am	-	-	-	-	2	2	-	-	-	-
Searsia	-	-	-	-	2	2	-	-	-	-
Acacia	-	-	-	-	1	1	2	46	-	-
Typha	-	-	-	-	-	-	-	-	25	60
Un-identified	-	-	-	-	1	1	-	-	1	1
Pseudoschizea	-	-	-	-	6	5	-	-	-	-
Charcoal > 100 µm	266	2509	86	143	5	4	9	208	8	19
Charcoal < 100µm	424	4000	182	305	21	17	67	1546	84	201
Fungi round	2	19	1	2	-	-	-	-	5	12
Fungi triangular	37	349	-	-	2	2	1	23	-	-

References

1. Kuman, K. & Clarke, R. J. Florisbad—New Investigations at a Middle Stone Age Hominid Site in South Africa. *Geoarchaeology* **1**, 103–125 (1986).
2. Kuman, K. Florisbad, South Africa. in *Handbook of Pleistocene Archaeology of Africa: Hominin behavior, geography, and chronology* (eds Beyin, A., Wright, D. K., Wilkins, J. & Olszewski, D. I.) 1451–1467 (Springer International Publishing, Cham, 2023). doi:10.1007/978-3-031-20290-2.
3. Brink, J. S. *The Archaeozoology of Florisbad, Orange Free State*. (National Museum, Bloemfontein, 1987).
4. Brink, J. S. The taphonomy and palaeoecology of the Florisbad spring fauna. *Collect. Palaeoecol. Afr. Surround. Isl.* 169–179 (1988).
5. Brink, J. S. & Lee-Thorp, J. A. The feeding niche of an extinct springbok, Antidorcas bondi (Antelopini, Bovidae), and its paleoenvironmental meaning. *South Afr. J. Sci.* **88**, 227–229 (1992).
6. Manegold, A. & Brink, J. S. Descriptions and palaeoecological implications of bird remains from the Middle Pleistocene of Florisbad, South Africa. *Paläontol. Z.* **85**, 19–32 (2011).
7. Van Couvering, J. A. & Delson, E. African Land Mammal Ages. *J. Vertebr. Paleontol.* **40**, e1803340 (2020).
8. Dreyer, T. F. The archaeology of the Florisbad deposits. *Argeologiese Navorsinge Van Nas. Mus. Bloemfontein* **1**, 65–77 (1938).
9. Meiring, A. J. D. The Macrolithic Culture of Florisbad. *Navorsinge Van Nas. Mus. Bloemfontein* **1**, 205–237 (1956).
10. Kuman, K., Inbar, M. & Clarke, R. J. Palaeoenvironments and Cultural Sequence of the Florisbad Middle Stone Age Hominid Site, South Africa. *J. Archaeol. Sci.* **26**, 1409–1425 (1999).
11. Henderson, Z. The integrity of the Middle Stone Age horizon at Florisbad, South Africa. *Navorsinge Van Nas. Mus. Bloemfontein* **17**, 25–52 (2001).
12. Brink, J. S. & Henderson, Z. L. A high-resolution last interglacial MSA horizon at Florisbad in the context of other open-air occurrences in the central interior of southern Africa: an interim statement. in *Middle Palaeolithic and Middle Stone Age settlement systems* (ed. Conard, N. J.) 1–20 (Kerns Verlag, Tübingen, 2001).
13. Dreyer, T. F. A human skull from Florisbad, Orange Free State, with a note on the endocranial cast, by C.U. Ariëns Kappers. *K. Akad. Van Wet. Te Amst.* **38**, 3–12 (1935).
14. Clarke, R. J. A new reconstruction of the Florisbad cranium, with notes on the site. in *Ancestors: the hard evidence* (ed. Delson, E.) 301–305 (Alan R. Liss, New York, 1985).
15. Grün, R. *et al.* Direct dating of Florisbad hominid. *Nature* **382**, 500–501 (1996).
16. Smith, P. *et al.* The late Middle Pleistocene upper third molar from Florisbad: metrics and morphology. *Trans. R. Soc. South Afr.* **70**, 233–244 (2015).
17. Grün, R. & Stringer, C. Direct dating of human fossils and the ever-changing story of human evolution. *Quat. Sci. Rev.* **322**, 108379 (2023).

18. Grine, F. E., Holt, S., Brink, J. S. & du Plessis, A. Enamel pearls: Their occurrence in recent human populations and earliest manifestation in the modern human lineage. *Arch. Oral Biol.* **101**, 147–155 (2019).
19. Bamford, M. K. & Henderson, Z. L. A reassessment of the wooden fragment from Florisbad, South Africa. *J. Archaeol. Sci.* **30**, 637–650 (2003).
20. Coetzee, L. & Brink, J. S. Fossil oribatid mites (Acari, Oribatida) from the Florisbad Quaternary deposits, South Africa. *Quat. Res.* **59**, 246–254 (2003).
21. Grobler, J. C. & Loock, N. J. The Florisbad mineral spring : its characteristics and genesis : conclusions. *Navorsinge Van Nas. Mus. Res. Natl. Mus.* **5**, 477–483 (1988).
22. Fourie, H. The influence of degree of water saturation on the growth of obligate hydrophytic vegetation. (University of the Free State, Bloemfontein, 2012).
23. Toffolo, M. B. Pleistocene archaeology and environments of the Free State, South Africa. *Azania Archaeol. Res. Afr.* **59**, 317–351 (2024).
24. Toffolo, M. B., Brink, J. S., van Huyssteen, C. & Berna, F. A microstratigraphic reevaluation of the Florisbad spring site, Free State Province, South Africa: Formation processes and paleoenvironment: Toffolo et al. *Geoarchaeology* **32**, 456–478 (2017).
25. Rubidge, B. S. & Brink, J. S. Preliminary-survey of the extent and nature of the Pleistocene sedimentary deposits at Florisbad, South Africa: conclusions. *Navorsinge Van Nas. Mus. Bloemfontein* **5**, 73–75 (1985).
26. Scott, L. & Nyakale, M. Pollen indications of Holocene palaeoenvironments at Florisbad spring in the central Free State, South Africa. *The Holocene* **12**, 497–503 (2002).
27. Pinder, R. C. Using multiple luminescence chronometers to establish the chronology of the Middle Stone Age site of Florisbad, South Africa. Unpublished PhD thesis, Aberystwyth University (2021).
28. Toffolo, M. B., Brink, J. S. & Berna, F. Bone diagenesis at the Florisbad spring site, Free State Province (South Africa): Implications for the taphonomy of the Middle and Late Pleistocene faunal assemblages. *J. Archaeol. Sci. Rep.* **4**, 152–163 (2015).
29. van Zinderen Bakker, E. M. A pollen analytical investigation of the Florisbad deposits (South Africa). in *Proceedings of the Third-Pan-African Congress on Prehistory, Livingstone, 1955* (ed. Clarke, J. D.) 56–67 (Chatto and Windus, London, 1957).
30. van Zinderen Bakker, E. M. Middle Stone Age palaeoenvironments at Florisbad (South Africa). *Palaeoecol. Afr.* **20**, 133–154 (1989).
31. van Zinderen Bakker, E. M. Archaeology and palynology. *Afr Archaeol Bull* **162**, 98–105 (1995).
32. Scott, L. *et al.* Palynology of Middle Stone Age spring deposits in grassland at the Florisbad hominin site, South Africa. *Rev. Palaeobot. Palynol.* **265**, 13–26 (2019).
33. Scott, L. *et al.* Late Quaternary palaeoenvironments in the central semi-arid region of South Africa from pollen in cave, pan, spring, stream and dung deposits. *Quat. Int.* **614**, 84–97 (2022).
34. Sachse, D., Radke, J. & Gleixner, G. δD values of individual n-alkanes from terrestrial plants along a climatic gradient – Implications for the sedimentary biomarker record. *Org. Geochem.* **37**, 469–483 (2006).
35. Seki, O. *et al.* Alkenone and boron-based Pliocene pCO2 records. *Earth Planet. Sci. Lett.* **292**, 201–211 (2010).

36. Diefendorf, A. F., Freeman, K. H., Wing, S. L. & Graham, H. V. Production of n-alkyl lipids in living plants and implications for the geologic past. *Geochim. Cosmochim. Acta* **75**, 7472–7485 (2011).
37. Sachse, D. *et al.* Molecular Paleohydrology: Interpreting the Hydrogen-Isotopic Composition of Lipid Biomarkers from Photosynthesizing Organisms. *Annu. Rev. Earth Planet. Sci.* **40**, 221–249 (2012).
38. Tipple, B. J. & Pagani, M. Environmental control on eastern broadleaf forest species' leaf wax distributions and D/H ratios. *Geochim. Cosmochim. Acta* **111**, 64–77 (2013).
39. Freimuth, E. J., Diefendorf, A. F., Lowell, T. V. & Wiles, G. C. Sedimentary n-alkanes and n-alkanoic acids in a temperate bog are biased toward woody plants. *Org. Geochem.* **128**, 94–107 (2019).
40. Carr, A. S. *et al.* Leaf wax n-alkane distributions in arid zone South African flora: Environmental controls, chemotaxonomy and palaeoecological implications. *Org. Geochem.* **67**, 72–84 (2014).
41. Herrmann, N. *et al.* Sources, transport and deposition of terrestrial organic material: A case study from southwestern Africa. *Quat. Sci. Rev.* **149**, 215–229 (2016).
42. Pancost, R. D. Biomarker carbon and hydrogen isotopes reveal changing peatland vegetation, hydroclimate and biogeochemical tipping points. *Quat. Sci. Rev.* **339**, 108828 (2024).
43. Baker, A., Routh, J. & Roychoudhury, A. N. Biomarker records of palaeoenvironmental variations in subtropical Southern Africa since the late Pleistocene: Evidences from a coastal peatland. *Palaeogeogr. Palaeoclimatol. Palaeoecol.* **451**, 1–12 (2016).
44. Garcin, Y. *et al.* Hydroclimatic vulnerability of peat carbon in the central Congo Basin. *Nature* **612**, 277–282 (2022).
45. Ficken, K. J., Li, B., Swain, D. L. & Eglinton, G. An n-alkane proxy for the sedimentary input of submerged/¯oating freshwater aquatic macrophytes. *Org. Geochem.* **31**, 745–749 (2000).
46. Ben Arous, E. *et al.* Humans in Africa's wet tropical forests 150 thousand years ago. *Nature* **640**, 402–407 (2025).
47. Liu, H. & Liu, W. Hydrogen isotope fractionation variations of n-alkanes and fatty acids in algae and submerged plants from Tibetan Plateau lakes: Implications for palaeoclimatic reconstruction. *Sci. Total Environ.* **695**, 133925 (2019).
48. Stock, W. D., Chuba, D. K. & Verboom, G. A. Distribution of South African C_3 and C_4 species of Cyperaceae in relation to climate and phylogeny. *Austral Ecol.* **29**, 313–319 (2004).
49. Tweedy, R. R. *et al.* African C3 grass n-alkane distributions & plant chemotaxonomy. *Org. Geochem.* **208**, 104994 (2025).
50. Vogel, J. C., Fuls, A. & Ellis, R. P. The geographical distribution of Kranz grasses in South Africa. *South Afr. J. Sci.* **74**, 209–215 (1978).
51. Boom, A., Carr, A. S., Chase, B. M., Grimes, H. L. & Meadows, M. E. Leaf wax n-alkanes and δ13C values of CAM plants from arid southwest Africa. *Org. Geochem.* **67**, 99–102 (2014).

52. Wintle, A. G. & Murray, A. S. A review of quartz optically stimulated luminescence characteristics and their relevance in single-aliquot regeneration dating protocols. *Radiat. Meas.* **41**, 369–391 (2006).
53. Kreutzer, S. *et al.* Luminescence: Comprehensive Luminescence Dating Data Analysis, R package version 1.1.0. 1.2.1 https://doi.org/10.32614/CRAN.package.Luminescence (2025).
