# Supplementary material for: Non-analog environments supported early Homo sapiens in southern Africa 200 000 years ago
Source: Commun Earth Environ. 2026 Oct 30;7(1):829. doi: 10.1038/s43247-026-04116-4 (PMC13722590; doi:10.1038/s43247-026-04116-4)
Supplement: Supplementary file 3 [file 43247_2026_4116_MOESM3_ESM.docx]

**Description of Additional Supplementary file**

File name: Supplementary Data 1

Description: Raw stable isotope data.
